# Polymer-tethered glyconanoparticle colourimetric biosensors for lectin binding: structural and experimental parameters to ensure a robust output†

**DOI:** 10.1039/d2ra06265h

**Published:** 2022-11-18

**Authors:** Julian Micallef, Alexander N. Baker, Sarah-Jane Richards, Douglas E. Soutar, Panagiotis G. Georgiou, Marc Walker, Matthew I. Gibson

**Affiliations:** Department of Chemistry, University of Warwick CV4 7AL UK m.i.gibson@warwick.ac.uk; Department of Physics, University of Warwick CV4 7AL UK; Division of Biomedical Sciences, Warwick Medical School, University of Warwick Gibbet Hill Road CV4 7AL Coventry UK

## Abstract

Glycan–lectin interactions play essential roles in biology; as the site of attachment for pathogens, cell–cell communication, and as crucial players in the immune system. Identifying if a new glycan (natural or unnatural) binds a protein partner, or if a new protein (or mutant) binds a glycan remains a non-trivial problem, with few accessible or low-cost tools available. Micro-arrays allow for the interrogation of 100's of glycans but are not widely available in individual laboratories. Biophysical techniques such as isothermal titration calorimetry, surface plasmon resonance spectrometry, biolayer interferometry and nuclear magnetic resonance spectroscopy all provide detailed understanding of glycan binding but are relatively expensive. Glycosylated plasmonic nanoparticles based on gold cores with polymeric tethers have emerged as biosensors to detect glycan–protein binding, based on colourimetric (red to blue) outputs which can be easily interpreted by a simple UV-visible spectrometer or by eye. Despite the large number of reports there are no standard protocols for each system or recommended start points, to allow a new user to deploy this technology. Here we explore the key parameters of nanoparticle size, polymeric tether length and gold concentration to provide some guidelines for how polymer-tethered glycosylated gold nanoparticles can be used to probe a new glycan/protein interactions, with minimal optimisation barriers. This work aimed to remove the need to explore chemical and nanoparticle space and hence remove a barrier for other users when deploying this system. We show that the concentration of the gold core is crucial to balance strong responses *versus* false positives and recommend a gold core size and polymer tether length which balances sufficient colloidal stability and output. Whilst subtle differences between glycans/lectins will impact the outcomes, these parameters should enable a lab user to quickly evaluate binding using minimal quantities of the glycan and lectin, to select candidates for further study.

## Abstract 2

Polymer tethered glycosylated gold nanoparticles are optimised to provide a starting point to evaluate glycan–lectin interactions.

## Graphical abstract

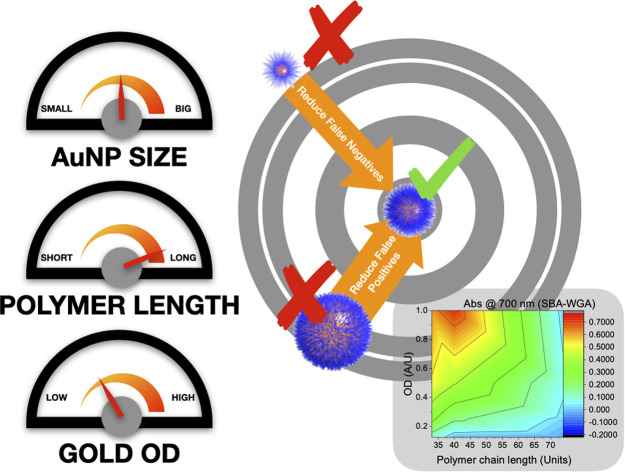

## Introduction

Glycan–protein interactions direct a huge range of biological recognition and signalling processes, spanning roles in immunology to pathogen invasion.^1–3^ For example, glycans are key binding motifs during the recognition of SARS-CoV-2 infection^4–7^ and the differential binding of hemagglutinins to sialic acid isomers is a key factor for influenza zoonosis from avian to humans.^8,9^ A key challenge in the dissection and discovery of glycan–lectin interactions is their intrinsically low affinity which is typically in the millimolar range.^10^ This leads to challenges in measuring the affinities requiring sensitive techniques such as isothermal titration calorimetry,^11^ nuclear magnetic resonance,^12^ microscale thermophoresis,^13^ and surface plasmon resonance^14^ (to name a few select examples) which are not always available and are relatively expensive.^11^ Due to the cluster glycoside effect,^10,15^ the multivalent display of glycans leads to a non-linear increase in affinity (or avidity) and hence observed affinities in the sub-nanomolar range can be realised, which has been widely exploited in the materials field using polymers,^16,17^ dendrimers,^18,19^ or particles.^20–24^ Carbohydrate micro-arrays^25^ which can contain 100's of distinct glycans are the primary tool for screening new glycan/lectin interactions. These use labelled (or secondary labelled) proteins *versus* glycans immobilised using amine or lipid^26–28^ anchors typically, but also polymers^29,30^ and emerging related technologies, such as bacteriophage-based solution assays.^31^ Whilst powerful, glycan arrays are not widely available and are suited to testing 10–100's of binding events, but not for rapid measurement at the bench.

Plasmonic nanoparticles based on gold (but also other metals^32^) are key components of lateral flow assays^33^ due to their intense red colouration and easy immobilisation of ligands, but they also show aggregation-dependent colourimetric responses. Mirkin and co-workers showed that gold nanoparticles functionalised with complementary DNA strands would aggregate, allowing a visible colour shift from red (dispersed) to blue (aggregated) due to the coupling of their surface plasmon resonance (SPR) bands.^34–36^ This is an appealing output, as the binding (caused by aggregation) can be seen by eye, or measured using UV-visible spectroscopy, which is widely available in laboratories, can be miniaturised and is low cost. Gold nanoparticles functionalised with glycans are therefore ideally suited for probing lectin-binding interactions as many (but not all) lectins have multiple binding sites enabling cross-linking.^37–41^ The installation of a glycan onto a gold surface is relatively simple using thiol–gold interactions. However, for any intended use in biosensing the colloidal stability of the resulting particles are crucial. Direct immobilisation of thio-glycosides onto gold particles leads to materials which bind their lectin partners, but control experiments demonstrate that the saline (or other buffer components) can also lead to aggregation (and hence colour change); a false-positive response.^42^ Richards *et al.* used RAFT (reversible additional fragmentation transfer) polymerisation to vary the length of a glycosylated polymeric linker from 10 to 70 repeat units, and immobilised these onto 60 nm gold particles.^43^ It was shown that the shortest polymers led to rapid aggregation in all conditions (due to colloidal instability) but that the longest failed to aggregate, even though the lectin partners were still binding,^44^ due to the too high colloidal stability of the particles. This highlights a key challenge in using aggregation to trigger an appealing and easy-to-read output, *i.e.*, the underpinning nanoparticle interface dictates the extent of response, as well as all the other features of linker length, particle size, and particle concentration. A wide range of conditions and linkers have been reported in the literature (including by ourselves). For example Baker *et al.* showed how 16 or 40 nm AuNPs with various polymer coating lengths, and glycan densities, impacted the lectin sensing outcomes in lateral flow formats.^45^ Other linkers such as poly(ethylene glycol) and oligo(ethylene) glycol have also been widely used.^46–49^

There exist many reports of polymer-tethered glyconanoparticles but the exact experimental details and design criteria which lead to a useful output vary significantly between each case, with the need to optimise first. Therefore, there remains a barrier to deployment of this useful biosensing tool in early-stage screening. Considering this, and the potential value of guidelines to enable a new user to dissect a glycan/protein interaction with minimal infrastructure and minimal optimisation, we set out to simplify the experimental and synthetic parameters to enable a robust glycosylated nanoparticle response to lectin binding using polymer-coated nanoparticles. Here we use (easy to obtain from commercial precursors) telechelic poly(*N*-hydroxyethyl acrylamide) tethers to capture the glycan and to immobilise onto the gold nanoparticles, based on our own developmental work using this tool. A range of gold nanoparticle cores were used, from 17–83 nm, and crucially the concentration of the gold (the optical density) were varied, which had a large impact on outputs. From these experiments we propose a set of conditions (polymer length, gold core, gold concentration, protein concentration) to enable a new user to obtain a robust response and minimise the chances of a false positive/negative, by removing the need for extensive optimisation.

## Results and discussion

**Fig. 1:**
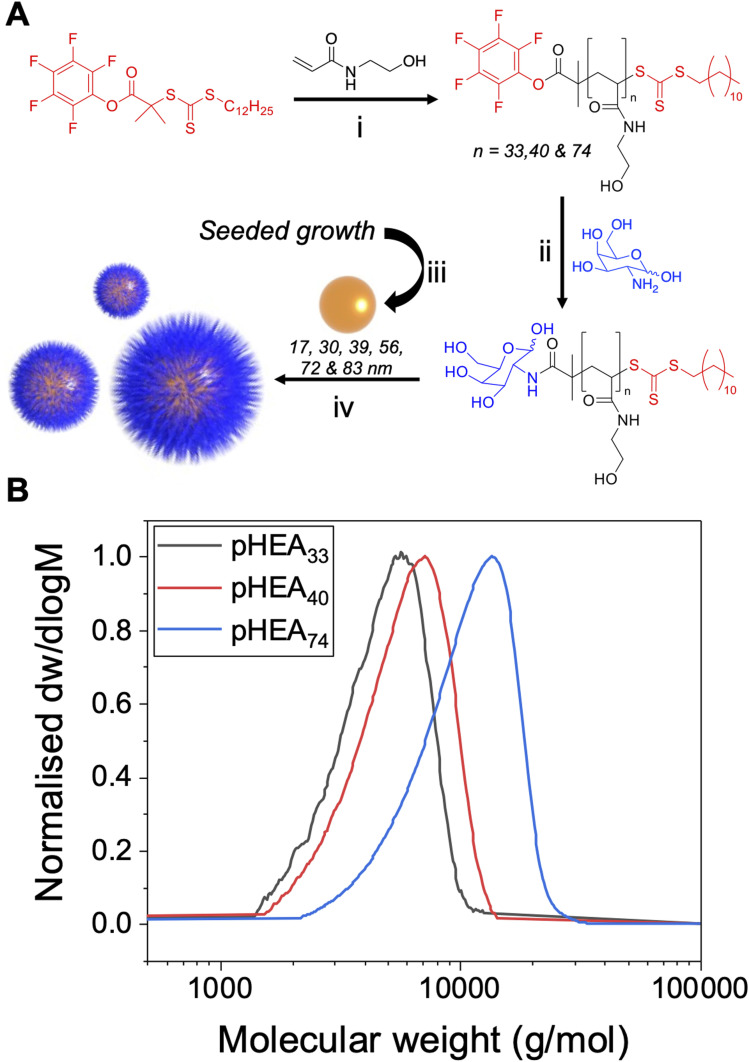
Polymer and gold nanoparticle synthesis. (A) Synthesis of PFP-terminated polymers. (i) ACVA, 70 °C, N_2_(g), MeOH/toluene (1 : 1); (ii) galactosamine, DMF, TEA, 50 °C; (iii) AuHCl_4_·3H_2_O, sodium citrate; (iv) galactosylated PHEA in water and preformed AuNPs; (B) molecular weight distributions from size exclusion chromatography of PHEAs in DMF.

The primary aim of this work was to systematically vary the experimental conditions of lectin-binding to polymer-tethered glyconanoparticles to provide a core set of parameters which give reliable signal outputs. This will allow other users to minimise the nano/chemical space which is required to be studied, enabling users to deploy these techniques to dissect glycan/protein binding, without the need for expensive infrastructure such as micro-arrays. Both polymer chain length and gold particle core are well known to impact the aggregation outcomes, hence a library of particles were prepared to allow systematic investigation of these variables.^21,44,45,50^ A series of poly(*N*-hydroxyethyl acrylamide) (pHEA) polymers were prepared using reversible addition–fragmentation chain transfer (RAFT) polymerisation to produce polymers with predictable polymer chain length and low dispersity using previously reported methods,^43,51^Fig. 1. The RAFT agent 2-(dodecylthiocarbonothioylthio)-2-methylpropionic acid pentafluorophenyl ester (PFP-DMP) was employed to allow installation of an amino-glycan at the ω-chain end, and to anchor onto gold surfaces using the masked thiol at the α-terminus. PHEA was chosen for its solubility and colloidal stability when immobilised on AuNPs, and as an acrylamide (rather than (meth)acrylate) it is hydrolytically stable.^33,43,52^ This modular approach allows the commonly used amino or azido terminated glycans to be easily captured on the chain end (the azide requires an intermediate alkyne-immobilisation step).^51^ This panel of polymers was functionalised with galactosamine by displacement of the PFP group, and characterised by SEC (size exclusion chromatography), ^1^H, ^13^C and ^19^F NMR (Table 1), showing narrow molecular weight distributions.

**Table 1:** Polymers synthesised

Polymer	[M] : [CTA] (−)	Conversion^a^ (%)	*M*_*n*_ (NMR)^a^ (g mol^−1^)	*M*_*n*_ b (g mol^−1^)	DP^b^ (−)	*Đ*_M_ b (−)
pHEA_33_	13	98	4100	4300	33	1.18
pHEA_40_	18	97	5600	5100	40	1.20
pHEA_74_	43	96	9800	9100	74	1.24

a Determined by ^1^H-NMR in D_2_O based on ratio of C***H***C***H***_2_ polymer backbone hydrogens to RAFT agent end-group.

b Determined by SEC in DMF + 5 mM NH_4_BF_4_ using poly(methyl methacrylate) (PMMA) standards.

**Fig. 2:**
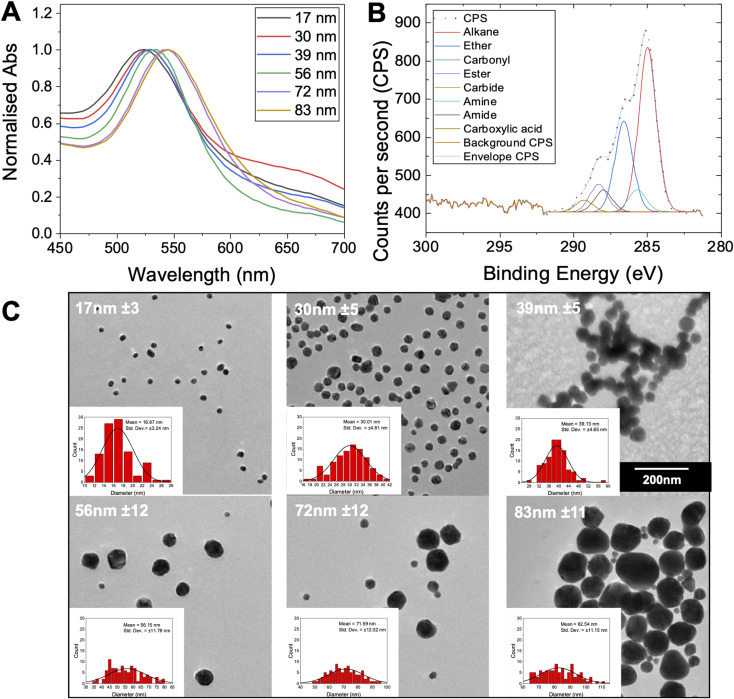
Gold nanoparticle characterisation. (A) UV-vis spectra for the gold nanoparticles synthesised; (B) representative XPS C 1s characterisation of Gal-PHEA_33_@AuNP_39_; (C) representative TEM images of the gold nanoparticles with their mean diameter indicated. Insets are the associated size histograms of a sample of 100 particles. All particles were imaged at same magnification.

To produce a panel of gold nanoparticle cores, a seeded growth mechanism was used.^53^ In brief, citrate stabilised 5–10 nm seed AuNPs were prepared by reduction of HAuCl_4_ with sodium citrate. These seeds were then used to prepare sequentially larger particles using the seeded growth method of Bastús *et al.*^53^ All AuNPs were characterised by dynamic light scattering (DLS), transmission electron microscopy (TEM) and UV-visible spectroscopy (UV-vis) (Fig. 2 and Table 2). These particles were subsequently coated with the polymers in Table 1, and excess polymer removed by centrifugation/re-suspension cycles, to give a library of 18 glycosylated nanoparticles. X-ray photoelectron spectroscopy (XPS) (Fig. 2B) provided confirmation that the polymer ligands (amide peaks) are present on the nanoparticles, but unlike other analytical techniques only requires microgram quantities so is ideally suited to these particles.

**Table 2:** Gold nanoparticle library

AuNP generation number and size	*λ*_SPR_ a (nm)	Abs_SPR_/Abs_450_	Diameter		
			UV-vis^b^ (nm)	DLS^c^ (nm)	TEM^d^ (nm)
1–17 nm	522	1.61	14	24 ± 0.3	17 ± 3
2–30 nm	524	1.66	30	60 ± 0.2	30 ± 5
4–39 nm	527	1.84	40	49 ± 0.4	39 ± 5
6–56 nm	532	2.01	52	49 ± 0.2	56 ± 12
8–72 nm	539	2.11	66	66 ± 0.1	72 ± 12
9–83 nm	543	2.13	72	76 ± 0.2	83 ± 11

a SPR peak (maximum) from UV-vis.

b Diameter estimated from UV-vis estimated using the method of Haiss *et al*.^54^

c Diameter from DLS.

d Diameter from TEM (sample of 100 particles) of each AuNP size collected. Error is ± SD from three repeat measurements for DLS and for a sample of 100 particles for TEM.

**Fig. 3:**
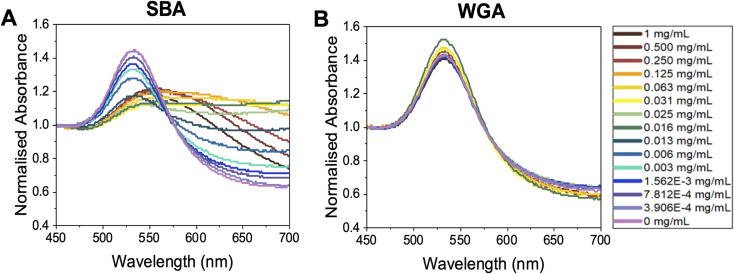
Representative UV-vis spectroscopy of OD_537_ (UV_max_) = 0.5 Gal-pHEA_33_@AuNP_56_ in response to (A) SBA and (B) WGA.

With this panel of glycosylated nanoparticles to hand, our aim was to first map how the polymer molecular weight and optical density (standard measure of concentration) of the gold nanoparticles impact the biosensing outcomes. The optical density of each gold nanoparticle system was taken as the maximum absorbance in UV-vis spectroscopy, *i.e.*, the absorbance of the SPR peak, typically around 520 nm. For these glycoparticles to be a reproducible tool, the potential for false positives (unwanted aggregation under the wrong conditions) or false negatives (no aggregation under the correct conditions) needs to be eliminated. To probe all the variables of signal generation, each polymer gold nanoparticle system was tested against a panel of lectins and controls. An optical density of 0.5 was chosen as a starting point as this has been used by ourselves in previous studies (the effect of varying OD is studied later in the present manuscript).^6,55,56^Fig. 3 shows dose-dependent UV-visible response curves of Gal-pHEA_33_@AuNP_56_ against SBA (soybean agglutinin) and WGA (wheat germ agglutinin). SBA has high affinity towards terminal *N*-acetylgalactosamine (GalNAc)^57,58^ (present on the polymer chain ends after galactosamine addition). WGA preferentially binds terminal sialic acids and *N*-acetylglucosamine (GlcNAc).^59^Fig. 3A and B shows typical binding curves with an overall increase in Abs_700_ when SBA is added, and only small changes when WGA is added. These small changes are crucial in the context of avoiding false positives as comparing both dose-dependent curves (not just the target lectin) are essential to allow real binding events to be distinguished from false positives. Fig. 3A highlights a further key consideration of using aggregation biosensors known as the hook effect (also known as prozone effect). At high concentrations of lectin, the signal begins to decrease due to saturation of the particle surface, so lectins only bind 1 particle (*i.e.* no cross-linking occurs) and hence no aggregation therefore no signal. This is an important consideration to ensure lectins are tested in a concentration regime which allows true positive effects to be probed. An apparent dissociation constant of 8 μg mL^−1^ (66 nM) was observed for SBA (Fig. 3A). This highlights the cluster glycoside effect enhancement and is similar to previously reported values for galactosylated gold nanoparticles verses SBA.^43^ It should be noted that these are only apparent and not the true *K*_d_ due to the multivalent/multivalent nature of the interaction.^60^

**Fig. 4:**
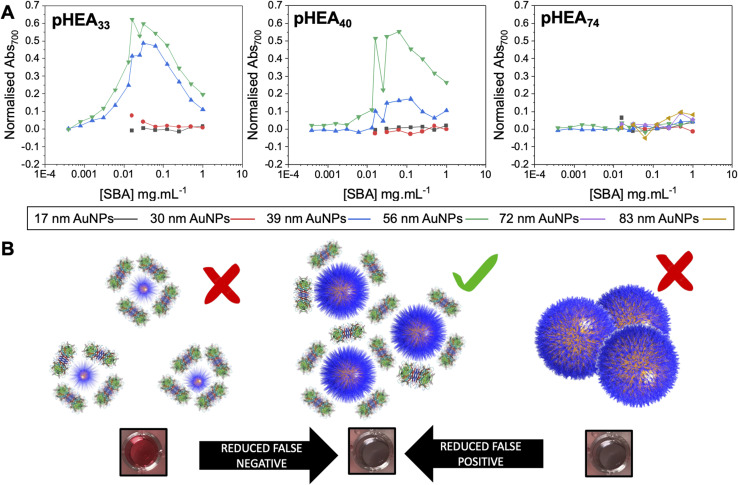
Demonstration of impact of excess analyte (lectin) on outputs. (A) Dose-responses of Abs_700_ (based on eqn (1) in ESI†) as a function of nanoparticle size for the three polymer lengths with lectin gradients. (B) Schematic of how particle size and lectin concentration impacts output.

With the above data to hand, the entire library could be considered, Fig. 4A (complete UV-vis spectra are in ESI†). For some of the polymer-coated nanoparticles, aggregation was observed by eye prior to lectin addition (obvious blue colouration) and confirmed by UV-vis and DLS, indicating these particles were not colloidally stable and so could not be taken forward. This partial aggregation was observed where the polymer chain length was insufficient to stabilise the larger particles; Gal-pHEA_33_@AuNP_72_, Gal-pHEA_33_@AuNP_83_ and Gal-pHEA_40_@AuNP_83_ systems. Hence, these systems were excluded from all future tests as their aggregation was unrelated to the lectin and instead were inherent to the AuNPs' colloidal stability. Furthermore, in our initial testing of the 17 and 30 nm core AuNPs, no aggregation was observed by UV-vis after addition of SBA (shown in Fig. S30 and S31†), this is demonstrated in Fig. 4B. This experiment is essential as it confirms previous reports that the size of the nanoparticle is key to generate a robust output and that smaller nanoparticles do not aggregate under these conditions, however this does not mean that binding is not occurring, which can be validated by surface sensitive techniques for example.^44^

The next consideration was the impact of the optical density (concentration) of the gold nanoparticle solutions. Reported values for the OD varies between publications, or is reported in molar concentrations of the gold, which is more challenging to determine. However, the OD (defined as the absorbance at the SPR band peak (UV_max_), and is typically in 520–540 nm range) is a more convenient measure which is not dependent on accurate size estimates from electron microscopy, for example. Schofield *et al.* used OD = 0.5 gold (with a PEG linker) to detect *Ricinus communis* agglutinin (RCA120).^61^ The same team reported cholera toxin detection with (without PEG linker) lactose using OD = 0.35 AuNPs.^47^ Conversely, peanut agglutinin was detected using AuNPs with ODs as low as 0.1.^62^ Baker *et al.* reported the impact of AuNP OD on outputs in lateral flow devices (where the hook effect would also be an issue) to detect plant lectins and the SARS-CoV-2 spike protein.^45,63^ Fairbanks *et al.* showed dose-dependent responses of sialic acid functional gold nanoparticles to hemagglutinins and intact virus using 3 nM gold, which showed an OD of ∼0.02.^41^ These select examples highlight the range of concentrations used and the versatility of the gold nanoparticles, but also the need for a ‘start point’ for new measurements.

**Fig. 5:**
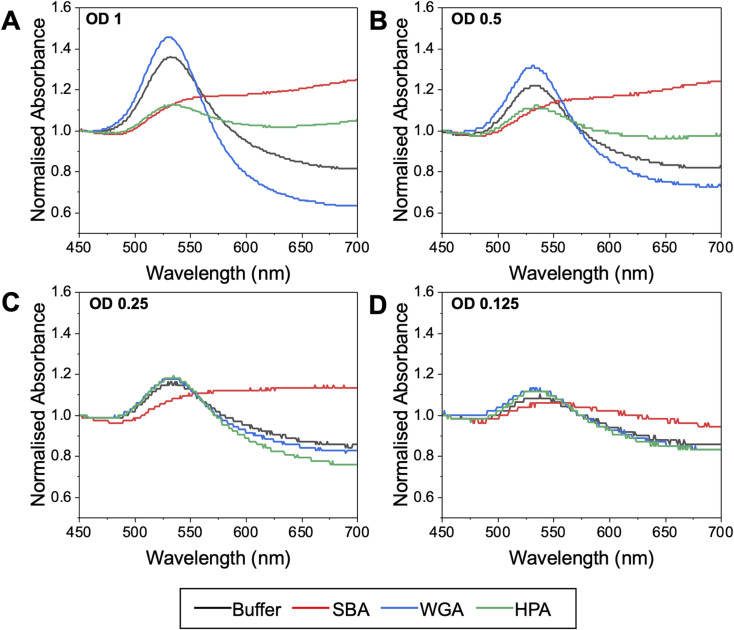
Normalised UV-visible spectra of Gal-pHEA_33_@AuNP_56_ nanoparticles after addition of SBA, HPA or WGA. All lectins used at 0.1 mg mL^−1^. (A) OD_537_ = 1; (B) OD_537_ = 0.5; (C) OD_537_ = 0.25; (D) OD_537_ = 0.125. Note, the starting OD indicated was taken from non-normalised data. Normalisation is essential to allow comparison of datasets.

The effect of OD is essential to allow reproducibility between experiments (as an easily accessible measurement), plus the probe concentration is expected to have a large impact on the signal outcomes. Fig. 5 shows the response of Gal-pHEA_33_@AuNP_56_ at OD_537_ values of 0.125 to 1, which is representative of concentrations suitable for use in these assays, balancing not saturating the detector and retaining resolution. The 56 nm AuNPs were selected as the starting particles for these experiments, as our previous results suggested AuNPs close to 60 nm give the strongest responses to SBA binding.^43,56^ In these experiments the lectin concentration was fixed at 0.1 mg mL^−1^. A third lectin was also introduced here as a further control – HPA (*Helix Pomatia* Agglutinin) also binds GalNAc residues (like SBA). HPA has a different architecture to SBA and so ensures the results span a reasonable range of lectins. At the highest OD_537_ (=1) (Fig. 5A) HPA and SBA both gave strong, but different magnitudes of, responses (increases in Abs_700_), indicative of binding to the terminal GalNAc units. Godula *et al.* have shown how the structural differences between SBA and HPA leads to altered binding in microarrays, with SBA showing significantly stronger propensities to crosslink compared to HPA.^30^ Despite these true positive responses, there was a small shift in the UV-vis spectra upon addition of WGA too. If this was the interrogation of an unknown lectin (or a known lectin against a new, or unnatural, glycan) this small shift may incorrectly be interpreted as a real binding response. This signal is likely due to non-specific fouling^64^ of the particle surface, and hence the ‘negative’ signal seen, rather than true aggregative responses. Lowering the AuNP concentration to OD_537_ = 0.5 (Fig. 5B) retained the SBA/HPA shifts, but reduced the unwanted WGA response, showing how the gold probe concentration (not just the lectin, polymer or AuNP size) is essential for optimisation. At OD_537_ = 0.25 (Fig. 5C), SBA produced a strong response, but HPA showed significantly weaker responses, as did WGA. At OD_537_ = 0.125 (Fig. 5D) there was a weak response to SBA only. It is also important to note the UV-vis traces become noisier at low OD's, which can be addressed by fitting (not undertaken here to ensure representative data is displayed) or increasing resolution of each collection step.

This data shows the that too high OD (particle concentration) can produce false positives, but that too low an OD can give false negatives. Based on this observation, an OD_peak_ = 0.5 seems to provide the optimal balance to allow specific interactions to be dissected using this tool, based on the panel of proteins used here. We would note that lower ODs can be successfully used, and we are not suggesting otherwise, depending on the binding partner, and this is intended only as a guide for this system.

**Fig. 6:**
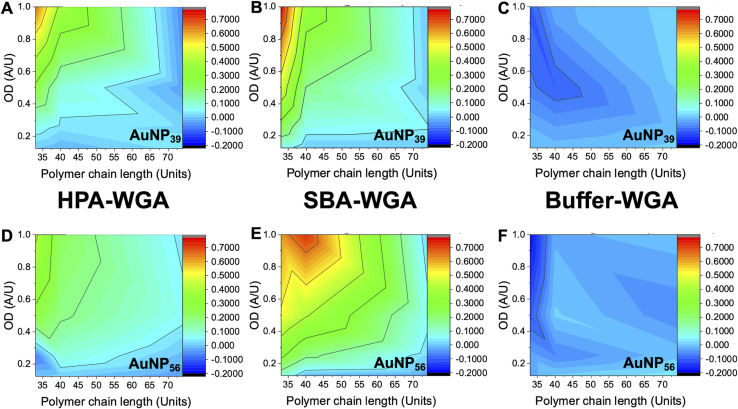
Heatmaps showing how the difference in absorbance at 700 nm for HPA *versus* WGA (HPA-WGA), SBA *versus* WGA (SBA-WGA) and buffer *versus* WGA (buffer-WGA) at varying polymer lengths and AuNP sizes; AuNP_39_ with (A) HPA-WGA; (B) SBA-WGA and (C) buffer-WGA. AuNP_56_ with (D) HPA-WGA; (E) SBA-WGA; (F) buffer-WGA.

The above data confirmed how the optical density (concentration) of the gold probe as well as the nature of the polymeric coating and nanoparticle size each play a role in ensuring a glycan–lectin binding output is a true positive. Based on the above, a larger number of experiments were undertaken to allow both the OD and polymer tether length on the overall signal response to be evaluated in a sequential manner, spanning maximum chemical and formulation space. Based on the data above only the 39 nm and 56 nm AuNP systems were taken forward as smaller nanoparticles failed to give strong lectin binding responses. Aggregation (Abs_700_) was measured for OD_max_ between 0.125 and 1, and polymer chain lengths of 33–74 for all 3 lectins (HPA, SBA, WGA). In each case, data is reported as the signal of the desired lectin bindings (SBA/HPA) with the WGA change (if any) subtracted to allow the discriminatory potential to be seen. In the case of WGA, this was minus the buffer only control. The results are plotted in Fig. 6, allowing visualisation of the regions giving higher (red) or lower (blue) signals. Strong and clear aggregation of SBA was observed for the Gal-pHEA_33_@AuNP_39_, Gal-pHEA_40_@AuNP_39_, Gal-pHEA_33_@AuNP_56_, and Gal-pHEA_40_@AuNP_56_ systems without any stability issues and minimal binding of the non-target lectin, WGA. HPA displayed similar binding to the Gal-PHEA_33_@AuNP_39_ and Gal-pHEA_40_@AuNP_39_ systems when compared to SBA. In contrast, HPA binding to the Gal-pHEA_33_@AuNP_56_ and pHEA_40_@AuNP_56_ systems was shown to be significantly weaker than that observed for SBA, allowing the binding of these two lectins to be more distinguishable from one another when the diameter of the nanoparticles was increased from 39 to 56 nm.

The 56 nm AuNPs showed essentially the same trends to the 39 nm AuNPs above. However, differences between SBA and HPA were more obvious in the 56 nm systems with the greatest difference in absorbance at 700 nm for SBA and WGA at ∼0.8 a.u compared to ∼0.7 a.u for HPA and WGA for the 39 nm AuNPs *versus* (Fig. 6). Hence, while the 39 nm AuNP systems demonstrated stronger binding, the 56 nm AuNP systems could better differentiate between the binding of SBA or HPA and WGA.^29^ The 56 nm AuNPs in general also showed less aggregation towards WGA when compared to a buffer double negative control making it easier to distinguish binding of SBA and HPA when compared to WGA (Fig. 6A and B). This is possibly due to some non-specific surface binding of WGA (*i.e.* nanoparticle corona^65^) as seen when BSA is used as a blocking agent to decrease aggregation in the buffer.^6^ Some non-specific binding will always occur, but this itself does not appear to lead to aggregation (and so does not impact the signal in this sensing platform).

Although smaller nanoparticles (below 39 nm) were excluded from our mapping (as above data suggested weaker binding) experiments were undertaken and it is important to comment on these. Some binding of SBA was observed with the Gal-pHEA_33_@AuNP_17_ (OD_522_ = 1) system, as well as some binding of HPA with the Gal-pHEA_74_@AuNP_17_ (OD_524_ = 1), Gal-pHEA_74_@AuNP_17_ (OD_524_ = 0.5), and Gal-pHEA_33_@AuNP_30_ (OD_527_ = 1) systems. As with any multivalent system, some cross-linking will occur on binding, at certain ODs. We reiterate that our aim is to establish robust conditions which allow testing of lectins to get reliable results but cannot rule out that aggregation will still occur in other platforms. For example, using directly conjugated glycans (no polymer linker) Fairbanks and co-workers found 20 nm cores to be optimal for heat label enterotoxin detection.^46^

Considering the results above, we propose (below) the following starting points to enable a user to rapidly assemble a nanoparticle system to interrogate their glycan/protein of choice. It should be noted that we do not recommend a specific conjugation chemistry here. Our choice of the pentafluorophenyl terminated polymer is deliberate, as this allows amino- or azido-glycans to be captured (in one, or two steps^51,56^), as these are commonly found as linkers in carbohydrate synthesis. We have not explored commercial polymer linkers here, as the versatility of the PFP-PHEA polymer in this application has been validated, and it can be easily synthesised from all commercial materials in a single step. Therefore, we recommend a start point as:

• 60 nm spherical gold (which is commercially available).

• Poly(*N*-hydroxyethyl acrylamide) with a number average degree of polymerisation close to 40 (5000 g mol^−1^).

• OD_540_ = 0.5 for the gold nanoparticle colloid.

• Starting lectin concentration of 0.1 mg mL^−1^. 2.01 mmol HEPES buffer containing 0.8 mmol CaCl_2_. This is important as many lectins require calcium (or other M^2+^ ions) and in standard phosphate buffers, these can precipitate calcium phosphate which leads to turbidity and can negatively impact measurements. The polymer coating is essential to provide stability in the saline.

• Read the UV-vis output after 30 minutes. Very long periods (*e.g.* 4 hours) can lead to reduced signal, as the cross-linked particles/lectin can precipitate.

This simplified workflow is suitable to enable small scale testing of new glycans, or new proteins where complete microarray (using 100's of samples) is not appropriate to allow investigation without moving to techniques such as SPR or ITC which can be complicated and require relatively expensive infrastructure.

## Conclusions

Glycosylated gold nanoparticles are widely known in the scientific literature and the impact of each component (core, polymer length, analyte, concentration) has been shown to be important. However, there does not exist a set of guidelines, to allow a new user to deploy a polymer-tethered glycosylated gold nanoparticle for monitoring binding to a lectin. Here we provide a step-by-step study of how each part of the nanoparticle formulation impacts binding outcomes with the aim of focusing on a single set of recommended conditions which can be deployed to enable any user to obtain initial binding data, removing the burden of a complete chemical and nanoscopic space exploration. We synthesised a small panel (guided by our previous experience of using these materials) of polymer-tethered glycosylated gold nanoparticles. RAFT polymerisation was used to obtain telechelic poly(*N*-hydroxyethyl acrylamide), displaying *N*-acetyl galactosamine at one terminus and a thiol at the other for immobilisation onto gold nanoparticles. Gold cores from 15–60 nm were evaluated, selected based on previous literature reports that these size ranges give colloidal stable dispersions, whilst having the desired colourimetric (aggregative) responses. Using this, we show that although smaller (sub 40 nm) gold cores can give aggregation responses, these could be weak in some cases, requiring higher concentrations of lectins to generate a response. Larger nanoparticles (approaching 60 nm) gave strong binding responses to the lectins, but it was observed that the concentration of gold used was crucial. At higher optical densities (concentration) non-specific binding led to small false positives, which in a single-lectin screen could be misleading, and too low concentrations of gold missed binding (false negatives). At an OD of 0.5 strong true positive signals could be seen. In any screen, there are obviously limitations and large oligosaccharides may impact the colloidal solutions, and OD can be varied significantly and a results still obtained, for example. We provide a series of suggested ‘starting points’ to enable a new user to use this approach without needing to explore wide chemical space.

## Data availability

The research data supporting this publication can be found in the ESI† and if there is addition information it can be found at https://wrap.warwick.ac.uk.

## Conflicts of interest

The authors declare no conflict of interest.

## Supplementary Material

RA-012-D2RA06265H-s001

## References

[cit1] Bertozzi C. R., Kiessling L. L. (2001). Chemical Glycobiology. Science.

[cit2] Lee Y. C., Lee R. T. (1995). Carbohydrate–Protein Interactions: Basis of Glycobiology. Acc. Chem. Res..

[cit3] Hudak J. E., Bertozzi C. R. (2014). Glycotherapy: New Advances Inspire a Reemergence of Glycans in Medicine. Chem. Biol..

[cit4] Guimond S. E., Mycroft-West C. J., Gandhi N. S., Tree J. A., Le T. T., Spalluto C. M., Humbert M. V., Buttigieg K. R., Coombes N., Elmore M. J. (2022). *et al.*, Synthetic Heparan Sulfate Mimetic Pixatimod (PG545) Potently Inhibits SARS-CoV-2 by Disrupting the Spike–ACE2 Interaction. ACS Cent. Sci..

[cit5] Clausen T. M., Sandoval D. R., Spliid C. B., Pihl J., Painter C. D., Thacker B. E., Glass C. A., Narayanan A., Majowicz S. A., Zhang Y. (2020). *et al.*, SARS-CoV-2 Infection Depends on Cellular Heparan Sulfate We Show That SARS-CoV-2 Spike Protein Interacts with Both Cellular Heparan Sulfate and Angiotensin-Converting Enzyme 2 (ACE2) through Its Receptor-Binding Domain (RBD). Docking Studies Suggest a Hep. Cell.

[cit6] Baker A. N., Richards S. J., Guy C. S., Congdon T. R., Hasan M., Zwetsloot A. J., Gallo A., Lewandowski J. R., Stansfeld P. J., Straube A. (2020). *et al.*, The SARS-COV-2 Spike Protein Binds Sialic Acids and Enables Rapid Detection in a Lateral Flow Point of Care Diagnostic Device. ACS Cent. Sci..

[cit7] Nguyen L., McCord K. A., Bui D. T., Bouwman K. A., Kitova E. N., Kumawat D., Daskan G. C., Tomris I., Han L., Chopra P. (2022). *et al.*, Sialic Acid-Containing Glycolipids Mediate Binding and Viral Entry of SARS-CoV-2. Nat. Chem. Biol..

[cit8] Childs R. A., Palma A. S., Wharton S., Matrosovich T., Liu Y., Chai W., Campanero-Rhodes M. A., Zhang Y., Eickmann M., Kiso M. (2009). *et al.*, Receptor-Binding Specificity of Pandemic Influenza A (H1N1) 2009 Virus Determined by Carbohydrate Microarray. Nat. Biotechnol..

[cit9] Vachieri S. G., Xiong X., Collins P. J., Walker P. A., Martin S. R., Haire L. F., Zhang Y., McCauley J. W., Gamblin S. J., Skehel J. J. (2014). Receptor Binding by H10 Influenza Viruses. Nature.

[cit10] Lundquist J. J., Toone E. J. (2002). The Cluster Glycoside Effect. Chem. Rev..

[cit11] Dam T. K., Brewer C. F. (2002). Thermodynamic Studies of Lectin–Carbohydrate Interactions by Isothermal Titration Calorimetry. Chem. Rev..

[cit12] Gimeno A., Valverde P., Ardá A., Jiménez-Barbero J. (2020). Glycan Structures and Their Interactions with Proteins. A NMR View. Curr. Opin. Struct. Biol..

[cit13] Jerabek-Willemsen M., André T., Wanner R., Roth H. M., Duhr S., Baaske P., Breitsprecher D. (2014). MicroScale Thermophoresis: Interaction Analysis and Beyond. J. Mol. Struct..

[cit14] Karamanska R., Clarke J., Blixt O., MacRae J. I., Zhang J. Q., Crocker P. R., Laurent N., Wright A., Flitsch S. L., Russell D. A. (2008). *et al.*, Surface Plasmon Resonance Imaging for Real-Time, Label-Free Analysis of Protein Interactions with Carbohydrate Microarrays. Glycoconjugate J..

[cit15] Richards S.-J., Gibson M. I. (2021). Toward Glycomaterials with Selectivity as Well as Affinity. JACS Au.

[cit16] Kanai M., Mortell K. H., Kiessling L. L. (1997). Varying the Size of Multivalent Ligands: The Dependence of Concanavalin A Binding on Neoglycopolymer Length. J. Am. Chem. Soc..

[cit17] Gerke C., Ebbesen M. F., Jansen D., Boden S., Freichel T., Hartmann L. (2017). Sequence-Controlled Glycopolymers *via* Step-Growth Polymerization of Precision Glycomacromolecules for Lectin Receptor Clustering. Biomacromolecules.

[cit18] Woller E. K., Cloninger M. J. (2002). The Lectin-Binding Properties of Six Generations of Mannose-Functionalized Dendrimers. Org. Lett..

[cit19] Zhang S., Xiao Q., Sherman S. E., Muncan A., Ramos Vicente A. D. M., Wang Z., Hammer D. A., Williams D., Chen Y., Pochan D. J. (2015). *et al.*, Glycodendrimersomes from Sequence-Defined Janus Glycodendrimers Reveal High Activity and Sensor Capacity for the Agglutination by Natural Variants of Human Lectins. J. Am. Chem. Soc..

[cit20] Marín M. J., Rashid A., Rejzek M., Fairhurst S. A., Wharton S. A., Martin S. R., McCauley J. W., Wileman T., Field R. A., Russell D. A. (2013). *et al.*, Glyconanoparticles for the Plasmonic Detection and Discrimination between Human and Avian Influenza Virus. Org. Biomol. Chem..

[cit21] Spain S. G., Albertin L., Cameron N. R. (2006). Facile *in Situ* Preparation of Biologically Active Multivalent Glyconanoparticles. Chem. Commun..

[cit22] Reichardt N.-C., Martín-Lomas M., Penadés S. (2016). Opportunities for Glyconanomaterials in Personalized Medicine. Chem. Commun..

[cit23] Marradi M., Chiodo F., García I., Penadés S., Whitesides G. M., Lee D. Y., Shin H., Pieters R. J., Fuente J. M. de la, Nishimura S.-H. (2013). *et al.*, Glyconanoparticles as Multifunctional and Multimodal Carbohydrate Systems. Chem. Soc. Rev..

[cit24] Baker A. N., Hawker-Bond G. W., Georgiou P. G., Dedola S., Field R. A., Gibson M. I. (2022). Glycosylated Gold Nanoparticles in Point of Care Diagnostics: From Aggregation to Lateral Flow. Chem. Soc. Rev..

[cit25] Bojar D., Meche L., Meng G., Eng W., Smith D. F., Cummings R. D., Mahal L. K. (2021). A Useful Guide to Lectin Binding: Machine-Learning Directed Annotation of 57 Unique Lectin Specificities. ACS Chem. Biol..

[cit26] Palma A. S., Feizi T., Childs R. A., Chai W., Liu Y. (2014). The Neoglycolipid (NGL)-Based Oligosaccharide Microarray System Poised to Decipher the *Meta*-Glycome. Curr. Opin. Chem. Biol..

[cit27] Feizi T., Fazio F., Chai W., Wong C. H. (2003). Carbohydrate Microarrays – a New Set of Technologies at the Frontiers of Glycomics. Curr. Opin. Struct. Biol..

[cit28] Rillahan C. D., Paulson J. C. (2011). Glycan Microarrays for Decoding the Glycome. Annu. Rev. Biochem..

[cit29] Godula K., Bertozzi C. R. (2012). Density Variant Glycan Microarray for Evaluating Cross-Linking of Mucin-like Glycoconjugates by Lectins. J. Am. Chem. Soc..

[cit30] Godula K., Bertozzi C. R. (2010). Synthesis of Glycopolymers for Microarray Applications *via* Ligation of Reducing Sugars to a Poly(Acryloyl Hydrazide) Scaffold. J. Am. Chem. Soc..

[cit31] Sojitra M., Sarkar S., Maghera J., Rodrigues E., Carpenter E. J., Seth S., Ferrer Vinals D., Bennett N. J., Reddy R., Khalil A. (2021). *et al.*, Genetically Encoded Multivalent Liquid Glycan Array Displayed on M13 Bacteriophage. Nat. Chem. Biol..

[cit32] Aberasturi D. J. de, Serrano-Montes A. B., Liz-Marzán L. M. (2015). Modern Applications of Plasmonic Nanoparticles: From Energy to Health. Adv. Opt. Mater..

[cit33] Bahadır E. B. and Sezgintürk M. K., Lateral Flow Assays: Principles, Designs and Labels, TrAC – Trends in Analytical Chemistry, Elsevier, 2016, pp. 286–306

[cit34] Mirkin C. A., Letsinger R. L., Mucic R. C., Storhoff J. J. (1996). A DNA-Based Method for Rationally Assembling Nanoparticles into Macroscopic Materials. Nature.

[cit35] Elghanian R., Storhoff J. J., Mucic R. C., Letsinger R. L., Mirkin C. A. (1997). Selective Colorimetric Detection of Polynucleotides Based on the Distance-Dependent Optical Properties of Gold Nanoparticles. Science.

[cit36] Eustis S., El-Sayed M. A. (2006). Why Gold Nanoparticles Are More Precious than Pretty Gold: Noble Metal Surface Plasmon Resonance and Its Enhancement of the Radiative and Nonradiative Properties of Nanocrystals of Different Shapes. Chem. Soc. Rev..

[cit37] Hone D. C., Haines A. H., Russell D. A. (2003). Rapid, Quantitative Colorimetric Detection of a Lectin Using Mannose-Stabilized Gold Nanoparticles. Langmuir.

[cit38] Schofield C. L., Haines A. H., Field R. A., Russell D. A. (2006). Silver and Gold Glyconanoparticles for Colorimetric Bioassays. Langmuir.

[cit39] Arnaud J., Audfray A., Imberty A. (2013). Binding Sugars: From Natural Lectins to Synthetic Receptors and Engineered Neolectins. Chem. Soc. Rev..

[cit40] Dam T. K., Brewer C. F. (2009). Lectins as Pattern Recognition Molecules: The Effects of Epitope Density in Innate Immunity. Glycobiology.

[cit41] Poonthiyil V., Nagesh P. T., Husain M., Golovko V. B., Fairbanks A. J. (2015). Gold Nanoparticles Decorated with Sialic Acid Terminated Bi-Antennary *N*-Glycans for the Detection of Influenza Virus at Nanomolar Concentrations. ChemistryOpen.

[cit42] Richards S.-J., Fullam E. C., Besra G. S., Gibson M. I. (2014). Discrimination between Bacterial Phenotypes Using Glyco-Nanoparticles and the Impact of Polymer Coating on Detection Readouts. J. Mater. Chem. B.

[cit43] Richards S.-J., Gibson M. I. (2014). Optimization of the Polymer Coating for Glycosylated Gold Nanoparticle Biosensors to Ensure Stability and Rapid Optical Readouts. ACS Macro Lett..

[cit44] Georgiou P. G., Baker A. N., Richards S.-J. J., Laezza A., Walker M., Gibson M. I. (2020). Tuning Aggregative *versus* Non-Aggregative Lectin Binding with Glycosylated Nanoparticles by the Nature of the Polymer Ligand. J. Mater. Chem. B.

[cit45] Baker A. N., Muguruza A. R., Richards S. J., Georgiou P. G., Goetz S., Walker M., Dedola S., Field R. A., Gibson M. I. (2022). Lateral Flow Glyco-Assays for the Rapid and Low-Cost Detection of Lectins – Polymeric Linkers and Particle Engineering Are Essential for Selectivity and Performance. Adv. Healthcare Mater..

[cit46] Poonthiyil V., Golovko V. B., Fairbanks A. J. (2015). Size-Optimized Galactose-Capped Gold Nanoparticles for the Colorimetric Detection of Heat-Labile Enterotoxin at Nanomolar Concentrations. Org. Biomol. Chem..

[cit47] Schofield C. L., Field R. A., Russell D. A. (2007). Glyconanoparticles for the Colorimetric Detection
of Cholera Toxin. Anal. Chem..

[cit48] Wang X., Ramström O., Yan M. (2009). A Photochemically Initiated Chemistry for Coupling Underivatized Carbohydrates to Gold Nanoparticles. J. Mater. Chem..

[cit49] Toyoshima M., Oura T., Fukuda T., Matsumoto E., Miura Y. (2010). Biological Specific Recognition of Glycopolymer – Modified Interfaces by RAFT Living Radical Polymerization. Polym. J..

[cit50] Won S., Richards S.-J., Walker M., Gibson M. I. (2017). Externally Controllable Glycan Presentation on Nanoparticle Surfaces to Modulate Lectin Recognition. Nanoscale Horiz..

[cit51] Richards S. J., Keenan T., Vendeville J. B., Wheatley D. E., Chidwick H., Budhadev D., Council C. E., Webster C. S., Ledru H., Baker A. N. (2021). *et al.*, Introducing Affinity and Selectivity into Galectin-Targeting Nanoparticles with Fluorinated Glycan Ligands. Chem. Sci..

[cit52] Richards S.-J., Biggs C. I., Gibson M. I. (2016). Multivalent Glycopolymer-Coated Gold Nanoparticles. Methods Mol. Biol..

[cit53] Bastús N. G., Comenge J., Puntes V. (2011). Kinetically Controlled Seeded Growth Synthesis of Citrate-Stabilized Gold Nanoparticles of up to 200 nm: Size Focusing *versus* Ostwald Ripening. Langmuir.

[cit54] Haiss W., Thanh N. T. K., Aveyard J., Fernig D. G. (2007). Determination of Size and Concentration of Gold Nanoparticles from UV–Vis Spectra. Anal. Chem..

[cit55] Pancaro A., Szymonik M., Georgiou P., Baker A. N., Walker M., Adriaensens P., Hendrix J., Gibson M. I., Nelissen I. (2021). The Polymeric Glyco-Linker Controls the Signal Outputs for Plasmonic Gold Nanorods Biosensors Due to Biocorona Formation. Nanoscale.

[cit56] Richards S.-J., Baker A. N., Walker M., Gibson M. I. (2020). Polymer-Stabilized Sialylated Nanoparticles: Synthesis, Optimization, and Differential Binding to Influenza Hemagglutinins. Biomacromolecules.

[cit57] Rao V. S. R., Lam K., Qasba P. K. (1998). Three Dimensional Structure of the Soybean Agglutinin-Gal/Galnac Complexes by Homology Modeling. J. Biomol. Struct. Dyn..

[cit58] Dam T. K., Gerken T. A., Brewer C. F. (2009). Thermodynamics of Multivalent Carbohydrate-Lectin Cross-Linking Interactions: Importance of Entropy in the Bind and Jump Mechanism. Biochemistry.

[cit59] Gallagher J. T., Morris A., Dexter T. M. (1985). Identification of Two Binding Sites for Wheat-Germ Agglutinin on Polylactosamine-Type Oligosaccharides. Biochem. J..

[cit60] Turnbull W. B., Precious B. L., Homans S. W. (2004). Dissecting the Cholera Toxin-Ganglioside GM1 Interaction by Isothermal Titration Calorimetry. J. Am. Chem. Soc..

[cit61] Schofield C. L., Mukhopadhyay B., Hardy S. M., McDonnell M. B., Field R. A., Russell D. A. (2008). Colorimetric Detection of Ricinus Communis Agglutinin 120 Using Optimally Presented Carbohydrate-Stabilised Gold Nanoparticles. Analyst.

[cit62] Hu X. Le, Jin H. Y., He X. P., James T. D., Chen G. R., Long Y. T. (2015). Colorimetric and Plasmonic Detection of Lectins Using Core–Shell Gold Glyconanoparticles Prepared by Copper-Free Click Chemistry. ACS Appl. Mater. Interfaces.

[cit63] Baker A. N., Richards S.-J., Pandey S., Guy C. S., Ahmad A., Hasan M., Biggs C. I., Georgiou P. G., Zwetsloot A. J., Straube A. (2021). *et al.*, Glycan-Based Flow-Through Device for the Detection of SARS-COV-2. ACS Sens..

[cit64] Ahmad A., Georgiou P., Hasan M., Pancaro A., Nelissen I., Gibson M. I. (2022). Polymer-Tethered Glycosylated Gold Nanoparticles Recruit Sialylated Glycoproteins into Their Protein Corona, Leading to Off-Target Lectin Binding. Nanoscale.

[cit65] Walczyk D., Bombelli F. B., Monopoli M. P., Lynch I., Dawson K. A. (2010). What the Cell “Sees” in Bionanoscience. J. Am. Chem. Soc..

