# Supplementary material for: Polymer-tethered glyconanoparticle colourimetric biosensors for lectin binding: structural and experimental parameters to ensure a robust output
Source: RSC Adv. 2022 Nov 18;12(51):33080–90. doi: 10.1039/d2ra06265h (PMC9672907; doi:10.1039/d2ra06265h)
Supplement: RA-012-D2RA06265H-s001 [file RA-012-D2RA06265H-s001.pdf]

Electronic Supplementary Material (ESI) for RSC Advances.
This journal is © The Royal Society of Chemistry 2022

Supporting Information for

Polymer-Tethered Glyconanoparticle Colourimetric Biosensors for Lectins: Structural and Experimental Parameters to Ensure a Robust Output

Julian Micallef,[a] Alexander N. Baker,[a] Sarah-Jane Richards,[a] Douglas E. Soutar,[a] Panagiotis G. Georgiou,[a] Marc Walker[b] and Matthew I. Gibson[a,c]*

a) Department of Chemistry, University of Warwick, UK, CV4 7AL

b) Department of Physics, University of Warwick, UK, CV4 7AL

c) Division of Biomedical Sciences, Warwick Medical School, University of Warwick, Gibbet Hill Road, CV4 7AL, Coventry, UK

Corresponding Author Email, m.i.gibson@warwick.ac.uk

Contents

Contents 2

Physical and Analytical Methods 4

NMR Spectroscopy 4

Mass spectrometry 4

FT-IR Spectroscopy 4

Size Exclusion Chromatography 4

X-ray Photoelectron Spectroscopy (XPS) 5

Dynamic Light Scattering 5

UV-vis Spectroscopy 6

Transmission Electron Microscopy 6

Materials 7

Synthetic Methods 8

Synthesis of 2-(dodecylthiocarbanothionylthio)-2-methyl propionic acid (DMP) 8

Synthesis of Pentafluorophenyl-2-dodecylthiocarbonothioylthio)-2-methylpropanoate (PFP-DMP) 9

Representative polymerization of 2-hydroxyethyl acrylamide 10

Representative DP40 Poly(N-hydroxyethyl acrylamide) glycan functionalisation 11

Citrate-stabilised 17 nm Gold Nanoparticle Synthesis[3] 12

Citrate-stabilised Gold Nanoparticle Seeded Growth Synthesis of 30-82 nm 12

Gold Nanoparticle Polymer Coating Functionalisation 13

2x HEPES buffer 13
Aggregation assay with varying lectin concentration and fixed AuNP OD 13
Aggregation assay with varying AuNP OD and fixed lectin concentration 13
Additional Data and Figures 15
Polymer Characterization 15
Nanoparticle Characterization 17
TEM Data 20
Analyzed XPS (x-ray Photoelectron Spectroscopy) data 26
Aggregation Assay Studies with SBA and WGA 32
Aggregation Assay Studies of SBA, WGA and HPA at low concentrations 38
Aggregation Assay Data for Varying OD 44
Aggregation Assay Heatmaps 56
References 68

Physical and Analytical Methods

NMR Spectroscopy

^{1}H-NMR, ^{13}C-NMR and ^{19}F-NMR spectra were recorded at 300 MHz, 400 MHz or 500 MHZ on a Bruker DPX-300, DPX-400 or DPX-500 spectrometer respectively, with chloroform-*d* ($CDCl_3$) or deuterium oxide (D_2O) as the solvent. Chemical shifts of protons are reported as δ in parts per million (ppm) and are relative to either $CDCl_3$ (7.26) or D_2O (4.79).

Mass spectrometry

Low resolution mass spectra (LRMS) were recorded on a Bruker Esquire 2000 spectrometer using electrospray ionisation (ESI). M/z values are reported in Daltons.

FT-IR Spectroscopy

Fourier Transform-Infrared (FT-IR) spectroscopy measurements were carried out using an Agilent Cary 630 FT-IR spectrometer, in the range of 650 to 4000 cm^{-1}.

Size Exclusion Chromatography

Size exclusion chromatography (SEC) analysis was performed on an Agilent Infinity II MDS instrument equipped with differential refractive index (DRI), viscometry (VS), dual angle light scatter (LS) and variable wavelength UV detectors. The system was equipped with 2 x PLgel Mixed D columns (300 x 7.5 mm) and a PLgel 5 µm guard column. The mobile phase used was DMF (HPLC grade) containing 5 mM NH_4BF_4 at 50 °C at flow rate of 1.0 $mL.min^{-1}$. Poly(methyl methacrylate) (PMMA) standards (Agilent EasyVials) were used for calibration between 955,000 – 550 $g.mol^{-1}$. Analyte samples were filtered through a nylon membrane with 0.22 μm pore size before injection. Number average molecular weights (M_n), weight average

molecular weights (M_w) and dispersities ($Đ_M = M_w/M_n$) were determined by conventional calibration and universal calibration using Agilent GPC/SEC software.

X-ray Photoelectron Spectroscopy (XPS)

The samples were attached to electrically-conductive carbon tape, mounted on to a sample bar and loaded in to a Kratos Axis Ultra DLD spectrometer which possesses a base pressure below 1 x 10^{-10} mbar. XPS measurements were performed in the main analysis chamber, with the sample being illuminated using a monochromated Al Kα x-ray source. The measurements were conducted at room temperature and at a take-off angle of 90° with respect to the surface parallel. The core level spectra were recorded using a pass energy of 20 eV (resolution approx. 0.4 eV), from an analysis area of 300 μm x 700 μm. The spectrometer work function and binding energy scale of the spectrometer were calibrated using the Fermi edge and $3d_{5/2}$ peak recorded from a polycrystalline Ag sample prior to the commencement of the experiments. In order to prevent surface charging the surface was flooded with a beam of low energy electrons throughout the experiment and this necessitated recalibration of the binding energy scale. To achieve this, the C-C/C-H component of the C 1*s* spectrum was referenced to 285.0 eV. The data were analysed in the CasaXPS package, using Shirley backgrounds and mixed Gaussian-Lorentzian (Voigt) lineshapes. For compositional analysis, the analyser transmission function has been determined using clean metallic foils to determine the detection efficiency across the full binding energy range.

Dynamic Light Scattering

Hydrodynamic diameters (D_h) and size distributions of particles were determined by dynamic light scattering (DLS) using a Malvern Zetasizer Nano ZS with a 4 mW He-Ne 633 nm laser module operating at 25 °C. Measurements were carried out at an angle of 173° (back scattering), and results were analysed using Malvern DTS 7.03 software. All determinations

were repeated 5 times with at least 10 measurements recorded for each run. D_h values were calculated using the Stokes-Einstein equation where particles are assumed to be spherical.

UV-vis Spectroscopy

Absorbance measurements were recorded on an Agilent Cary 60 UV-Vis Spectrophotometer and on a BioTek Epoch microplate reader.

Transmission Electron Microscopy

Dry-state stained TEM imaging was performed on a JEOL JEM-2100Plus microscope operating at an acceleration voltage of 200 kV. All dry-state samples were diluted with deionized water and then deposited onto formvar-coated copper grids.

Materials

All chemicals were used as supplied unless otherwise stated. *N*-Hydroxyethyl acrylamide (97 %), 4,4'-azobis(4-cyanovaleric acid) (98 %), mesitylene (reagent grade), triethylamine (> 99%), sodium citrate tribasic dihydrate (> 99 %), gold(III) chloride trihydrate (99.9%), potassium phosphate tri basic (≥ 98%, reagent grade), deuterium oxide (D_2O, 99.9%), deuterated chloroform ($CDCl_3$, 99.8%), diethyl ether ((≥ 99.8%, ACS reagent grade), sodium azide (≥ 99.5%, reagent plus grade), methanol (≥ 99.8%, ACS reagent grade), toluene (≥ 99.7%,), Tween-20 (molecular biology grade), HEPES, PBS tablets (dissolved in 200 mL of deionized water yields 0.01 M phosphate buffer, 0.0027 M potassium chloride and 0.137 M sodium chloride, pH 7.4), sucrose (Bioultra grade), carbon disulphide (≥ 99.8%), acetone (≥ 99%), 1-dodecane thiol (≥ 98%) and pentafluorophenol (≥ 99%, reagent plus) were purchased from Sigma-Aldrich. Galactosamine HCl was purchased from Carbosynth. Anhydrous trehalose was purchased from Alfa Aesar. DMF (>99%), 2-bromo-2-methyl propionic acid (98%) were purchased from Acros Organics. HPLC grade acetonitrile (≥ 99.8%), hexane fraction from petrol (lab reagent grade), DCM (99% lab reagent grade), sodium hydrogen carbonate (≥ 99%), ethyl acetate (≥ 99.7%, analytical reagent grade), sodium chloride (≥ 99.5%), calcium chloride, 40-60 petroleum ether (lab reagent grade), hydrochloric acid (~37%, analytical grade), glacial acetic acid (analytical grade) and magnesium sulphate (reagent grade) were purchased from Thermo Fisher Scientific.

Soybean agglutinin, *Ricinus Communis* Agglutinin I (RCA_{120}), Ulex Europaeus Agglutinin I and wheat germ agglutinin were purchased from Vector Laboratories.

Clear 96-well plates were purchased from Greiner Bio-one. Streptavidin (SA) biosensors were purchased from Forte Bio.

Distilled water used for buffers was MilliQ grade 18.2 mΩ resistance.

Synthetic Methods

Synthesis of 2-(dodecylthiocarbanothionylthio)-2-methyl propionic acid (DMP)

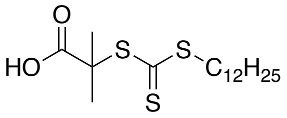

This was synthesised, according to a previously published procedure.[1,2] 2.37 mL (9.88 mmol) of 1-dodecane thiol was added dropwise to 2.13 g (10.03 mmol) of potassium phosphate tribasic monohydrate in 30 mL of acetone at RTP with stirring for 25 minutes. 2.09 g (27.39 mmol) of carbon disulfide was added and the mixture left for 10 minutes. 1.52 g (9.11 mmol) of 2-bromo-2-methyl propionic acid was then added and the solution left to stir at RTP for 16 hours. The solvent was then removed, and the crude product dissolved in 100 mL of 1 M hydrochloric acid and extracted through washings of 2 x 100 mL of DCM. The organic layer was then washed with distilled water, then brine, and dried with magnesium sulfate. The organic layer was filtered under gravity. The solvent was then removed under vacuum. The solid was tested against the reagents on TLC in 75:24:1 PET:DCM:Glacial acetic acid to confirm the reaction had gone to completion, and the solid was then recrystalised in n-hexane for 16 hours at -20 °C to produce a yellow, crystalline solid of mass 1.80 g (54.09%).[49] Structure was confirmed by $^{1}H/^{13}C$-NMR, ESI-MS and FT-IR. δ_H (400 MHz, $CDCl_3$) 3.28 (2H, t, *J*=7.5Hz, $SC\boldsymbol{H}_2CH_2$), 1.72-1.63 (8H, m, $C(C\boldsymbol{H}_3)_2$ and $SCH_2C\boldsymbol{H}_2$), 1.38-1.25 (18H, m, $(C\boldsymbol{H}_2)_9CH_3$), 0.88 (3H, t, *J*=6.5, $(CH_2)_9C\boldsymbol{H}_3$). δ_C (100 MHz, $CDCl_3$) 37.3 (1C, $S\boldsymbol{C}H_2$), 32.1-28.0 (9C, $SCH_2(\boldsymbol{C}H_2)_9$), 25.4 (2C, $C(\boldsymbol{C}H_3)_2$), 22.9 (1C, $\boldsymbol{C}H_2CH_3$), 14.3 (1C, $CH_2\boldsymbol{C}H_3$). 2955.8, 2916.6, 2847.7 (methyl and methylene), 1701.5 (ester C=O), 1457.4, 1435.0, 1412.7 (methyl and methylene), 1280.3 ($C(CH_3)_2$), 1064.2 (S-C(S)-S). m/z calculated at 364.16; found for ESI $[M+H]^+$ 365.1 and $[M+Na]^+$ 387.1 and $[M-H]^-$ 363.1.

Synthesis of Pentafluorophenyl-2-dodecylthiocarbonothioylthio)-2-methylpropanoate (PFP-DMP)

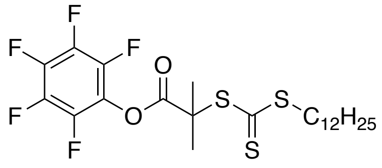

This was synthesised, according to a previously published procedure.[1,2] 1.47 g (4.04 mmol) of DMP, 1.42 g (7.42 mmol) of EDC and 0.88 g (7.20 mmol) of DMAP were dissolved in 160 mL of DCM and degassed with nitrogen for 30 minutes with stirring at RTP. 2.75 g (14.94 mmol) of pentafluorophenol was dissolved in 20 mL of DCM and added to the DMP mixture under nitrogen and stirred at RTP for 18 hours. This organic layer was subsequently washed with 2 washings of 100 mL of 3M hydrochloric acid, 2 washings of 100 mL of 1 M sodium hydrogen carbonate and 2 washings of 100 mL of 0.5 M sodium chloride. The organic layer was dried with magnesium sulfate and filtered under gravity. The solvent was then removed under vacuum and the solid attained recrystalised in ethyl acetate for 22 hours at -20 °C. The solids were then filtered under gravity to give a yellow crystalline solid of mass 0.59 g (27.45%).[49] Structure was confirmed by ^{1}H/^{13}C-NMR, ESI-MS and FT-IR. δ_H (400 MHz, $CDCl_3$) 3.31 (2H, t, *J*=7.5Hz, $SC\boldsymbol{H}_2CH_2$), 1.87 (2H, s, $SCH_2C\boldsymbol{H}_2$) 1.72-1.65 (6H, m, $C(C\boldsymbol{H}_3)_2$), 1.43-1.25 (18H, m, $(C\boldsymbol{H}_2)_9CH_3$), 0.88 (3H, t, *J*=6.5, $(CH_2)_9C\boldsymbol{H}_3$). δ_C (100 MHz, $CDCl_3$) 37.3 (1C, $S\boldsymbol{C}H_2$), 32.1-22.9 (9C, $SCH_2(\boldsymbol{C}H_2)_9$), 25.6 (2C, $C(\boldsymbol{C}H_3)_2$), 14.3 (1C, $CH_2\boldsymbol{C}H_3$). $\boldsymbol{\delta}_F$(400MHz, $CDCl_3$) -151.49 - -151.57 (2F, m, ortho-F), -157.69 - -157.80 (1F, t, J=23, para-F), -162.29 - -162.39 (2F, m, meta-F). 2924.1, 2853.3 (methyl and methylene), 1740.7 (ester C=O), 1518.9 (aromatic C=C or C-F), 1466.7 (methyl and methylene), 1231.9 ($C(CH_3)_2$). 1079.1 (S-C(S)-S). m/z calculated at 530.14; found for ESI $[M+Na]^+$ 553.1 and $[M-H]^-$ 529.4.

Representative polymerization of 2-hydroxyethyl acrylamide

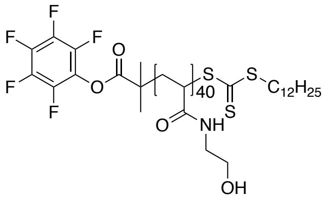

This was synthesised, according to a previously published procedure.[2] PHEA40 as representative example. 0.51 g (4.4024 mmol) of 2-hydroxyethyl acrylamide, 0.13 g (0.24 mmol) of PFP-DMP and 0.0165 g (0.11 mmol) of ACVA was added to 4 mL of 1:1 toluene:methanol and degassed under nitrogen for 30 minutes with stirring. The reaction was heated to 70 °C for 2 hours. After 2 hours, the vessel was quenched in ice cold water. The solvent was removed under vacuum. To purify, the crude product redissolved in the minimum amount of methanol. 25 mL of diethyl ether cooled in liquid nitrogen was added, precipitating out the product. The suspended product was centrifuged at 13 krpm for 2 minutes, and the liquid decanted off. The purification step was repeated once again. The purified product was redissolved in methanol which was then removed again under vacuum to attain a pure, dry product yellow powder, structure was confirmed by ^{1}H-NMR, GPC/SEC and FT-IR.

pHEA$_{40}$ - δ_H (400 MHz, D_2O) 3.86-2.94 (171H, m, $NHCH_2$, CH_2OH and SCH_2), 2.36-1.13 (157H, m, $CH_2CHC(O)$, $C(CH_3)_2$ and $(C\boldsymbol{H}_2)_7CH_2CH_3$), 0.71-0.56 (5H, m, CH_2CH_3). 3274.5 (Broad, OH), 3093.7, 2924.1 (C(O)NH and NH), 1638.2, 1541.3 (C(O)NH).

pHEA$_{33}$ - δ_H (400 MHz, D_2O) 3.87-2.92 (115H, m, $NHCH_2$, CH_2OH and SCH_2), 2.29-0.91 (118H, m, $CH_2CHC(O)$, $C(CH_3)_2$ and $(C\boldsymbol{H}_2)_7CH_2CH_3$), 0.71-0.58 (5H, m, CH_2CH_3).

pHEA$_{74}$ - δ_H (400 MHz, D_2O) 3.82-2.97 (331H, m, $NHCH_2$, CH_2OH and SCH_2), 2.21-0.91 (265H, m, $CH_2CHC(O)$, $C(CH_3)_2$ and $(C\boldsymbol{H}_2)_7CH_2CH_3$), 0.71-0.58 (5H, m, CH_2CH_3).

Representative DP40 Poly(N-hydroxyethyl acrylamide) glycan functionalisation

This was synthesised, according to a previously published procedure.[2] $pHEA_{40}$-Galactosamine as representative example. 0.21 g (0.040 mmol) of $pHEA_{40}$ and 0.02 g (0.004 mmol) of D-(+)-galactosamine hydrochloride was added to 20 mL DMF with 0.05M TEA. The reaction was stirred at 50 °C for 16 hours. The solvent was then removed under vacuum. The crude product was dissolved in the minimum amount of methanol and precipitated with 25 mL of diethyl ether. The suspended product was centrifuged at 13 krpm for 2 minutes, and the liquid decanted off. This washing step was repeated once more. The purified product was redissolved in methanol. The solvent was removed under vacuum to attain a pure, dry golden crystalline solid. ^{19}F-NMR was used to confirm reaction completion, structure was confirmed by ^{1}H-NMR and FT-IR.

Gal-pHEA$_{40}$ - δ_H (400 MHz, D_2O) 4.06-2.68 (219H, m, Glycan protons, CH_2OH and SCH_2), 2.38-0.88 (158H, m, $CH_2CHC(O)$, $C(CH_3)_2$, Glycan protons and $(C\boldsymbol{H}_2)_7CH_2CH_3$), 0.75-0.61 (5H, m, CH_2CH_3). δ_F (400 MHz, D_2O) No peaks. 3274.5 (Broad, OH), 3095.6, 2926.0 (C(O)NH and NH), 1638.2, 1546.8 (C(O)NH).

Gal-pHEA$_{33}$ - δ_H (400 MHz, D_2O) 4.10-2.67 (180H, m, Glycan protons, CH_2OH and SCH_2), 2.41-0.85 (132H, m, $CH_2CHC(O)$, $C(CH_3)_2$, Glycan protons and $(C\boldsymbol{H}_2)_7CH_2CH_3$), 0.74-0.60 (5H, m, CH_2CH_3). δ_F (400 MHz, D_2O) No peaks.

Gal-pHEA$_{74}$ - δ_H (400 MHz, D_2O) 4.04-2.67 (307H, m, Glycan protons, CH_2OH and SCH_2), 2.33-0.85 (211H, m, $CH_2CHC(O)$, $C(CH_3)_2$, Glycan protons and $(C\boldsymbol{H}_2)_7CH_2CH_3$), 0.75-0.63 (5H, m, CH_2CH_3). δ_F (400 MHz, D_2O) No peaks.

Citrate-stabilised 17 nm Gold Nanoparticle Synthesis[3]

0.03 g of gold (III) chloride trihydrate was added to 100 mL of distilled water and heated to reflux. 0.09 g (0.29 mmol) of sodium citrate tribasic dihydrate was dissolved in 2.92 mL of distilled water and was added to the gold solution. The solution was left under reflux for 30 minutes before being allowed to cool to RTP over 3 hours. The solution was centrifuged at 13 krpm for 20 minutes, and the pellet resuspended in 40 mL distilled water to give an Absorbance on UV-vis of 1 at 520 nm.[49] Size was determined by DLS, UV-vis and TEM.

Citrate-stabilised Gold Nanoparticle Seeded Growth Synthesis of 30-82 nm

35 nm gold nanoparticles were synthesised by a modified step growth method developed by Bastús *et al.*[3] A solution of 2.2 mM sodium citrate in Milli-Q water (150 mL) was heated under reflux for 15 min under vigorous stirring. After boiling had commenced, 1 mL of $HAuCl_4$ (25 mM) was injected. The colour of the solution changed from yellow to bluish gray and then to soft pink in 10 min, 1 mL was taken for DLS and UV/Vis analysis. Immediately after the synthesis of the Au seeds and in the same reaction vessel, the reaction was cooled until the temperature of the solution reached 90 °C. Then, 1 mL of a $HAuCl_4$ solution (25 mM) was injected. After 20 min, the reaction was finished. This process was repeated twice. After that, the sample was diluted by adding 85 mL of MilliQ water and 3.1 mL of 60 mM sodium citrate. This solution was then used as a seed solution, and three further portions of 1.6 mL of 25 mM $HAuCl_4$ were added with 20 min between each addition. Following completion of this step 1 mL was taken for DLS and UV/Vis analysis. The sample was diluted by adding 135 mL of MilliQ water and 4.9 mL of 60 mM sodium citrate. This solution was then used as the seed solution and the process repeated to attain all the desired gold nanoparticle sizes, using the dilution process to remove a 55 mL aliquot of the desired size particles. Final size was determined by DLS, UV-vis and TEM.

Gold Nanoparticle Polymer Coating Functionalisation

Stock solutions of each polymer (Gal-pHEA$_{33}$, Gal-pHEA$_{40}$, Gal-pHEA$_{74}$) were prepared by dissolving 10 mg of each separately into 1 mL of distilled water. 100 µL of each stock solution was added individually to 1 mL of OD 1 gold nanoparticles of the desired size. The nanoparticle solutions were left to functionalise overnight on a roller. The nanoparticle solutions were then centrifuged for 30 minutes at the speeds corresponding to their size, the super-natant removed, and the solutions re-diluted with distilled water. Two further washes were performed with 15-minute centrifugations at the corresponding speeds.[3,4]

2x HEPES buffer

0.48 g (2.01 mmol) of HEPES, 1.75 g (29.95 mmol) of sodium chloride, 88.8 mg of calcium chloride (0.80 mmol) and 4 µL (3.58 nmol) of TWEEN-20 were dissolved in 200 mL of distilled water and stirred for 30 minutes.

Aggregation assay with varying lectin concentration and fixed AuNP OD

An aggregation assay was prepared in 96 well plates by addition of 25 µL of functionalised gold nanoparticles of a given size to a serial dilution of 50 µL of 2 mg/mL SBA or WGA, diluting with 2x HEPES buffer, to attain 25 µL aliquots of concentrations 1, 0.5, 0.25, 0.125, 0.063, 0.031, and 0.015 mg/mL of SBA or WGA after dilution as well as a 25 µL HEPES buffer control. To give a total volume in the well of 50 µL.

Aggregation assay with varying AuNP OD and fixed lectin concentration

Stock solutions of lectins (0.1 mg/mL) were prepared by dissolving 0.5 mg of SBA, HPA or WGA in 5 mL of distilled water. 25 µL of SBA was added to 48 wells, this was repeated for the HPA, WGA and 2x HEPES buffer. To the wells, 25 µL of OD 2, 1, 0.5 and 0.25

functionalised gold nanoparticles of varying sizes and polymer functionalisation sizes were added.

Additional Data and Figures

Polymer Characterization

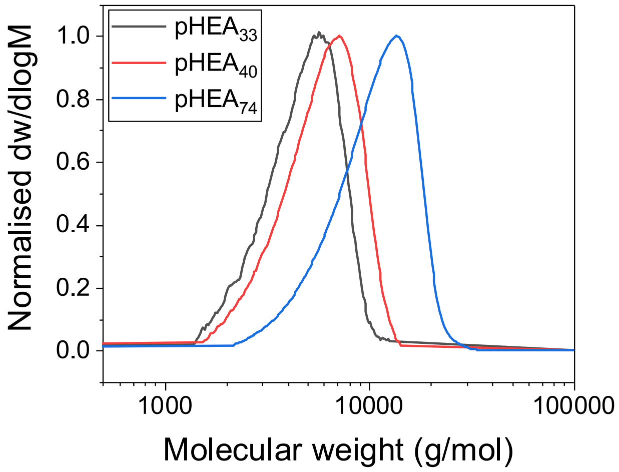

Figure S1. Normalised size exclusion chromatography RI molecular weight distributions of telechelic PHEA obtained in DMF verses PMMA standards.

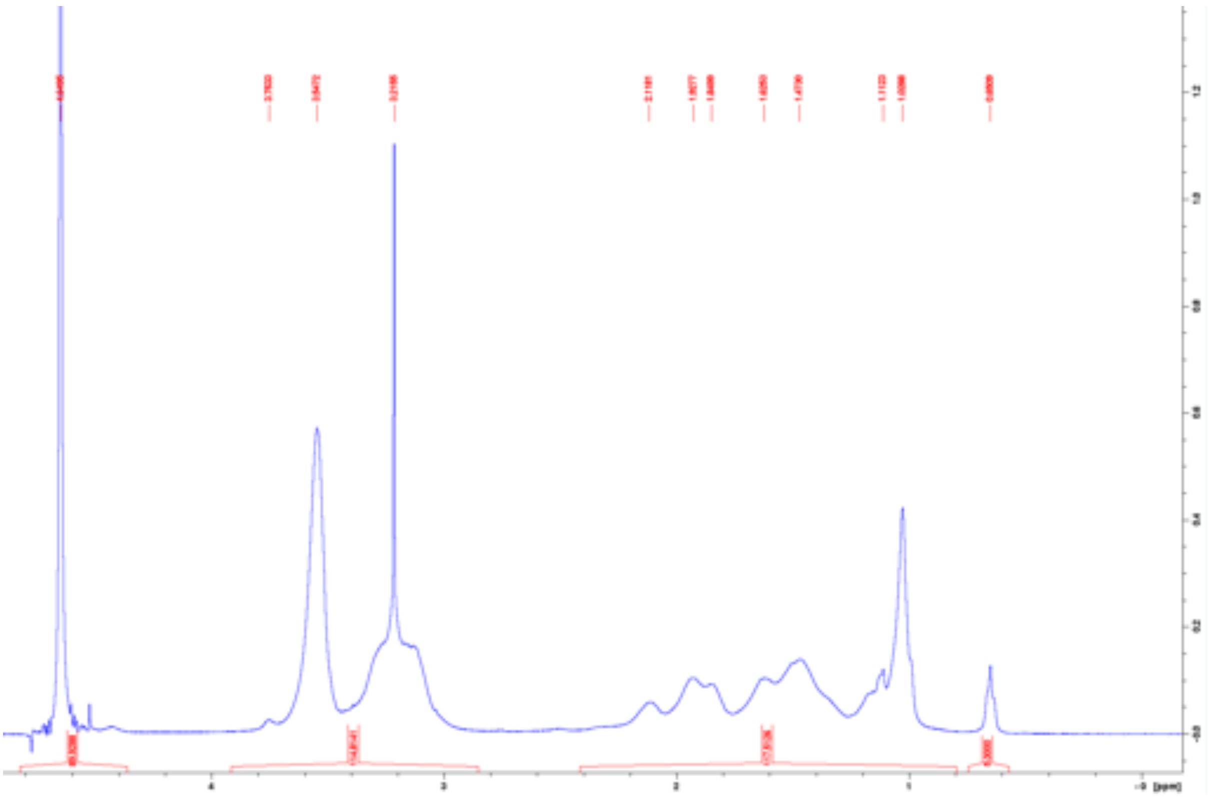

Figure S2. ^{1}H NMR spectrum of DP33 PHEA

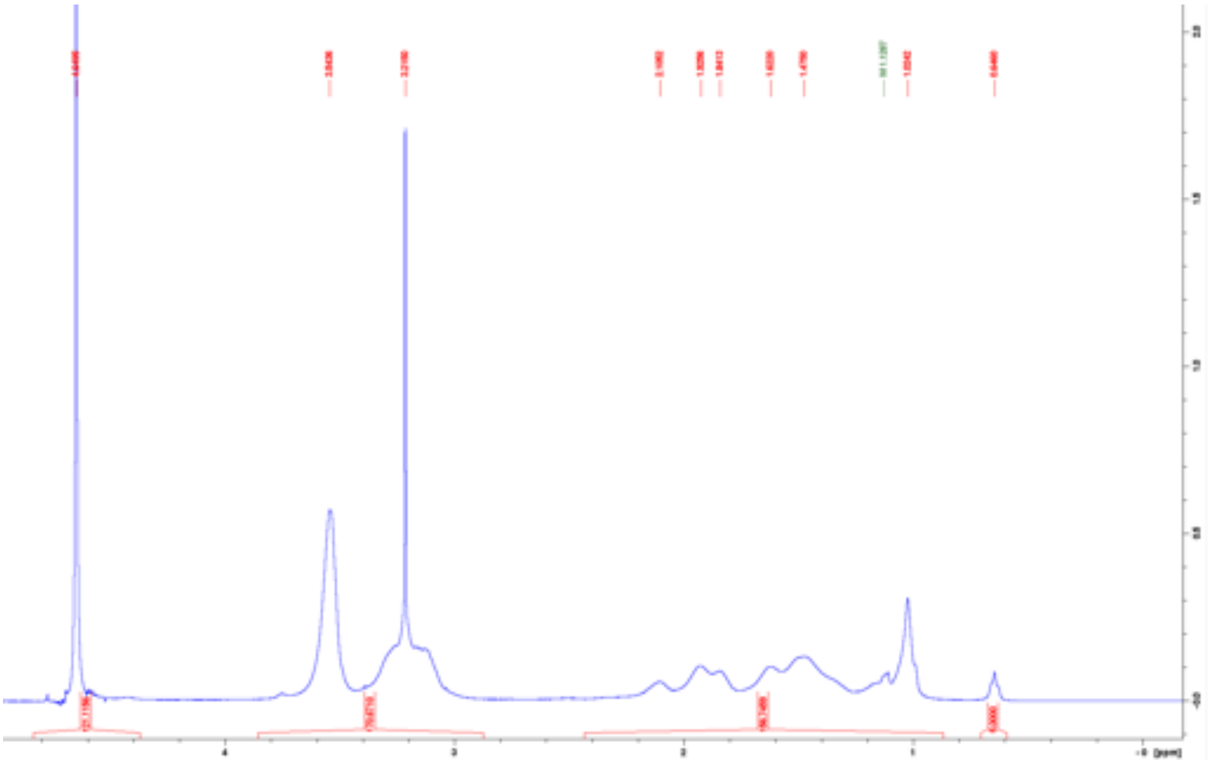

Figure S3. ^{1}H NMR spectrum of DP40 PHEA

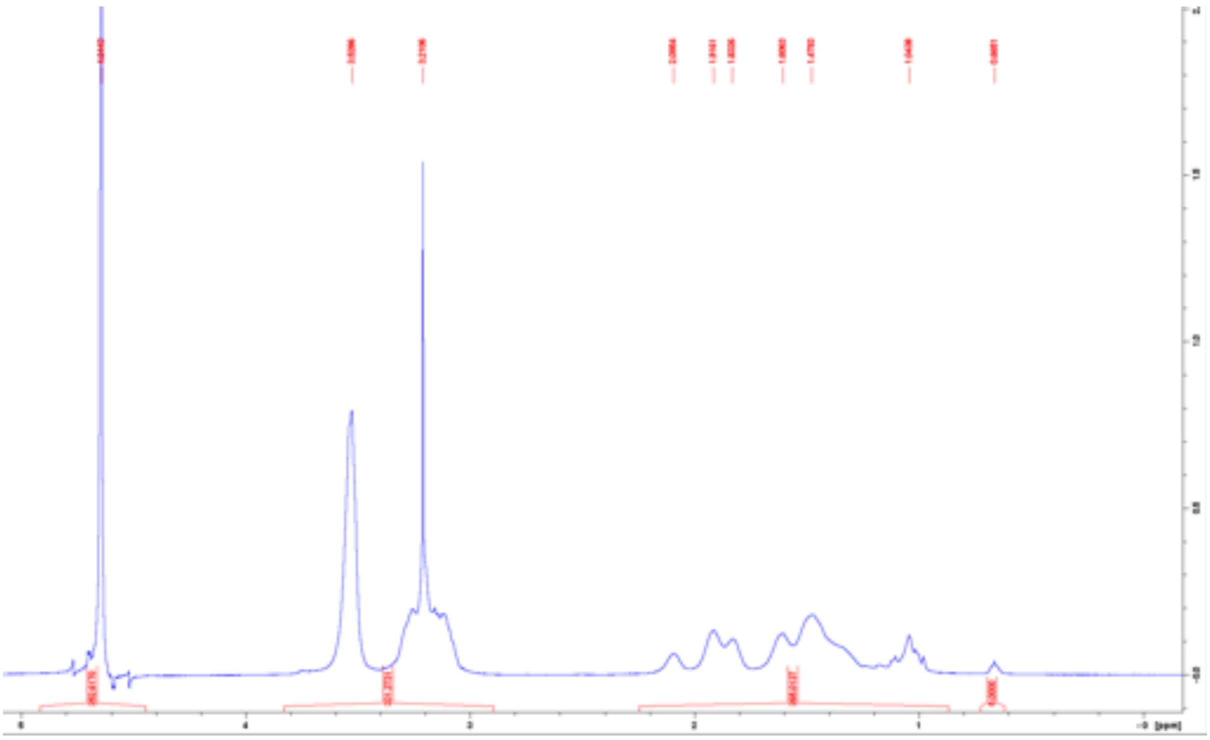

Figure S4. ^{1}H NMR spectrum of DP74 PHEA

Nanoparticle Characterization

The citrate-stabilized gold nanoparticles were characterized by DLS, UV/VIS and TEM. Example images and histogram analyses (from >100 particles) are shown below.

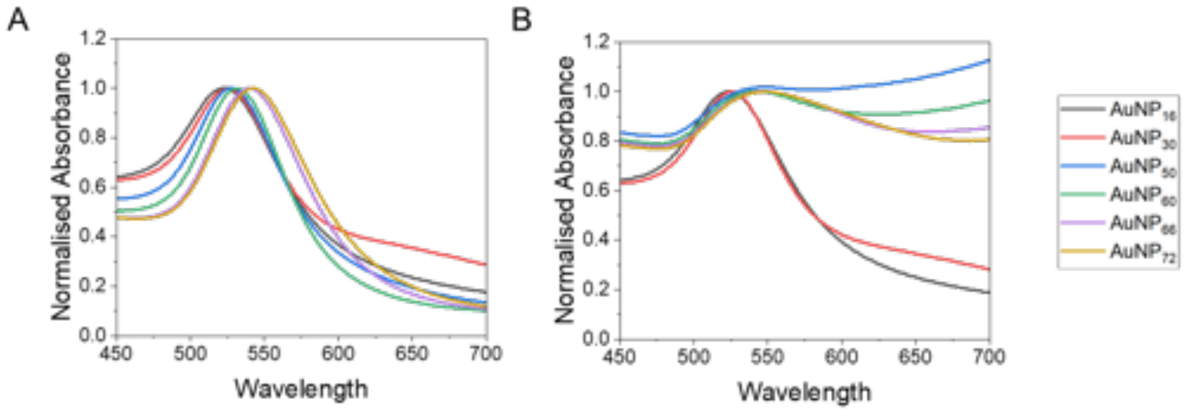

Figure S5. UV-vis characterization of uncoated $AuNP_{17,30,39,56,72,83}$ used in this study A) in water and B) in PBS.

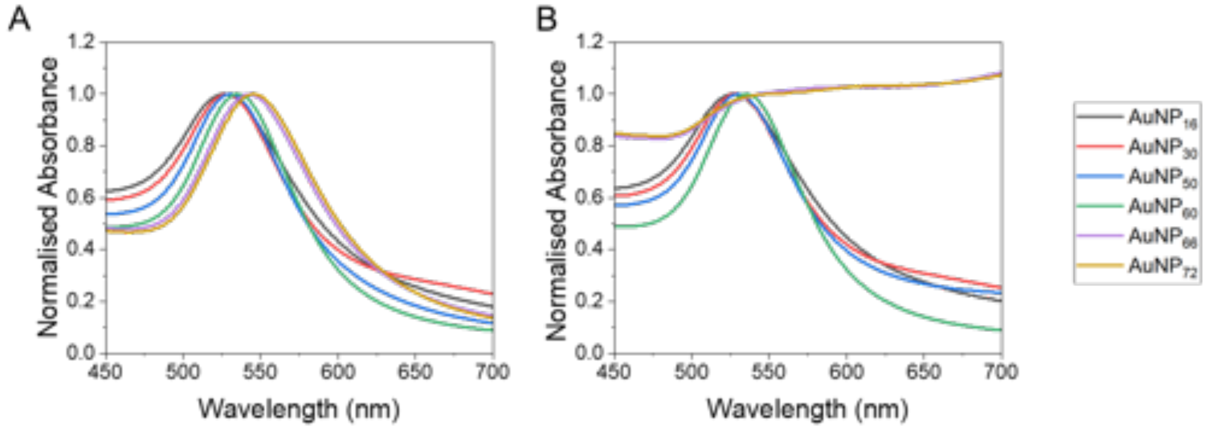

Figure S6. UV-vis characterization of $pHEA_{33}$ coated $AuNP_{17,30,39,56,72,83}$ used in this study A) in water and B) in PBS.

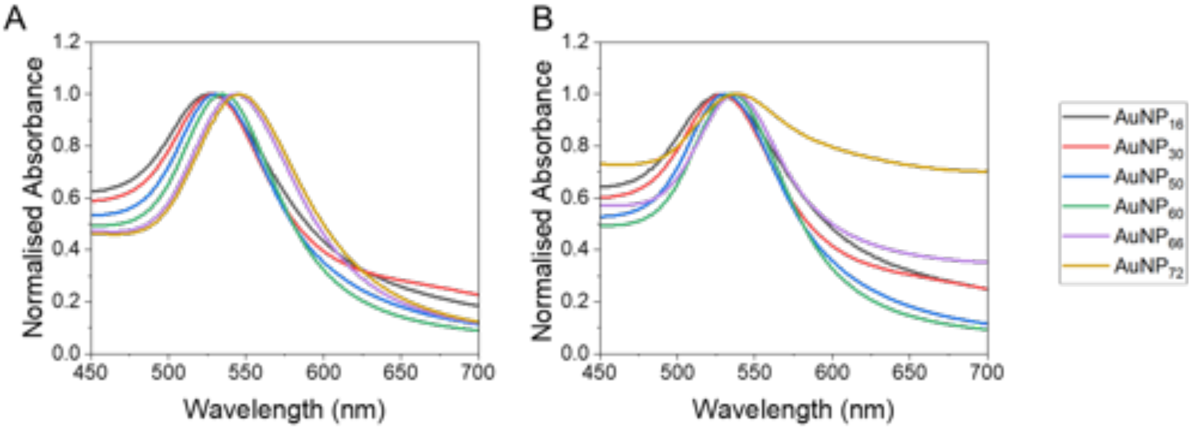

Figure S7. UV-vis characterization of $pHEA_{40}$ coated $AuNP_{17,30,39,56,72,83}$ used in this study A) in water and B) in PBS.

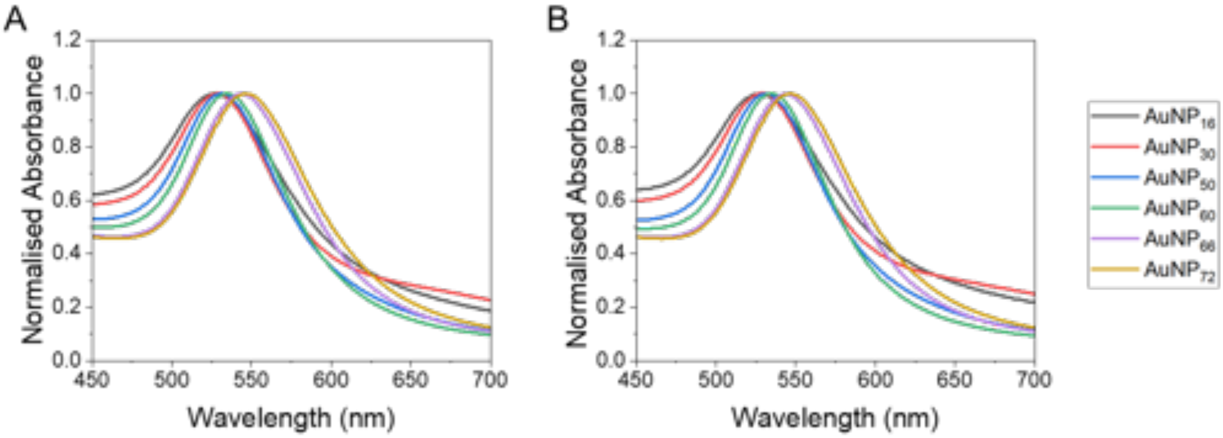

Figure S8. UV-vis characterization of $pHEA_{74}$ coated $AuNP_{17,30,39,56,72,83}$ used in this study A) in water and B) in PBS.

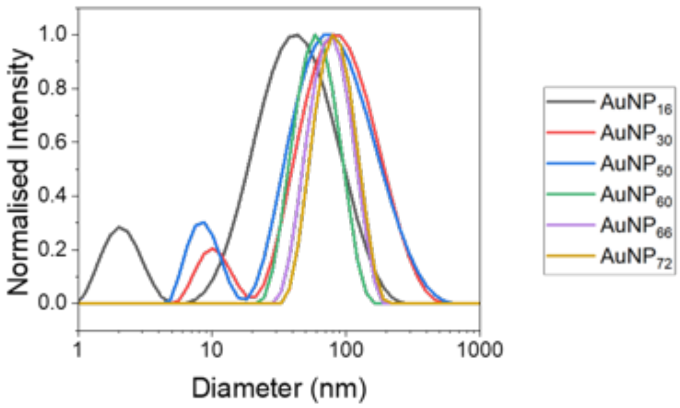

Figure S9. DLS characterization of uncoated AuNPs used in this study.

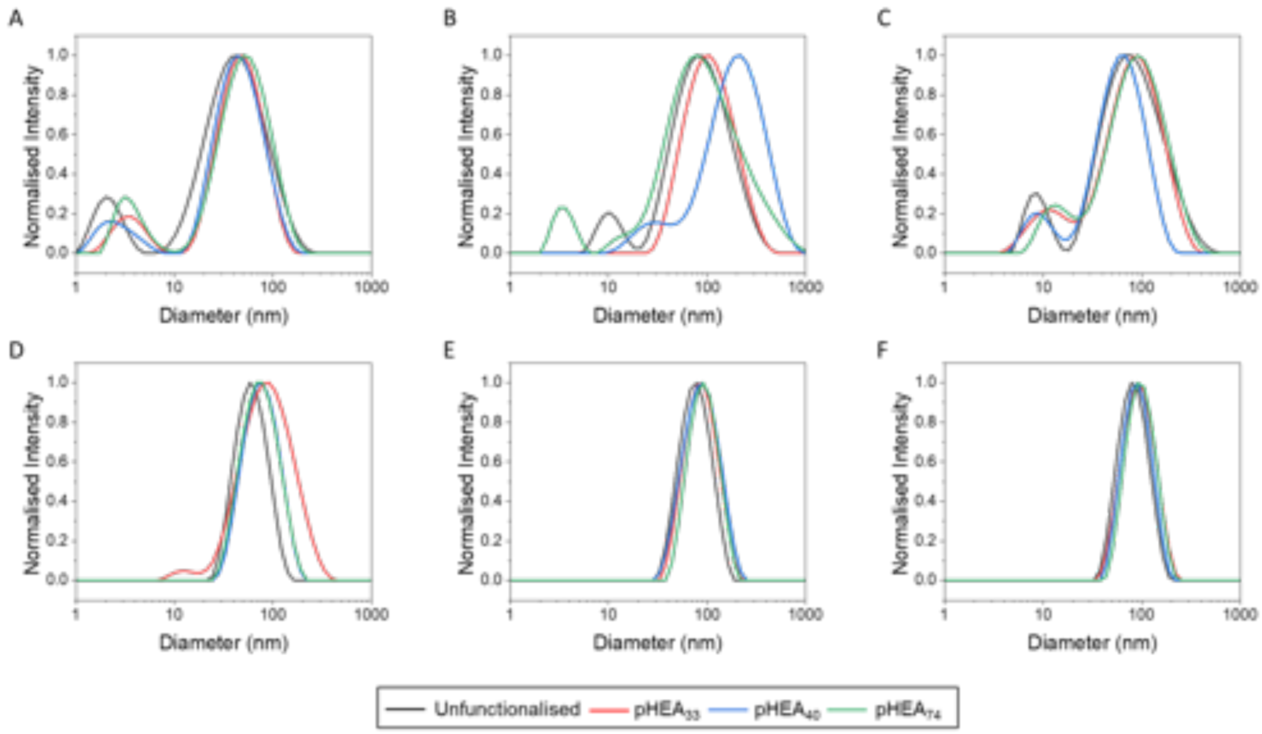

Figure S10. DLS characterization of uncoated and coated AuNPs of sizes A) 17 nm, B) 30 nm, C) 39 nm, D) 56 nm, E) 72 nm and F) 82nm used in this study.

TEM Data

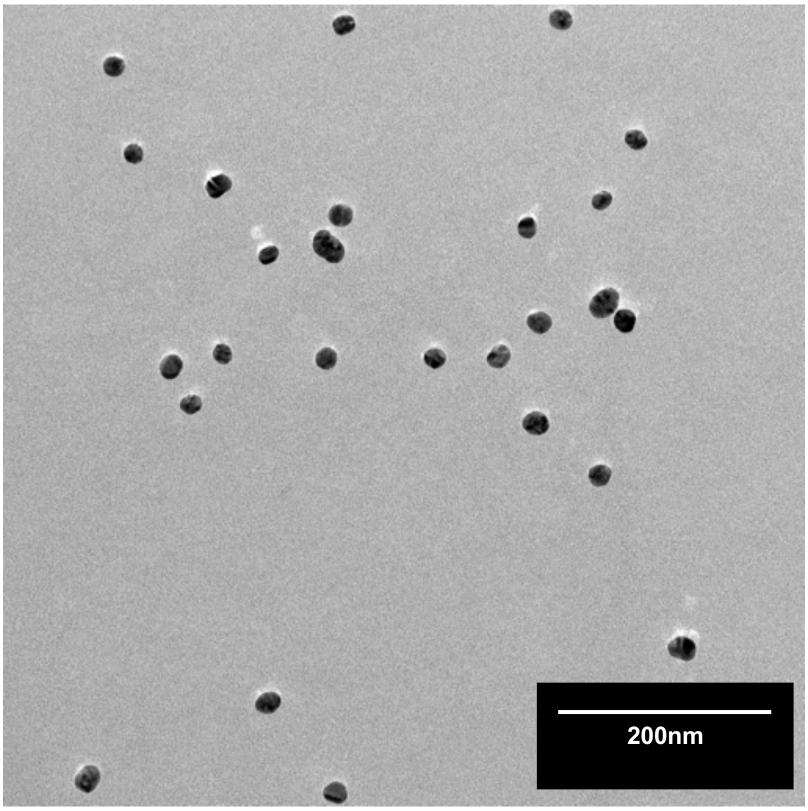

Figure S11. TEM micrograph of $AuNP_{17}$

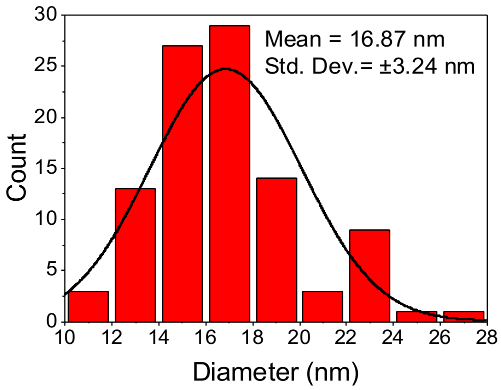

Figure S12. Histogram of TEM micrograph of $AuNP_{17}$

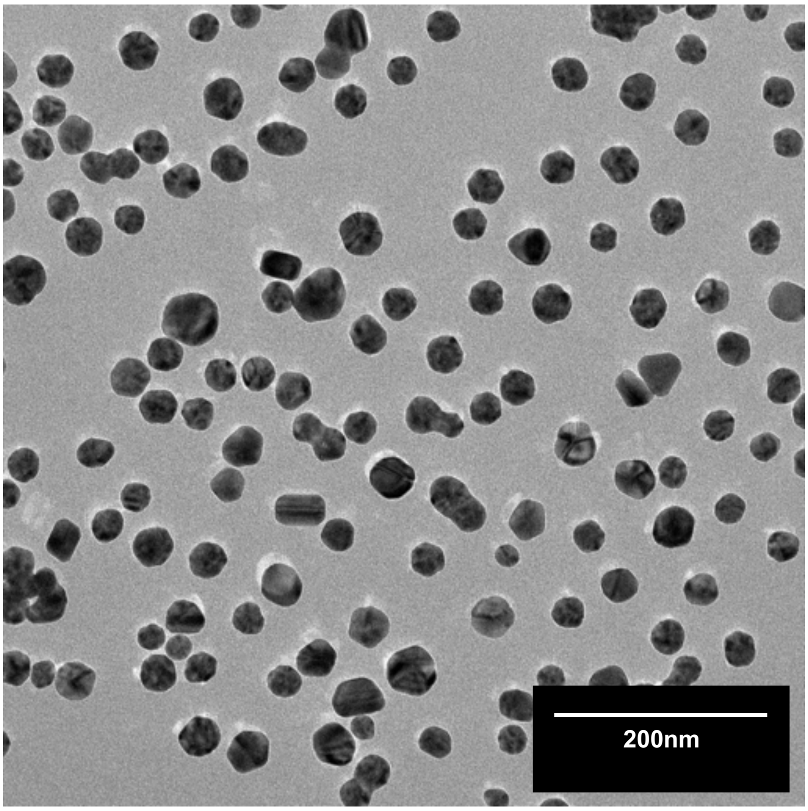

Figure S13. TEM micrograph of $AuNP_{30}$

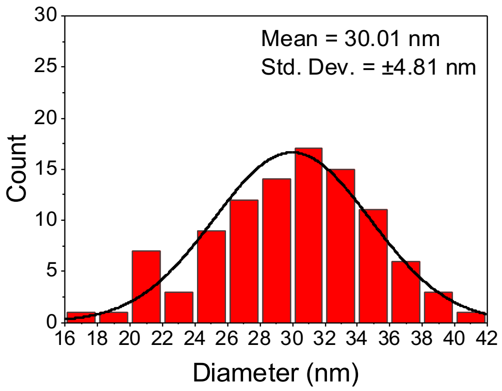

Figure S14. Histogram of TEM micrograph of $AuNP_{30}$

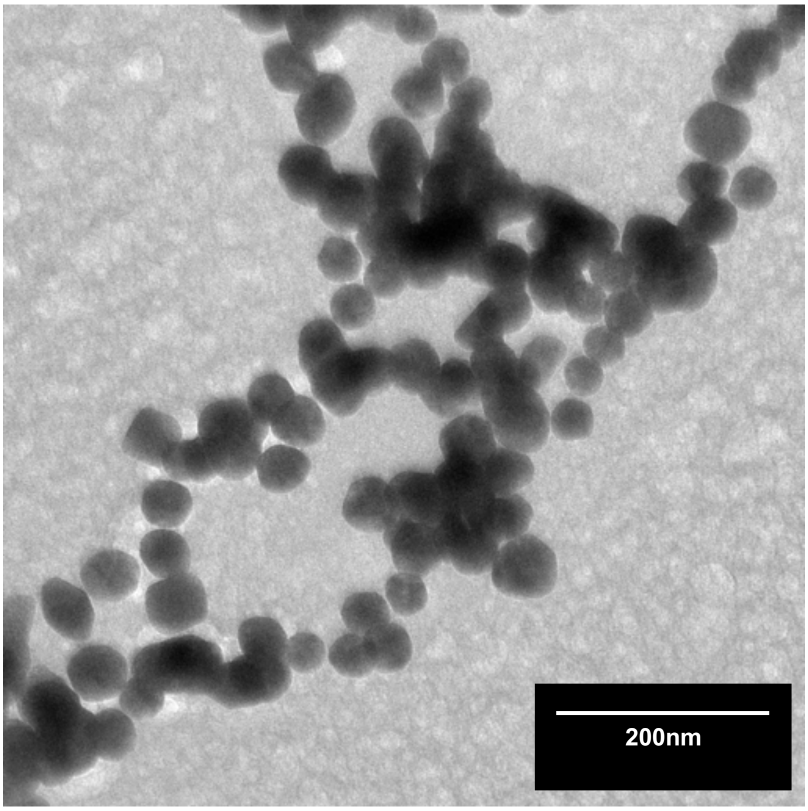

Figure S15. TEM micrograph of $AuNP_{39}$

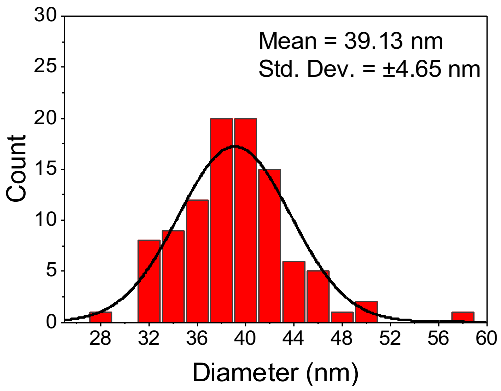

Figure S16. Histogram of TEM micrograph of $AuNP_{39}$

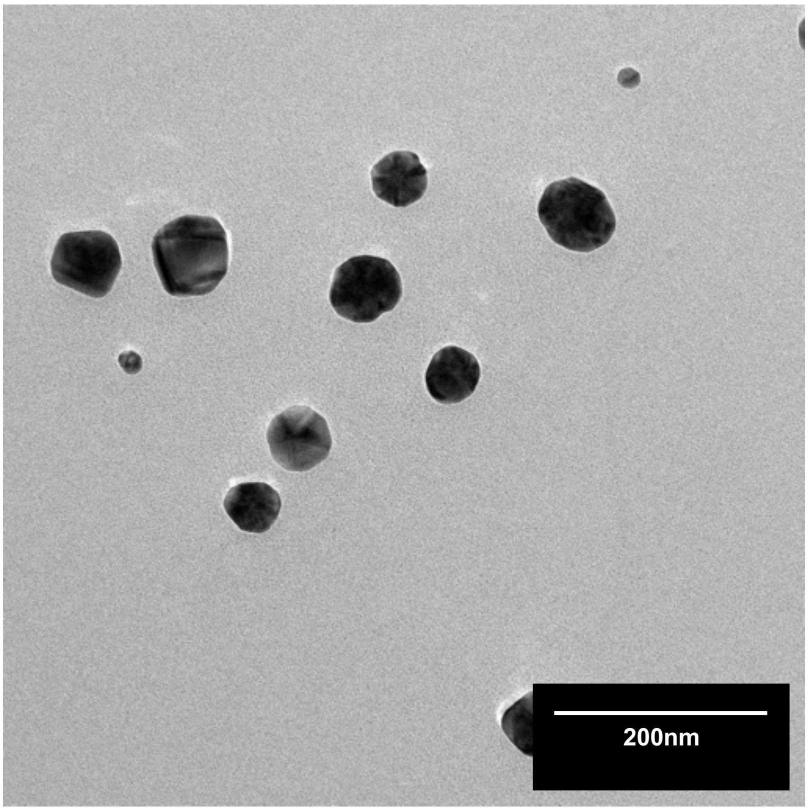

Figure S17. TEM micrograph of $AuNP_{56}$

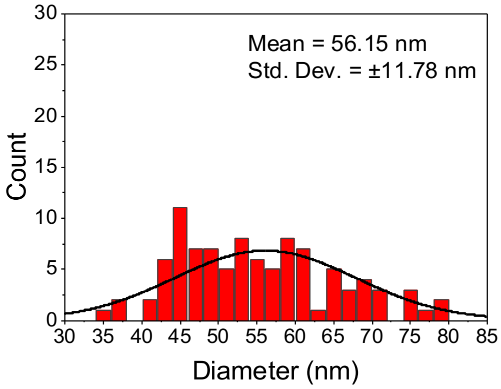

Figure S18. Histogram of TEM micrograph of $AuNP_{56}$

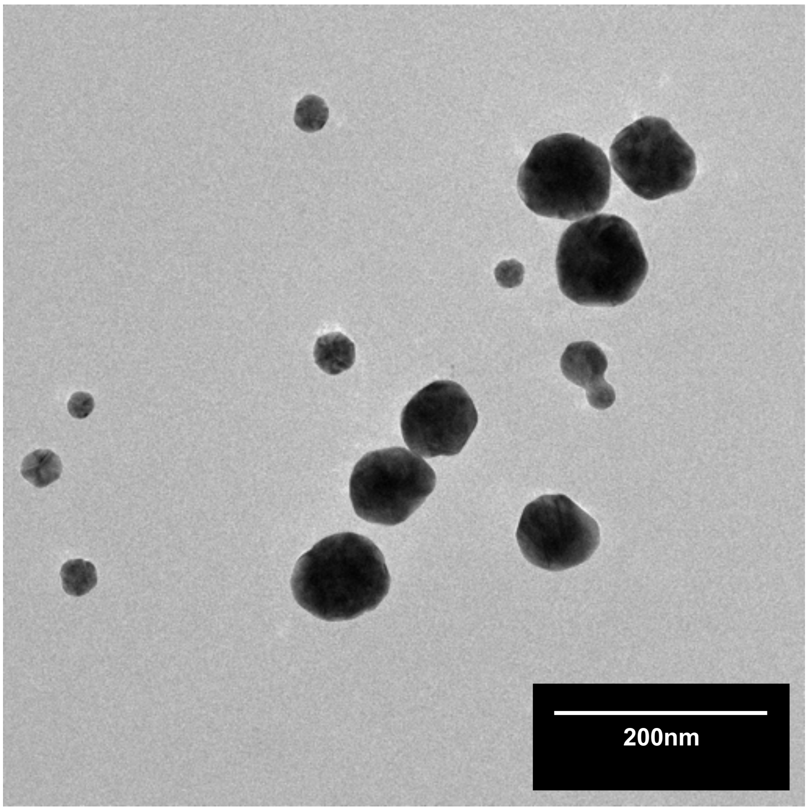

Figure S19. TEM micrograph of $AuNP_{72}$

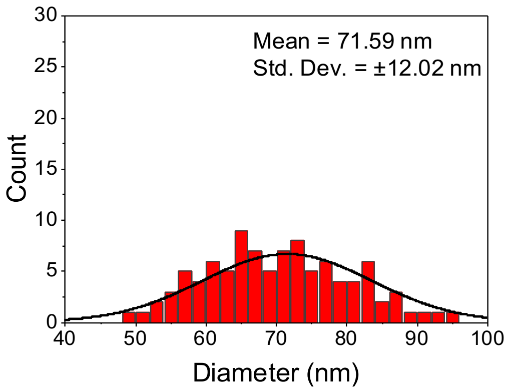

Figure S20. Histogram of TEM micrograph of $AuNP_{72}$

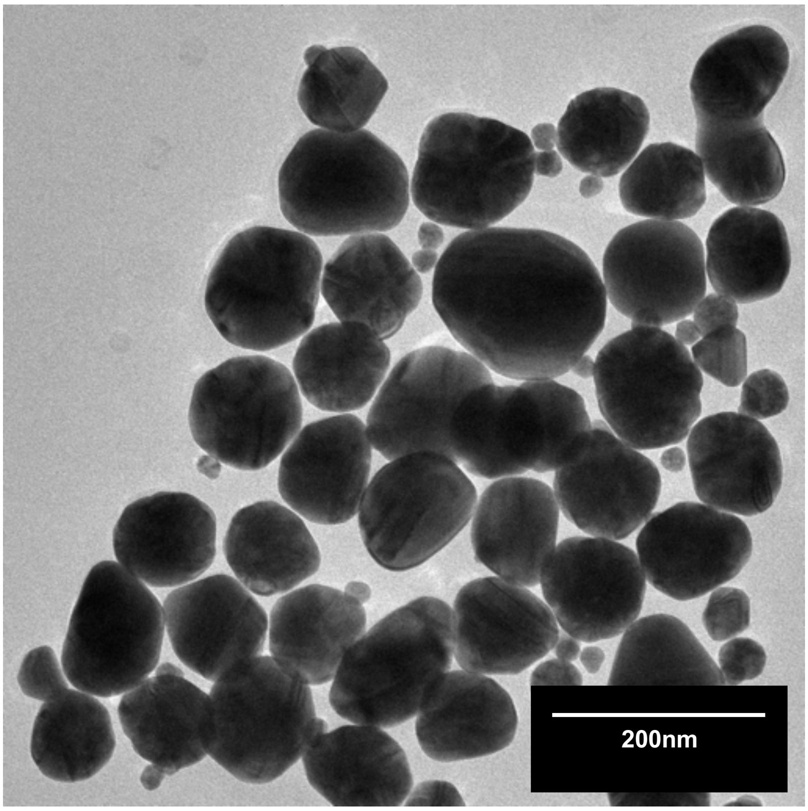

Figure S21. TEM micrograph of $AuNP_{83}$

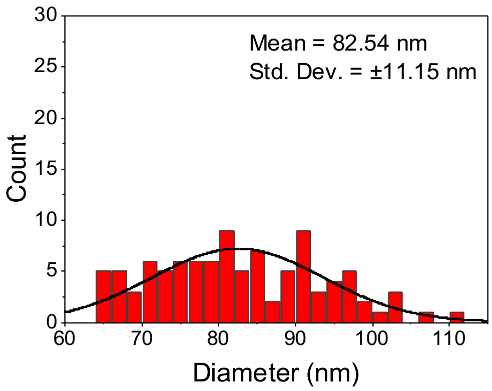

Figure S22. Histogram of TEM micrograph of $AuNP_{83}$

Analyzed XPS (x-ray Photoelectron Spectroscopy) data

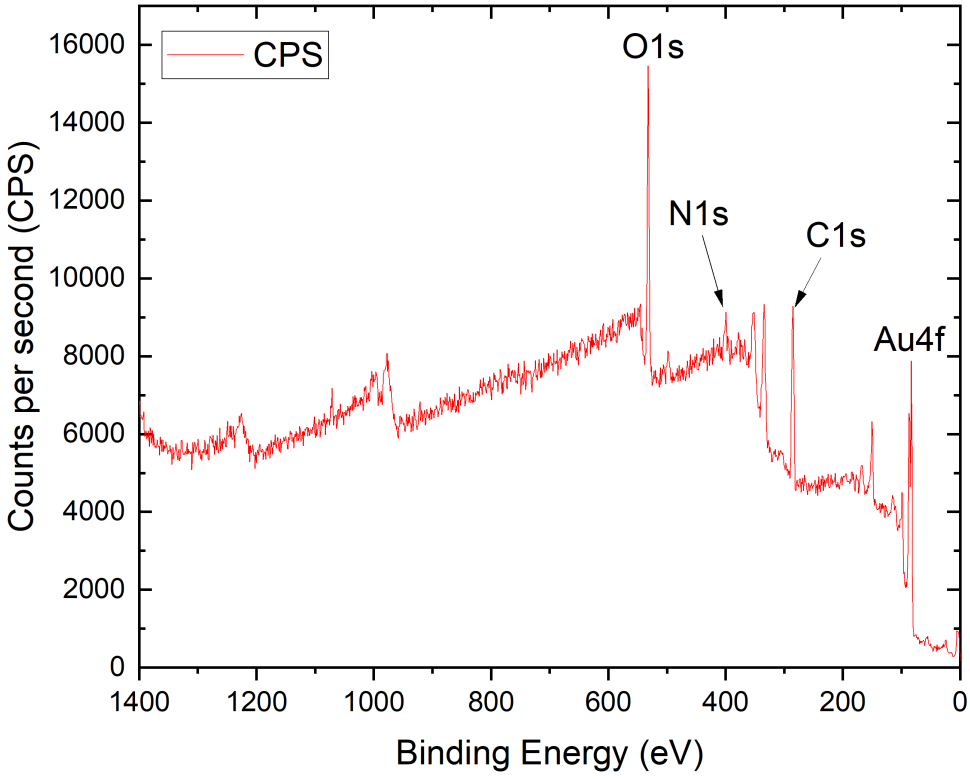

Figure S23. Representative XPS survey scan of glycopolymer functionalised AuNP (Gal-PHEA$_{33}$@AuNP$_{39}$)

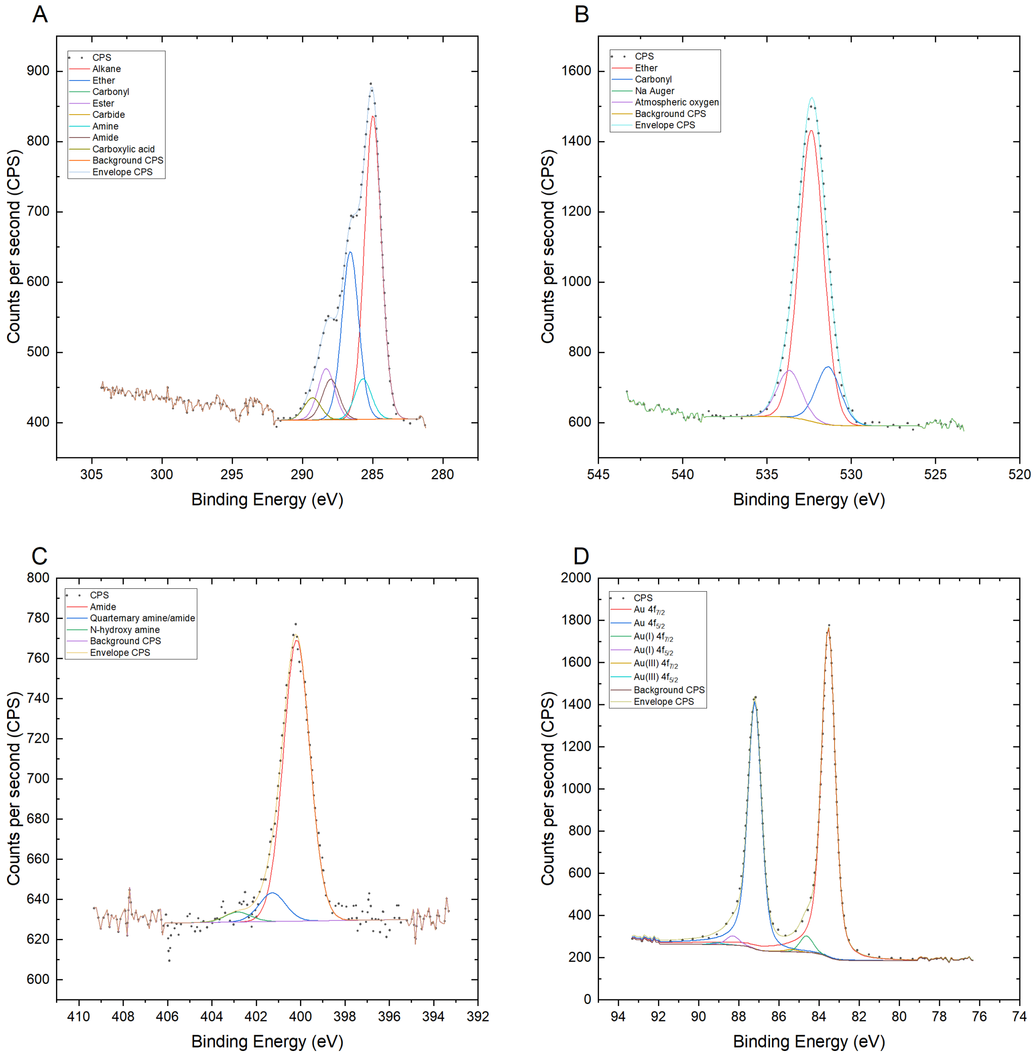

Figure S24. XPS of Gal-PHEA$_{33}$@AuNP$_{39}$ A) C 1*s* B) O 1*s* C) N 1*s* and D) Au 4*f*

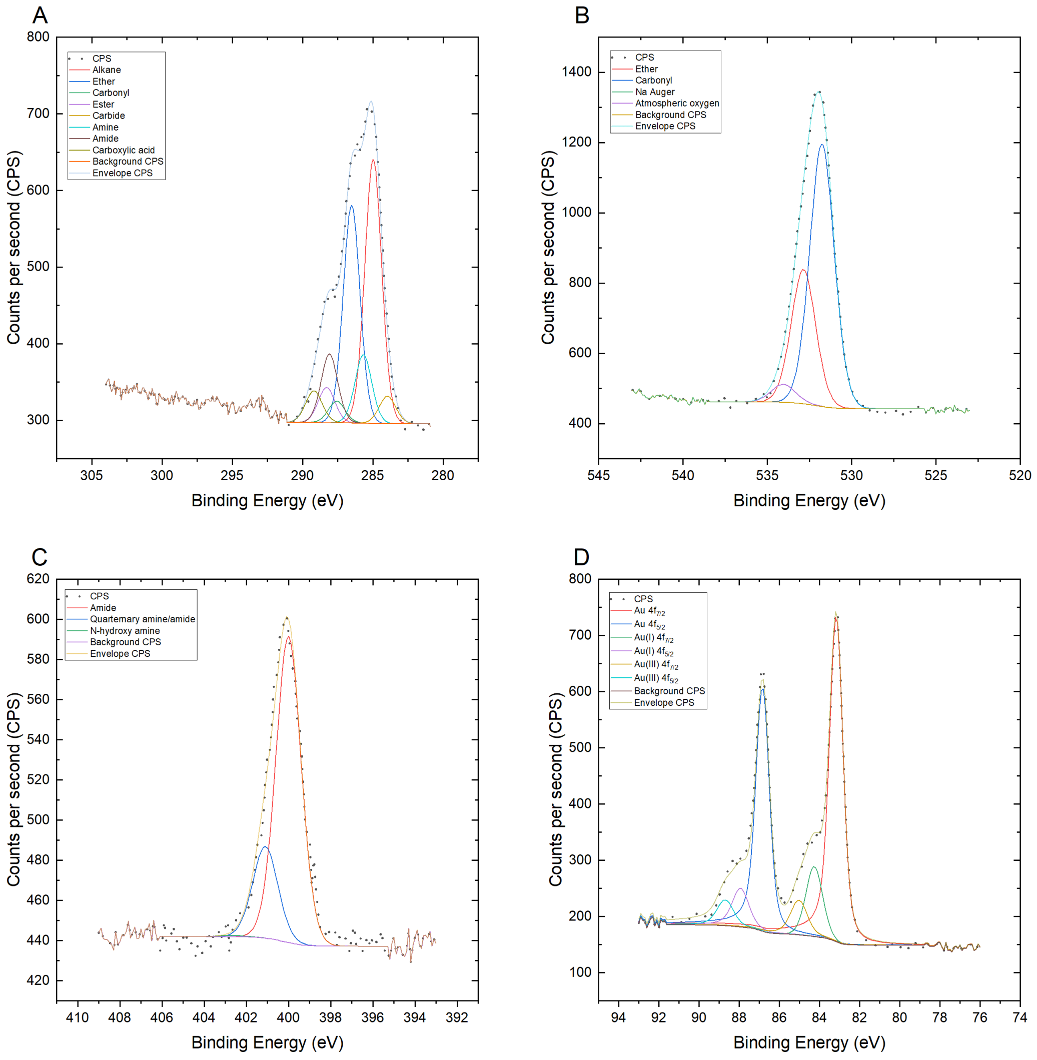

Figure S25. XPS of Gal-PHEA$_{33}$@AuNP$_{56}$ A) C 1*s* B) O 1*s* C) N 1*s* and D) Au 4*f*

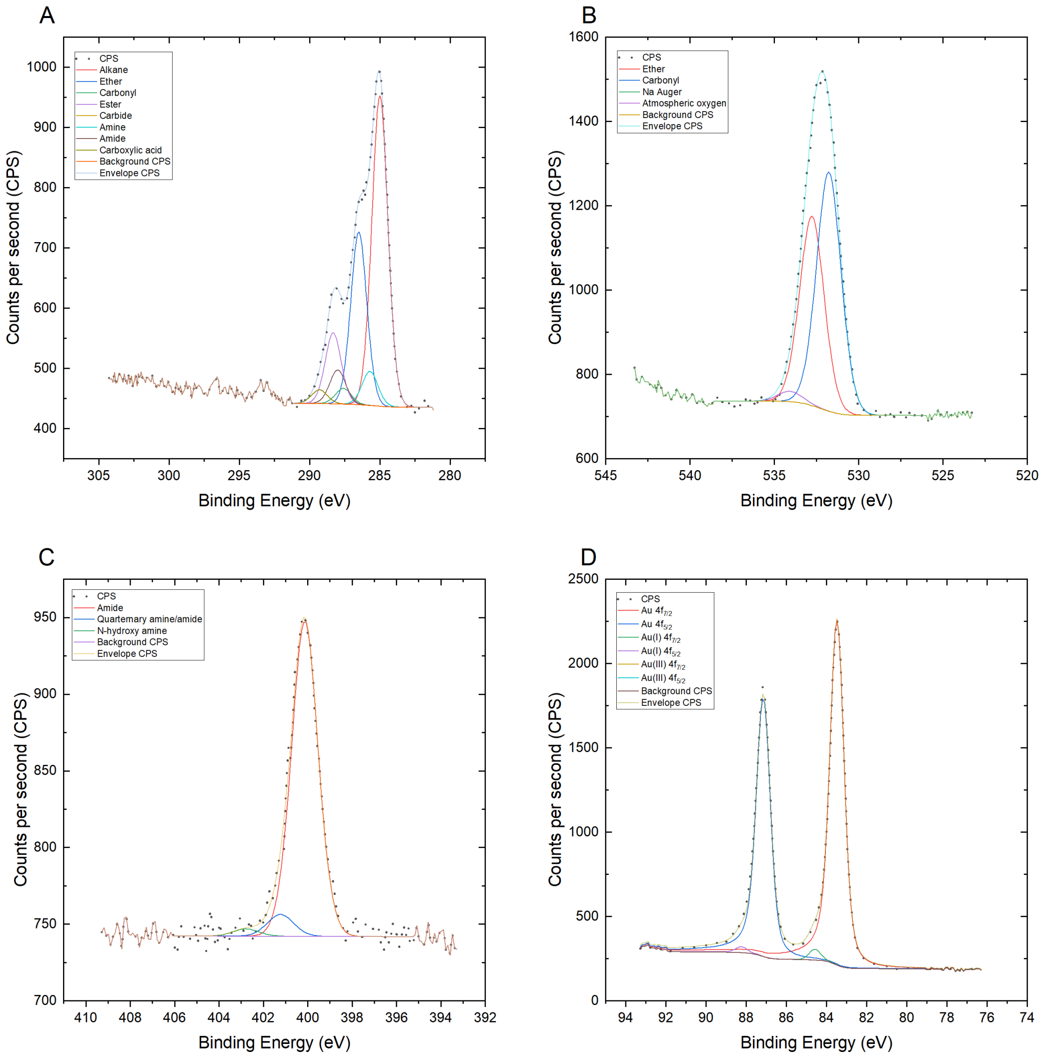

Figure S26. XPS of Gal-PHEA$_{40}$@AuNP$_{39}$ A) C 1*s* B) O 1*s* C) N 1*s* and D) Au 4*f*

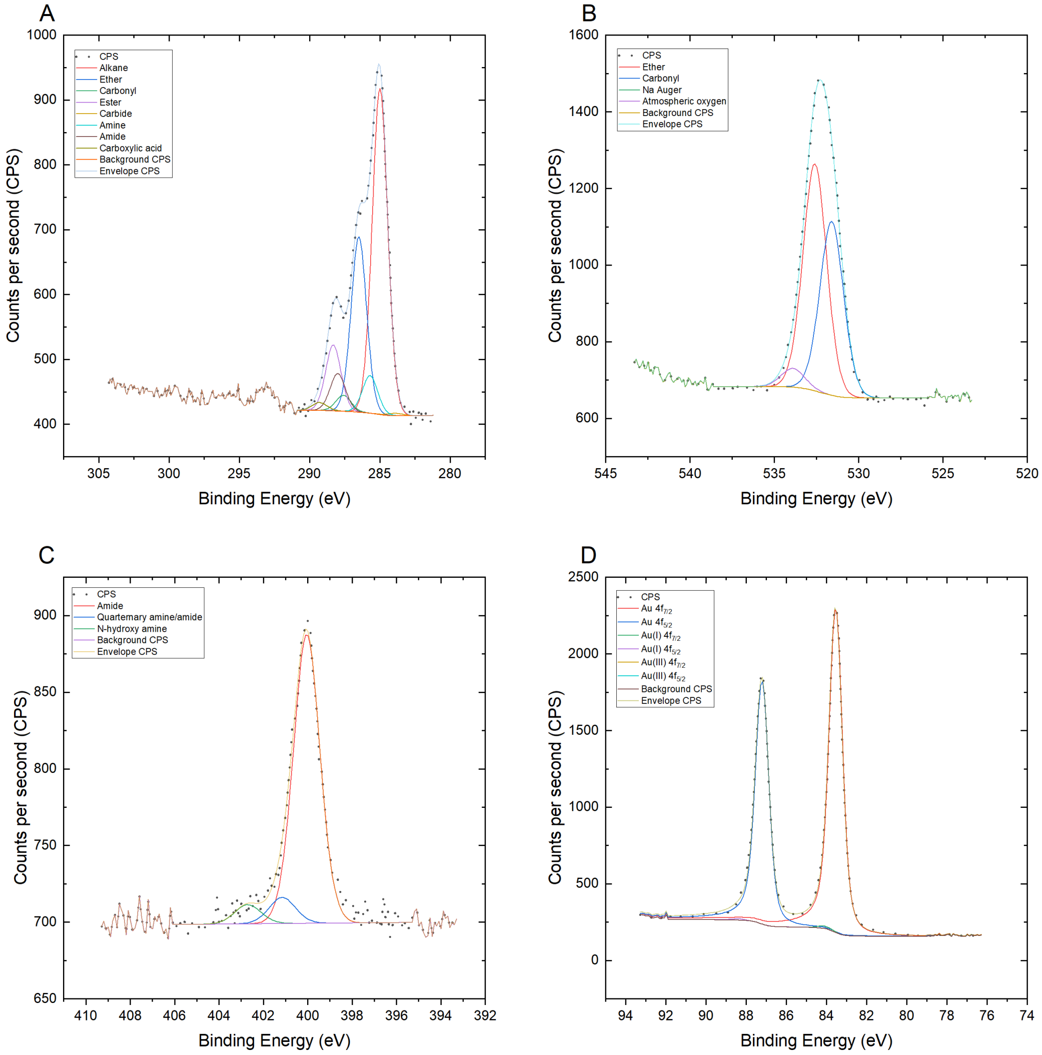

Figure S27. XPS of Gal-PHEA$_{40}$@AuNP$_{56}$ A) C 1*s* B) O 1*s* C) N 1*s* and D) Au 4*f*

Particle	Elemental Percentage Composition (%)				Elemental Ratios	
	C 1*s*	O 1*s*	N 1*s*	Au 4*f*	N 1*s*/C 1*s*	N 1*s*/Au 4*f*
Gal-pHEA$_{33}$@AuNP$_{39}$	57.60	28.82	5.80	7.78	0.14	0.75
Gal-pHEA$_{40}$@AuNP$_{39}$	59.60	23.86	7.43	9.11	0.15	0.82
Gal-pHEA$_{33}$@AuNP$_{56}$	60.23	29.17	7.08	3.52	0.06	2.01
Gal-pHEA$_{40}$@AuNP$_{56}$	57.65	25.18	7.70	9.46	0.16	0.81

Table S1. Elemental composition of nanoparticles determined by XPS

Particle	C 1*s* Bond Percentage Composition (%)								Bond Ratios	
	Alkane	Ether	Carbonyl	Ester	Carbide	Amine	Amide	Carboxylic acid	Amide/ Alkane	Amide/ Ether
Gal-pHEA$_{33}$@AuNP$_{39}$	48.31	26.78	0.16	8.19	0.00	6.49	6.49	3.58	0.13	0.24
Gal-pHEA$_{40}$@AuNP$_{39}$	47.57	26.50	2.43	10.90	0.00	5.24	5.24	2.12	0.11	0.20
Gal-pHEA$_{33}$@AuNP$_{56}$	35.91	29.60	2.95	4.80	3.74	9.34	9.34	4.33	0.26	0.32
Gal-pHEA$_{40}$@AuNP$_{56}$	48.71	26.21	2.40	9.86	0.37	5.62	5.62	1.20	0.12	0.21

Table S2. C 1*s* bonding composition of nanoparticle determined by XPS

Aggregation Assay Studies with SBA and WGA

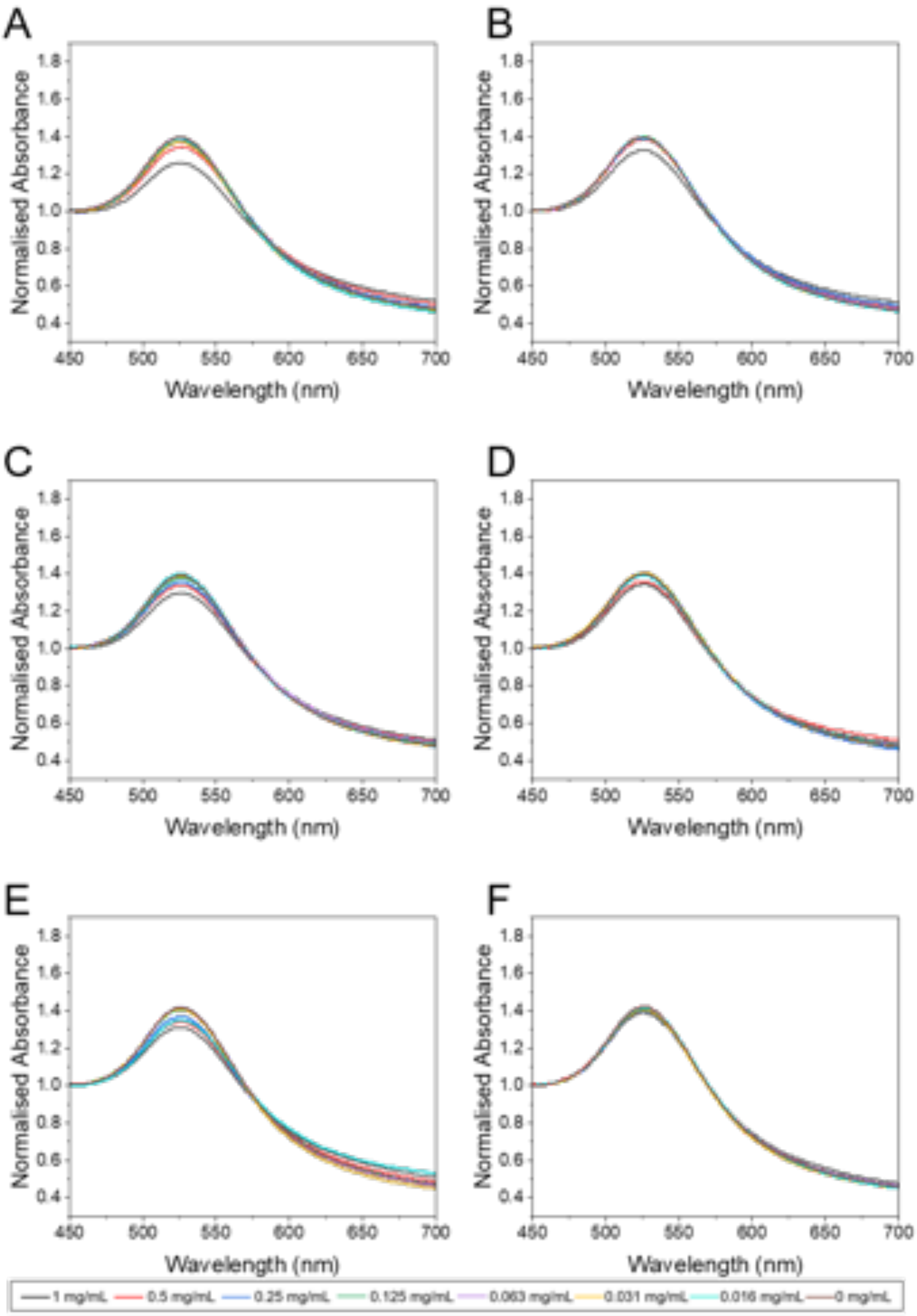

Figure S28. BLI data for 17 nm particles versus SBA (Left) & WGA (Right). A&B) Gal-PHEA$_{33}$@AuNP$_{17}$ C&D) Gal-PHEA$_{40}$@AuNP$_{17}$ and E&F) Gal-PHEA$_{74}$@AuNP$_{17}$

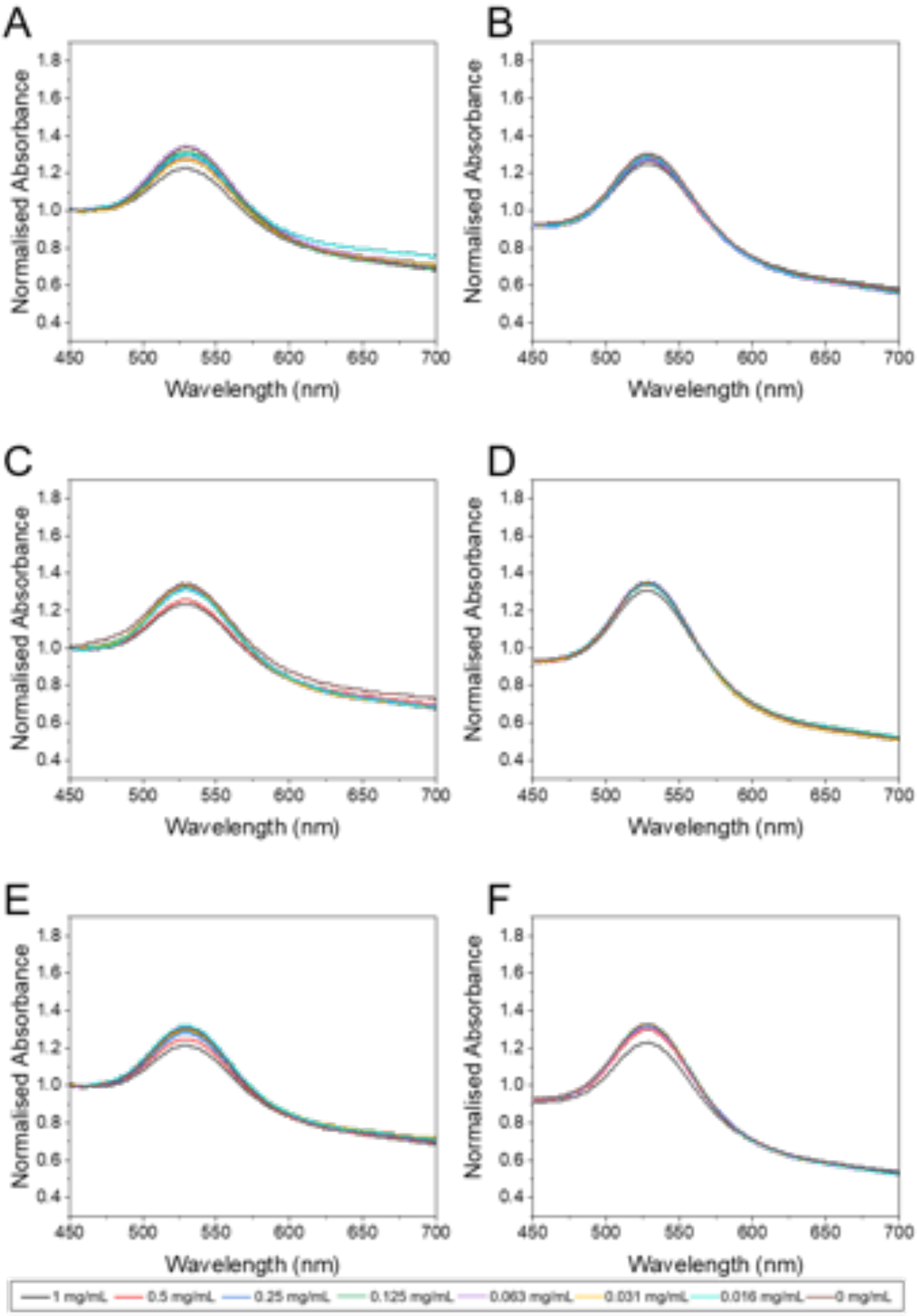

Figure S29. BLI data for 30 nm particles versus SBA (Left) & WGA (Right). A&B) Gal-PHEA$_{33}$@AuNP$_{30}$ C&D) Gal-PHEA$_{40}$@AuNP$_{30}$ and E&F) Gal-PHEA$_{74}$@AuNP$_{30}$

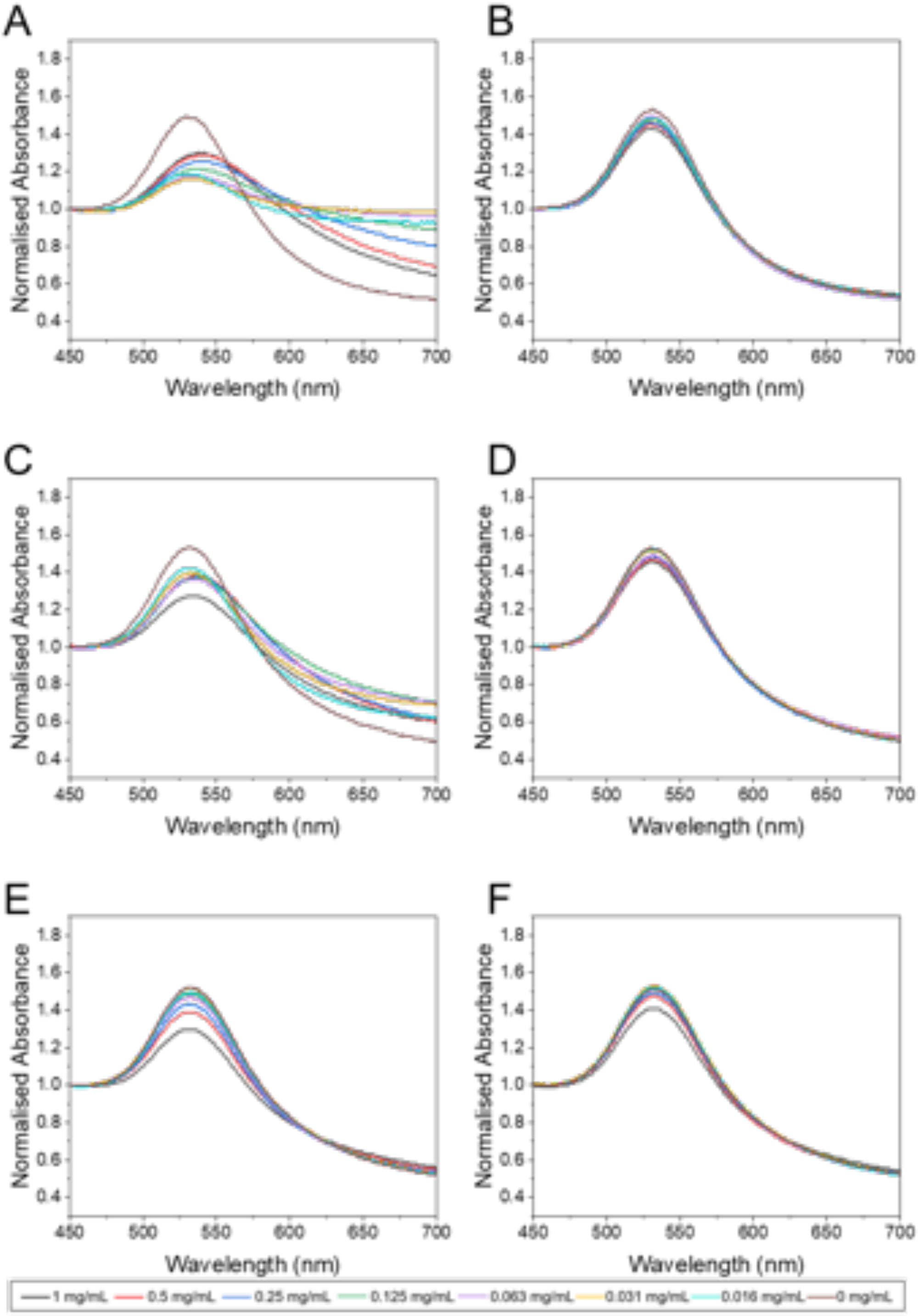

Figure S30. BLI data for 39 nm particles versus SBA (Left) & WGA (Right). A&B) Gal-PHEA$_{33}$@AuNP$_{39}$ C&D) Gal-PHEA$_{40}$@AuNP$_{39}$ and E&F) Gal-PHEA$_{74}$@AuNP$_{39}$

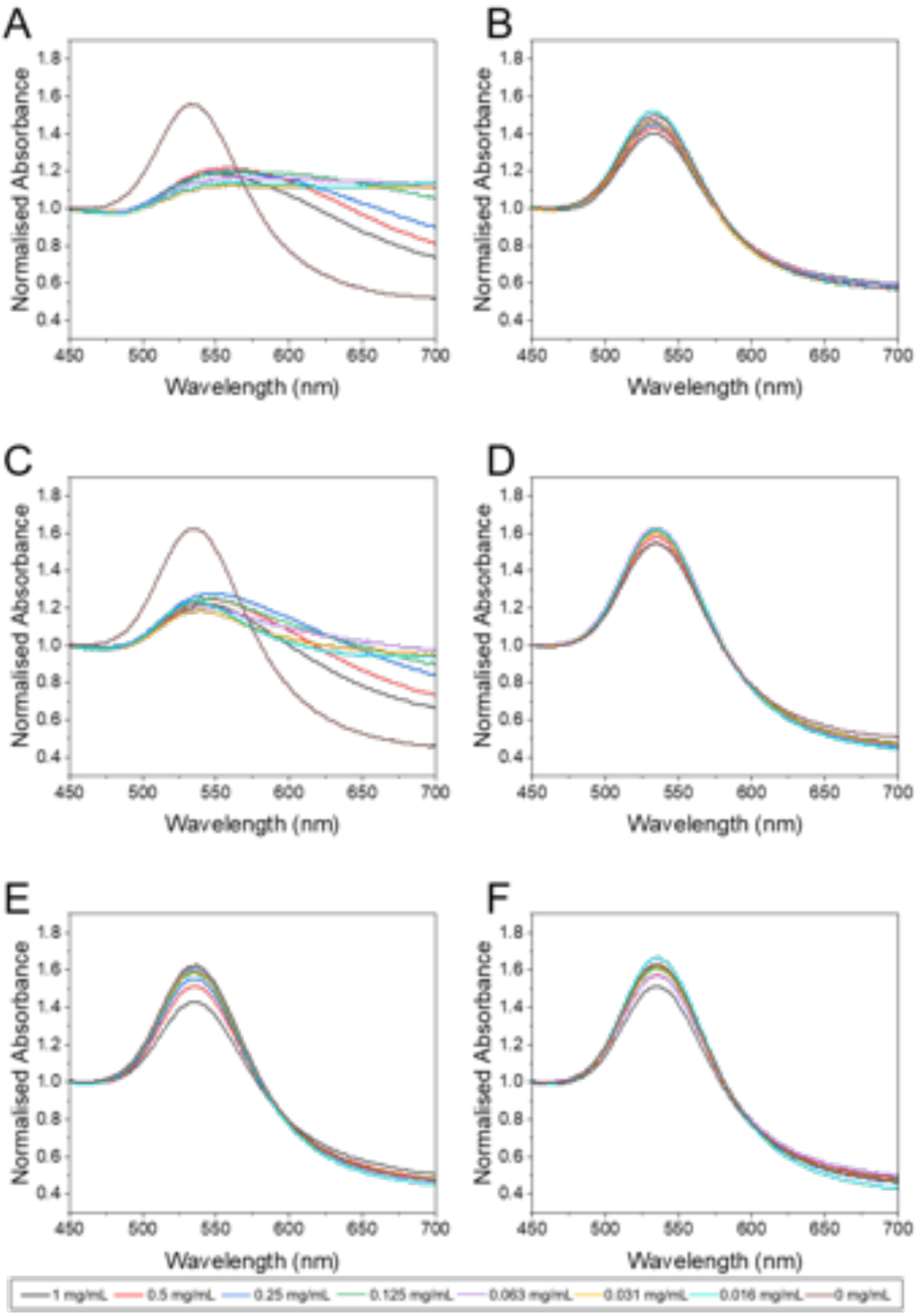

Figure S31. BLI data for 56 nm particles versus SBA (Left) & WGA (Right). A&B) Gal-PHEA$_{33}$@AuNP$_{56}$ C&D) Gal-PHEA$_{40}$@AuNP$_{56}$ and E&F) Gal-PHEA$_{74}$@AuNP$_{56}$

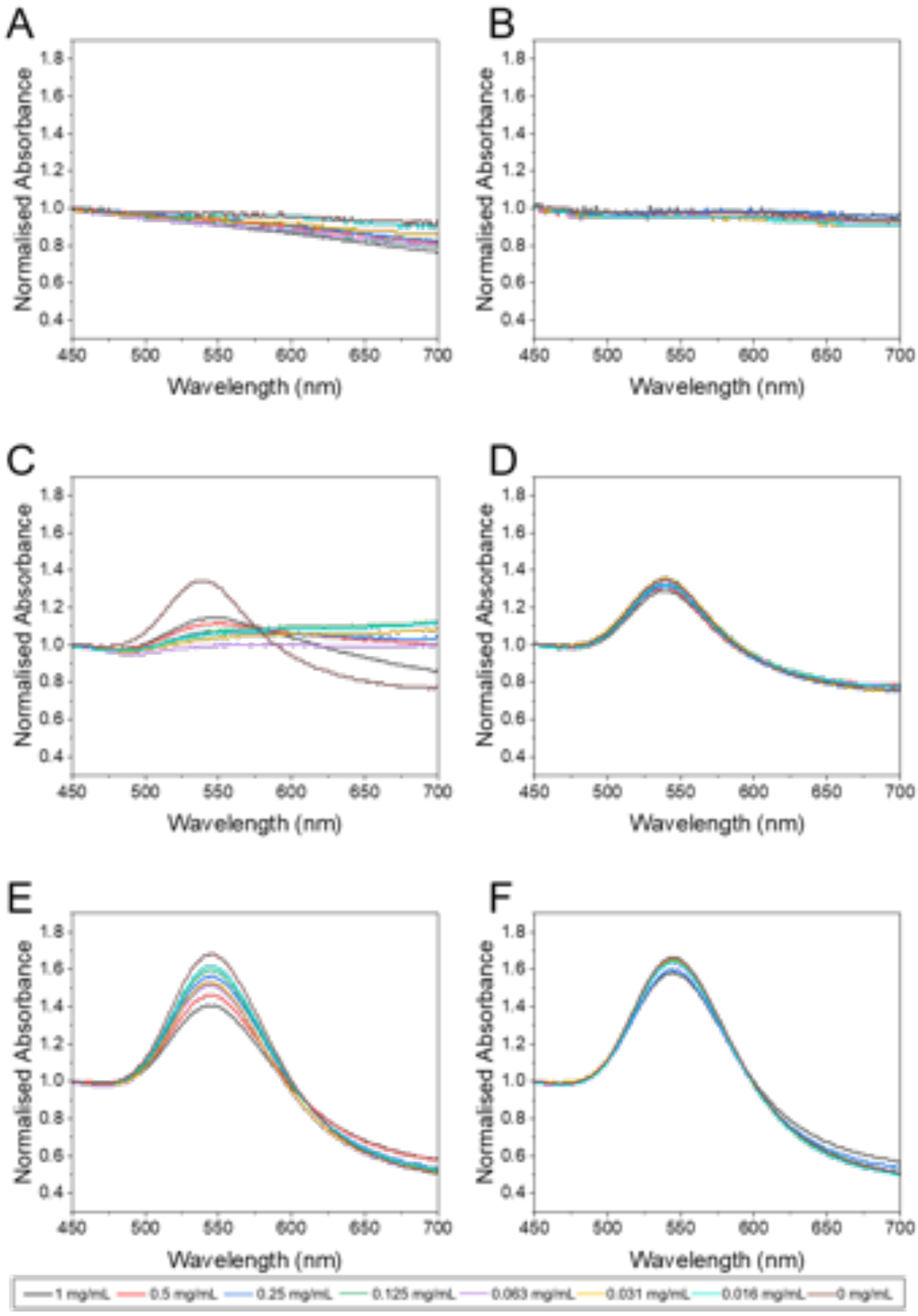

Figure S32. BLI data for 72 nm particles versus SBA (Left) & WGA (Right). A&B) Gal-PHEA$_{33}$@AuNP$_{72}$ C&D) Gal-PHEA$_{40}$@AuNP$_{72}$ and E&F) Gal-PHEA$_{74}$@AuNP$_{72}$

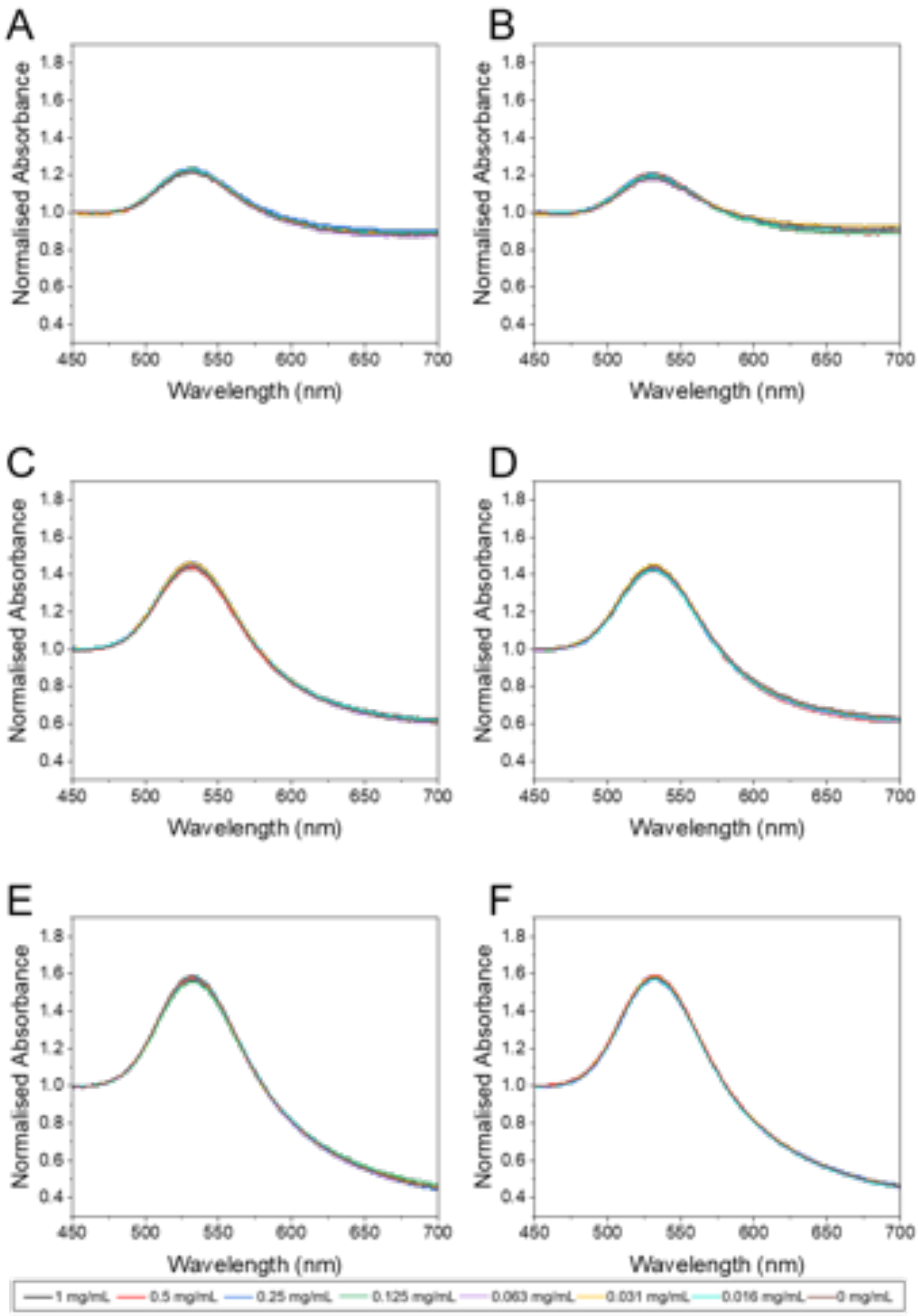

Figure S33. BLI data for 83 nm particles versus SBA (Left) & WGA (Right). A&B) Gal-PHEA$_{33}$@AuNP$_{83}$ C&D) Gal-PHEA$_{40}$@AuNP$_{83}$ and E&F) Gal-PHEA$_{74}$@AuNP$_{83}$

Aggregation Assay Studies of SBA, WGA and HPA at low concentrations

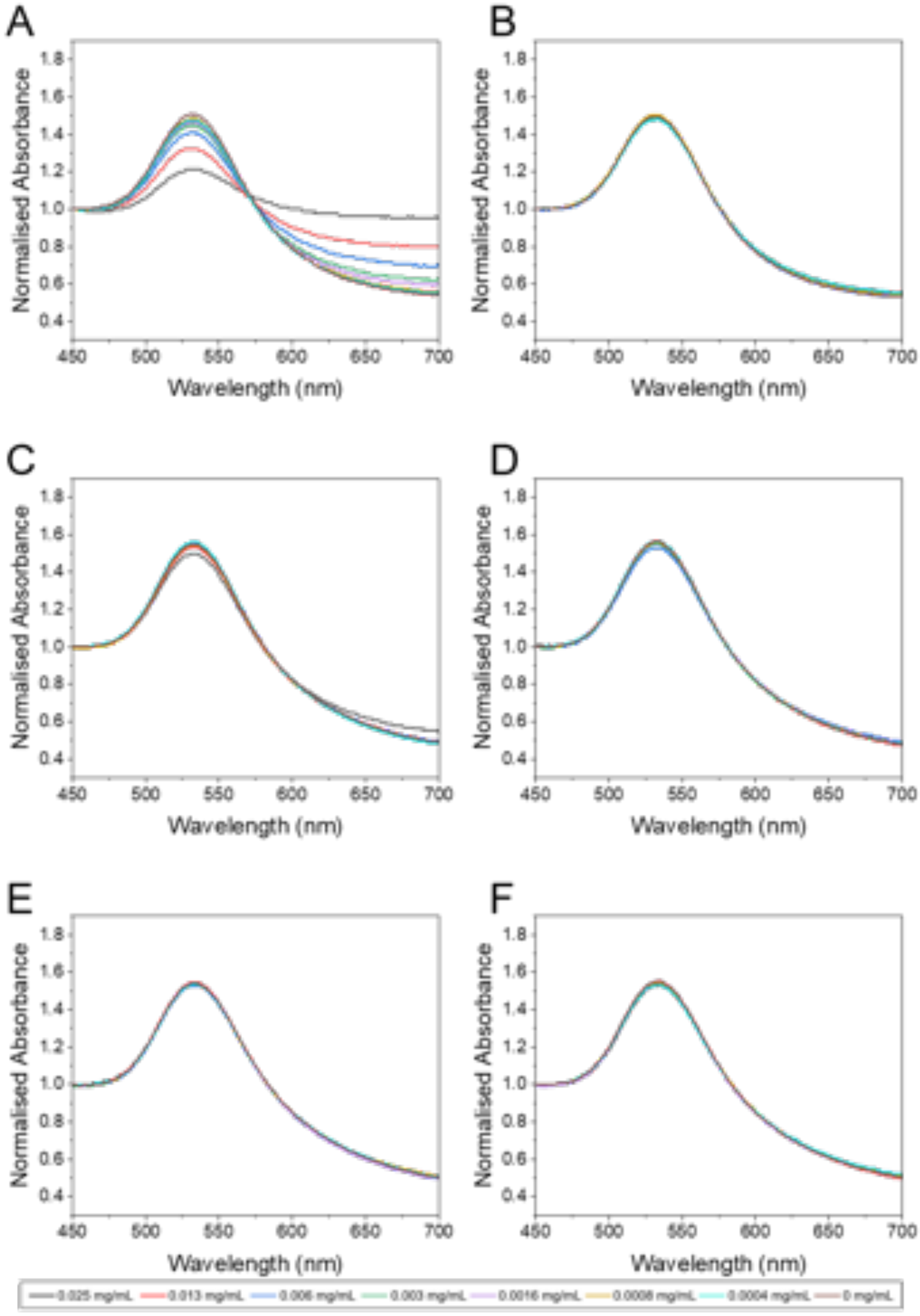

Figure S34. Plot of normalised absorbance of 39 nm AuNPs of varying polymer length in varying concentrations of SBA (Left) and WGA (Right). A&B) Gal-PHEA$_{33}$@AuNP$_{39}$ C&D) Gal-PHEA$_{40}$@AuNP$_{39}$ and E&F) Gal-PHEA$_{74}$@AuNP$_{39}$

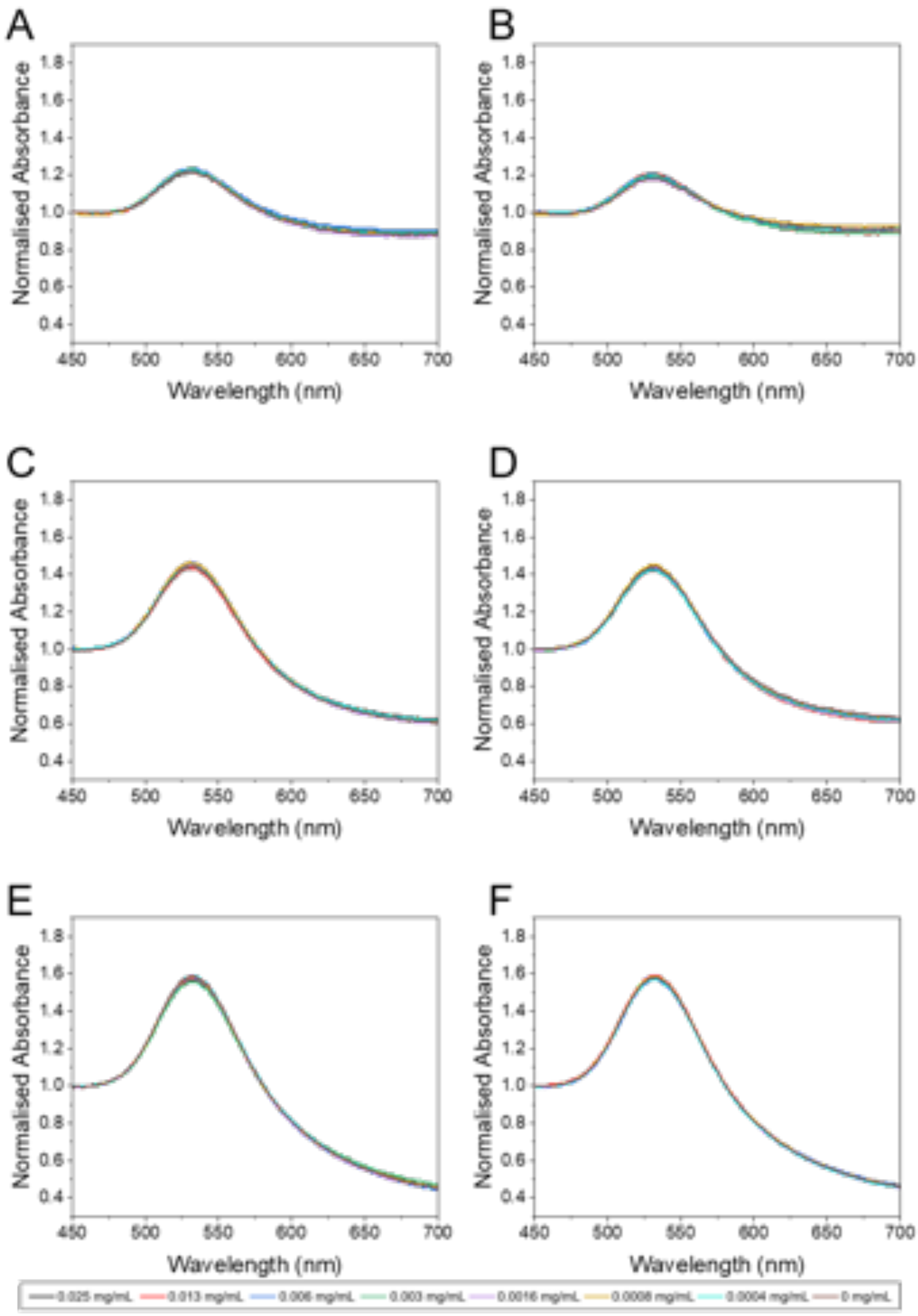

Figure S35. Plot of normalised absorbance of 39 nm AuNPs of varying polymer length in varying concentrations of HPA (Left) and WGA (Right). A&B) Gal-PHEA$_{33}$@AuNP$_{39}$ C&D) Gal-PHEA$_{40}$@AuNP$_{39}$ and E&F) Gal-PHEA$_{74}$@AuNP$_{39}$

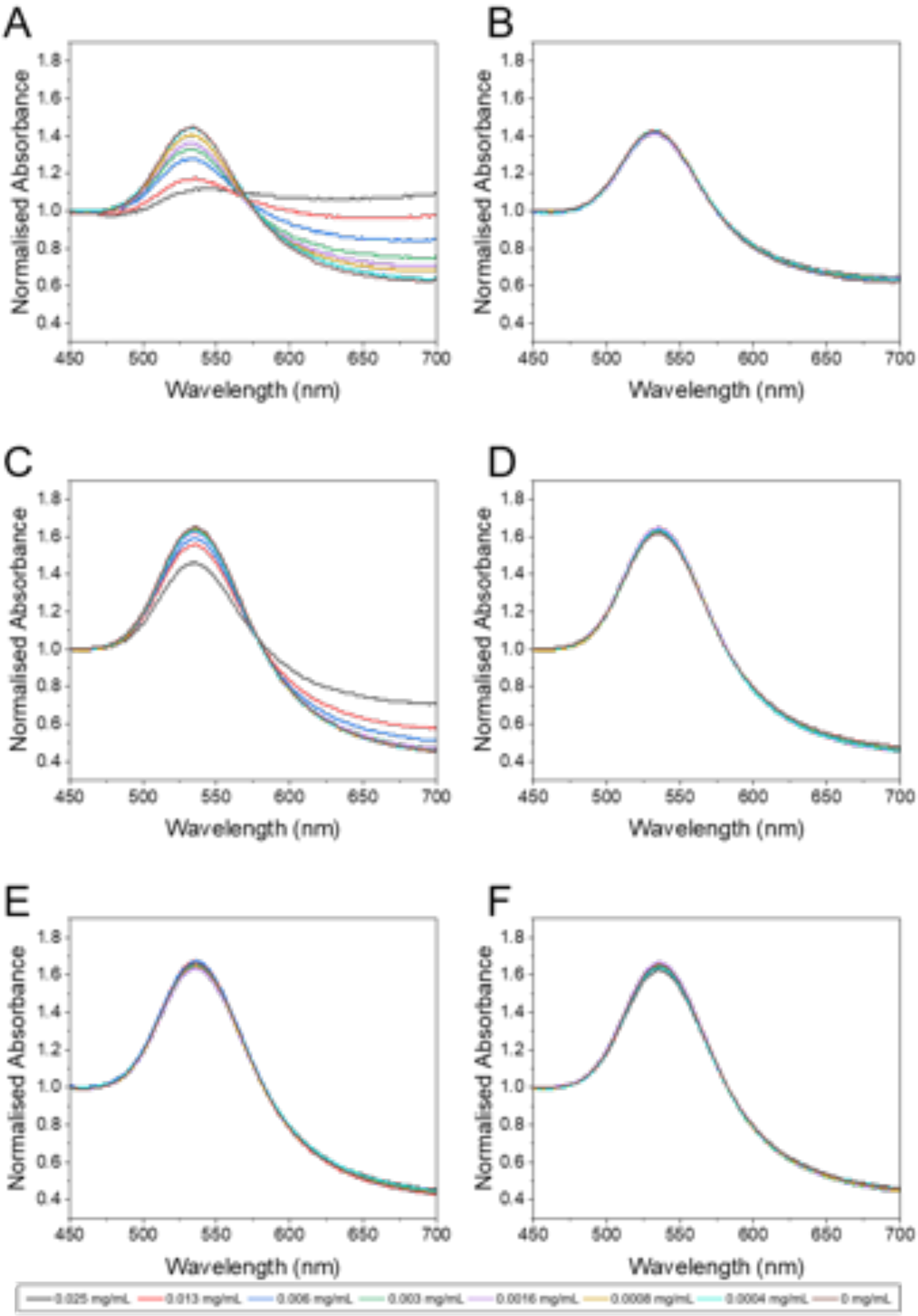

Figure S36. Plot of normalised absorbance of 56 nm AuNPs of varying polymer length in varying concentrations of SBA (Left) and WGA (Right). A&B) Gal-PHEA$_{33}$@AuNP$_{56}$ C&D) Gal-PHEA$_{40}$@AuNP$_{56}$ and E&F) Gal-PHEA$_{74}$@AuNP$_{56}$

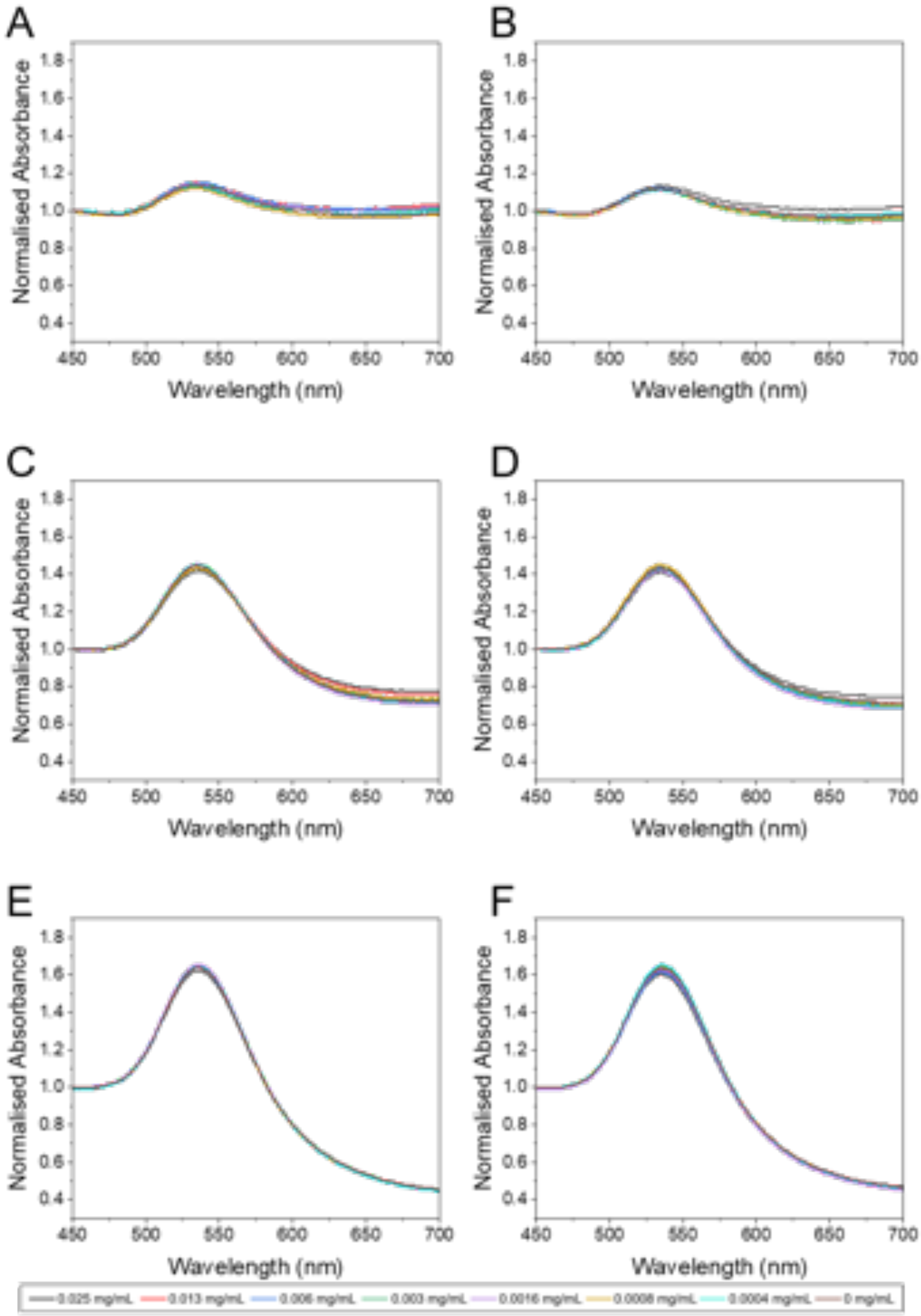

Figure S37. Plot of normalised absorbance of 56 nm AuNPs of varying polymer length in varying concentrations of HPA (Left) and WGA (Right). A&B) Gal-PHEA$_{33}$@AuNP$_{56}$ C&D) Gal-PHEA$_{40}$@AuNP$_{56}$ and E&F) Gal-PHEA$_{74}$@AuNP$_{56}$

System	LoD (µg SBA/mL)	LoD (nM SBA)
Gal-pHEA$_{33}$@AuNP$_{39}$	1.56	14
Gal-pHEA$_{40}$@AuNP$_{39}$	25.0	224
Gal-pHEA$_{33}$@AuNP$_{56}$	0.78	7
Gal-pHEA$_{40}$@AuNP$_{56}$	6.25	56

Table S3. Estimate of limit detection of the AuNP-pHEA systems for SBA. This is not intended as a limit for e.g. diagnostics but to show how each formulation can give a different response and why the polymer linker length is crucial.

$$Normalised\ Abs_{700} = \left(\left(\frac{Normalised\ Abs_{700}\ SBA_x}{Normalised\ Abs_{SPR}\ SBA_x}\right) - \left(\frac{Normalised\ Abs_{700}\ Buffer}{Normalised\ Abs_{SPR}\ Buffer}\right)\right) - \left(\left(\frac{Normalised\ Abs_{700}\ WGA_x}{Normalised\ Abs_{SPR}\ WGA_x}\right) - \left(\frac{Normalised\ Abs_{700}\ Buffer}{Normalised\ Abs_{SPR}\ Buffer}\right)\right)$$

Equation 1. Calculation of normalised Abs_{700} for each concentration [x] of SBA and WGA in Figure 3C.

Aggregation Assay Data for Varying OD

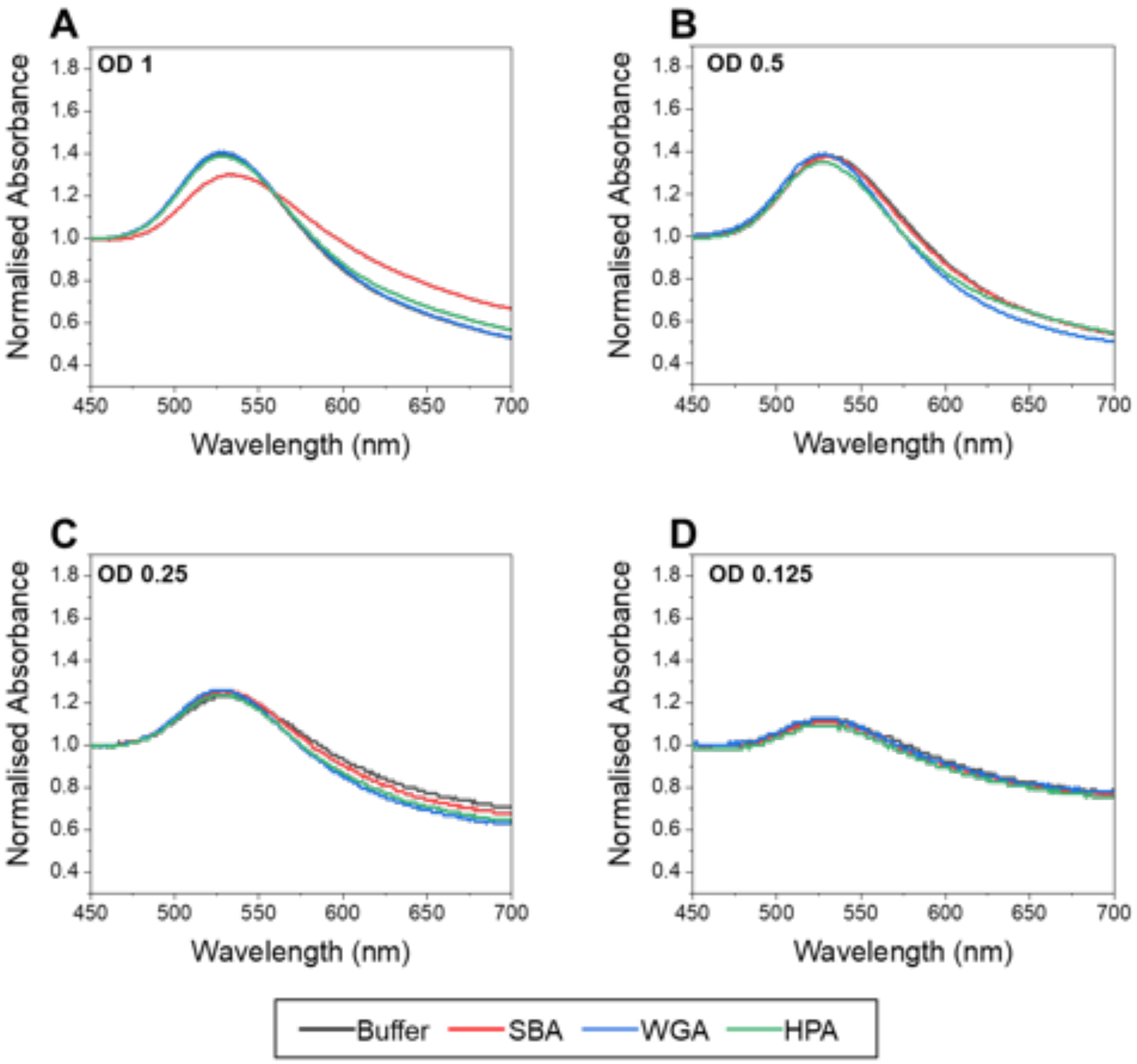

Figure S38. UV-Visible spectra of Gal-pHEA$_{33}$@AuNP$_{17}$ after addition of SBA, HPA or WGA. All lectins used at 0.1 mg.mL^{-1}. A) OD 1, B) OD 0.5, C) OD 0.25 and D) OD 0.125.

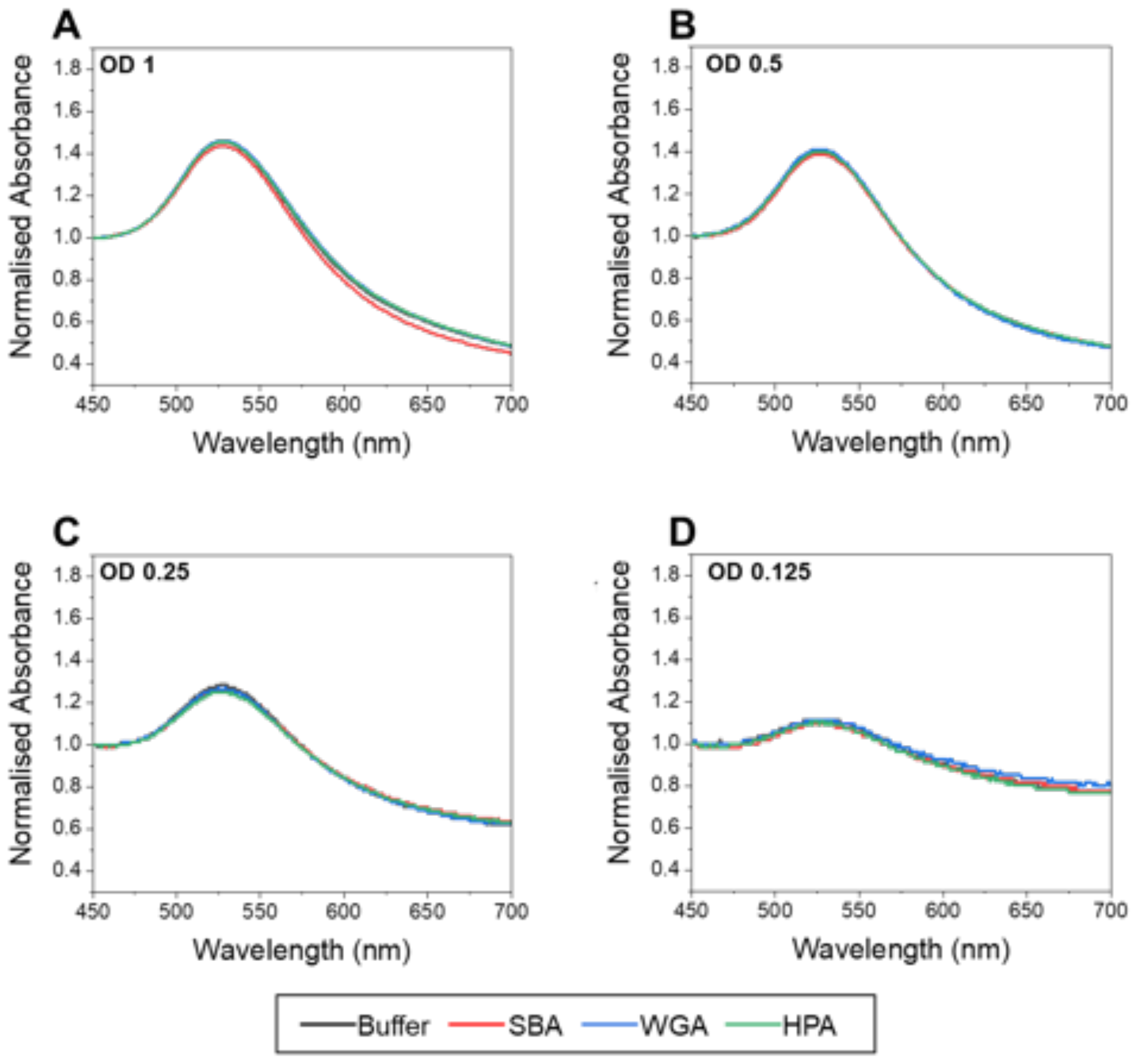

Figure S39. UV-Visible spectra of Gal-pHEA$_{40}$@AuNP$_{17}$ after addition of SBA, HPA or WGA. All lectins used at 0.1 mg.mL^{-1}. A) OD 1, B) OD 0.5, C) OD 0.25 and D) OD 0.125.

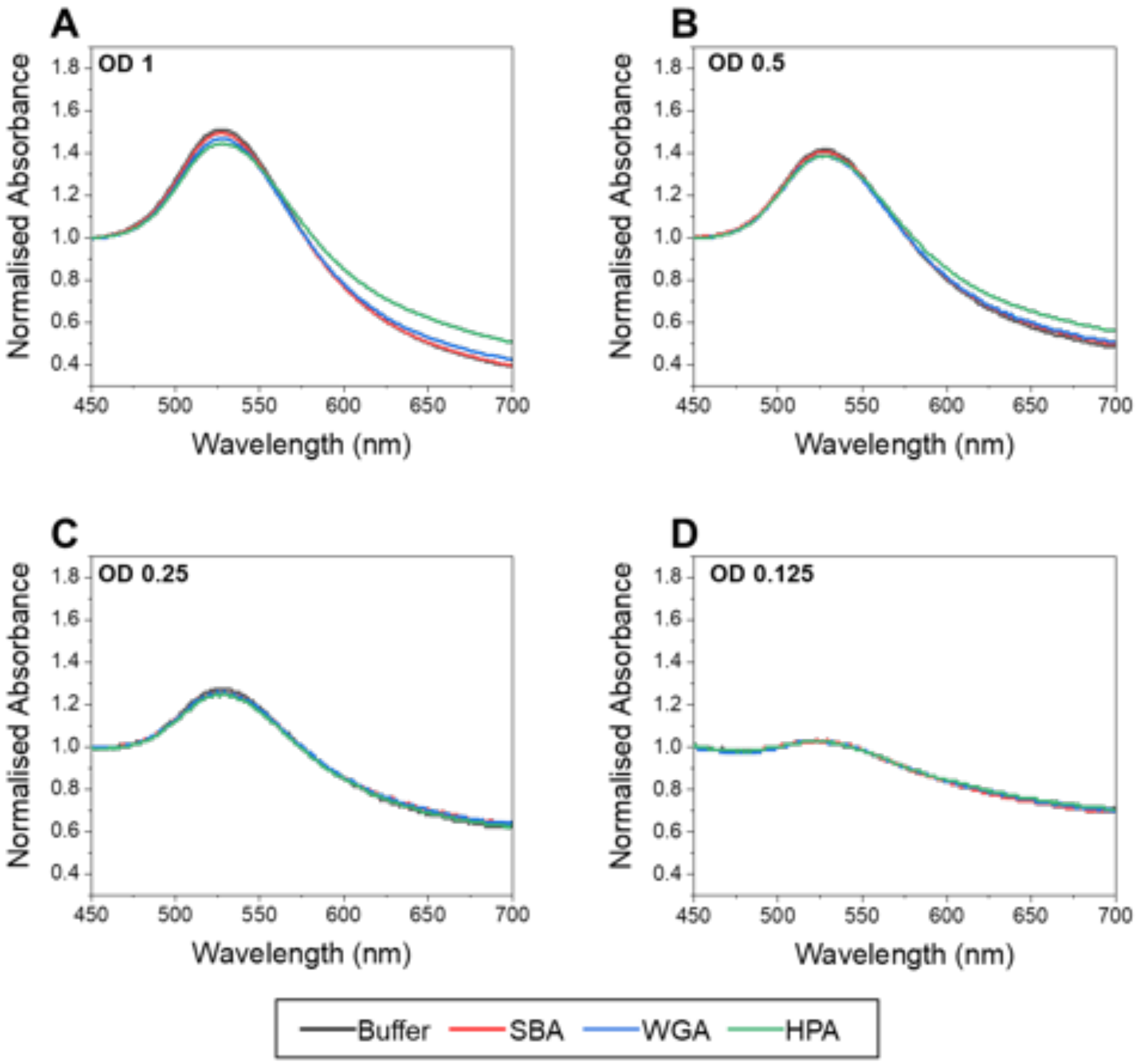

Figure S40. UV-Visible spectra of Gal-pHEA$_{74}$@AuNP$_{17}$ after addition of SBA, HPA or WGA. All lectins used at 0.1 mg.mL^{-1}. A) OD 1, B) OD 0.5, C) OD 0.25 and D) OD 0.125.

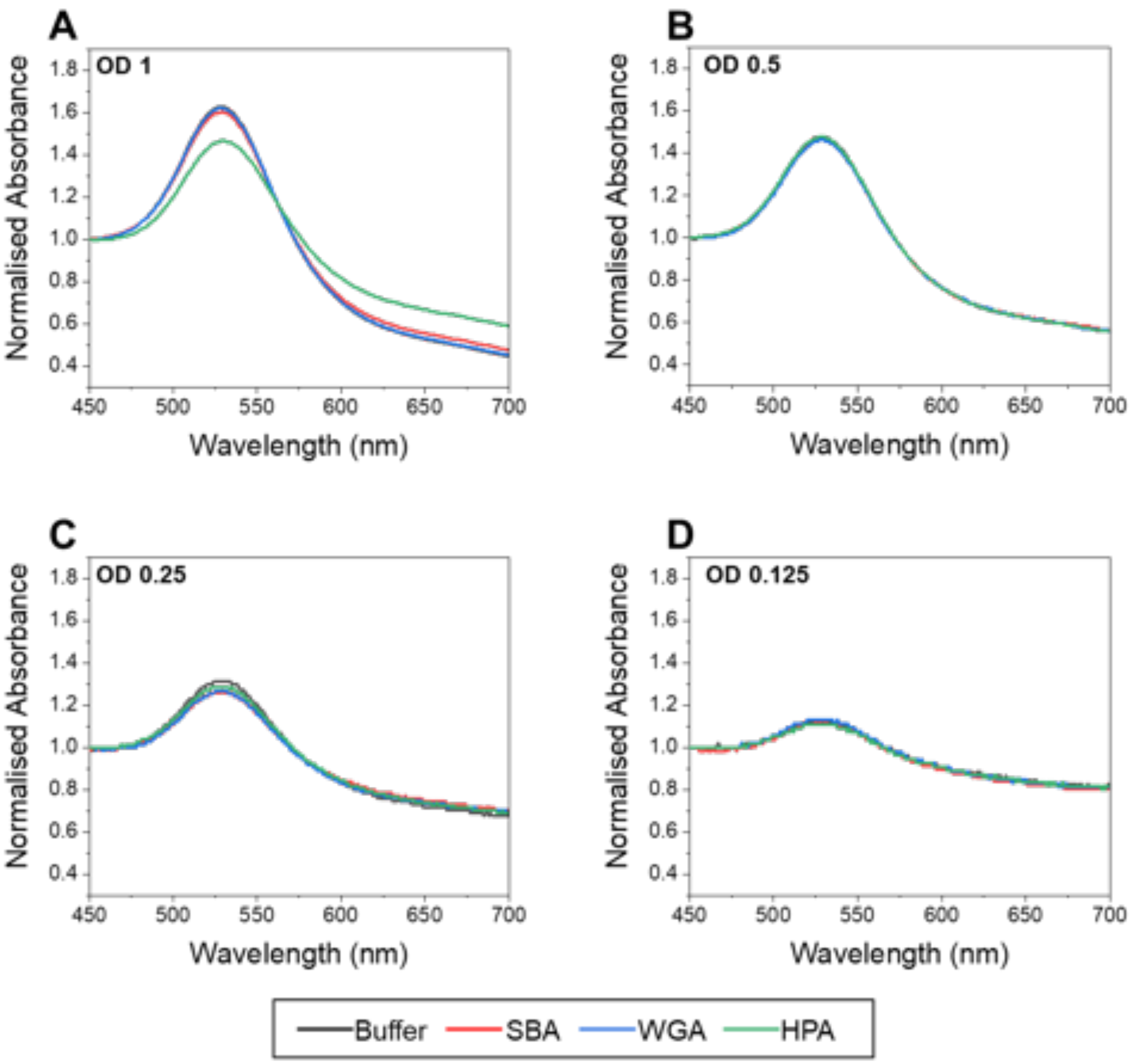

Figure S41. UV-Visible spectra of Gal-pHEA$_{33}$@AuNP$_{30}$ after addition of SBA, HPA or WGA. All lectins used at 0.1 mg.mL^{-1}. A) OD 1, B) OD 0.5, C) OD 0.25 and D) OD 0.125.

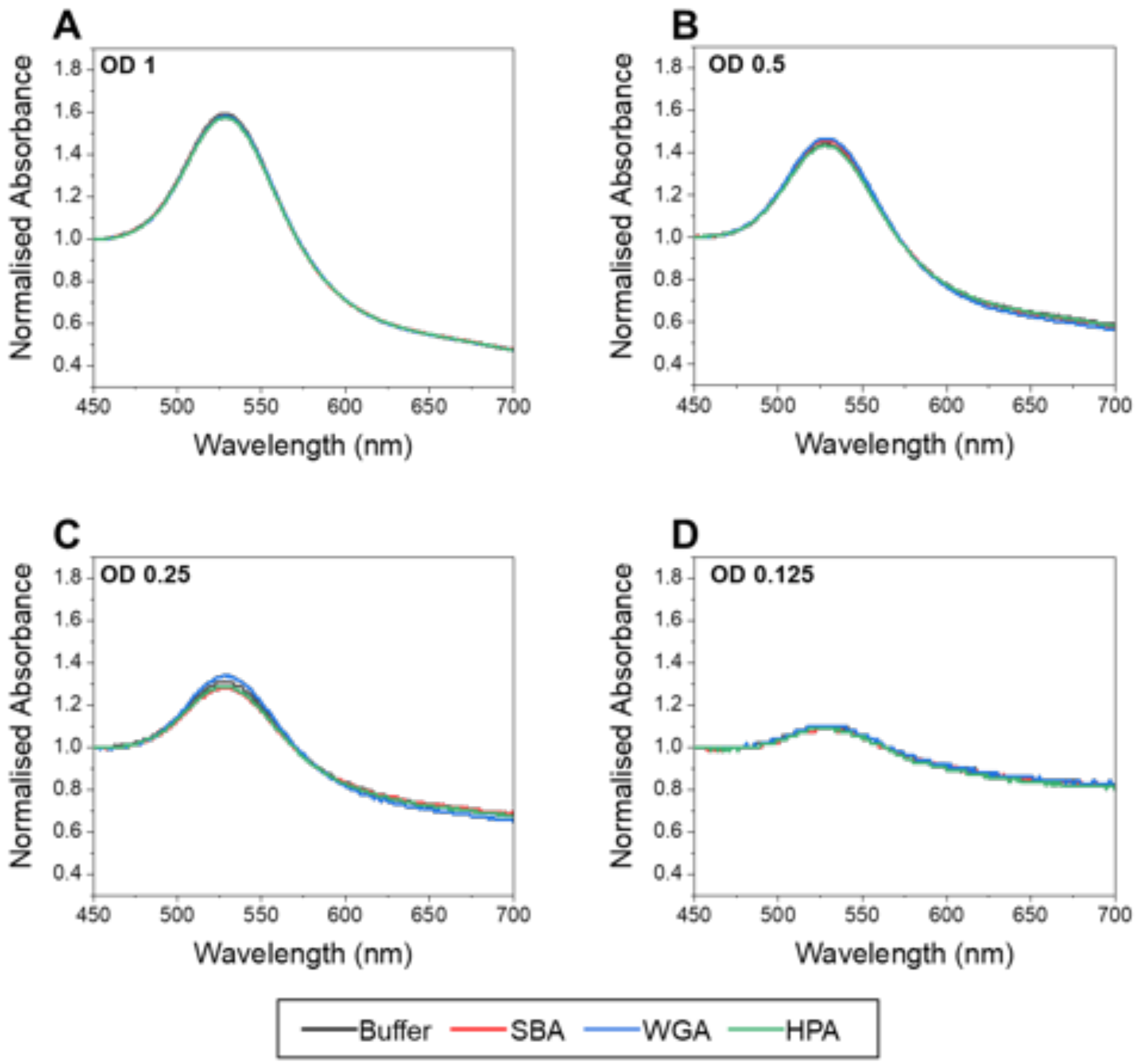

Figure S42. UV-Visible spectra of Gal-pHEA$_{40}$@AuNP$_{30}$ after addition of SBA, HPA or WGA. All lectins used at 0.1 mg.mL^{-1}. A) OD 1, B) OD 0.5, C) OD 0.25 and D) OD 0.125.

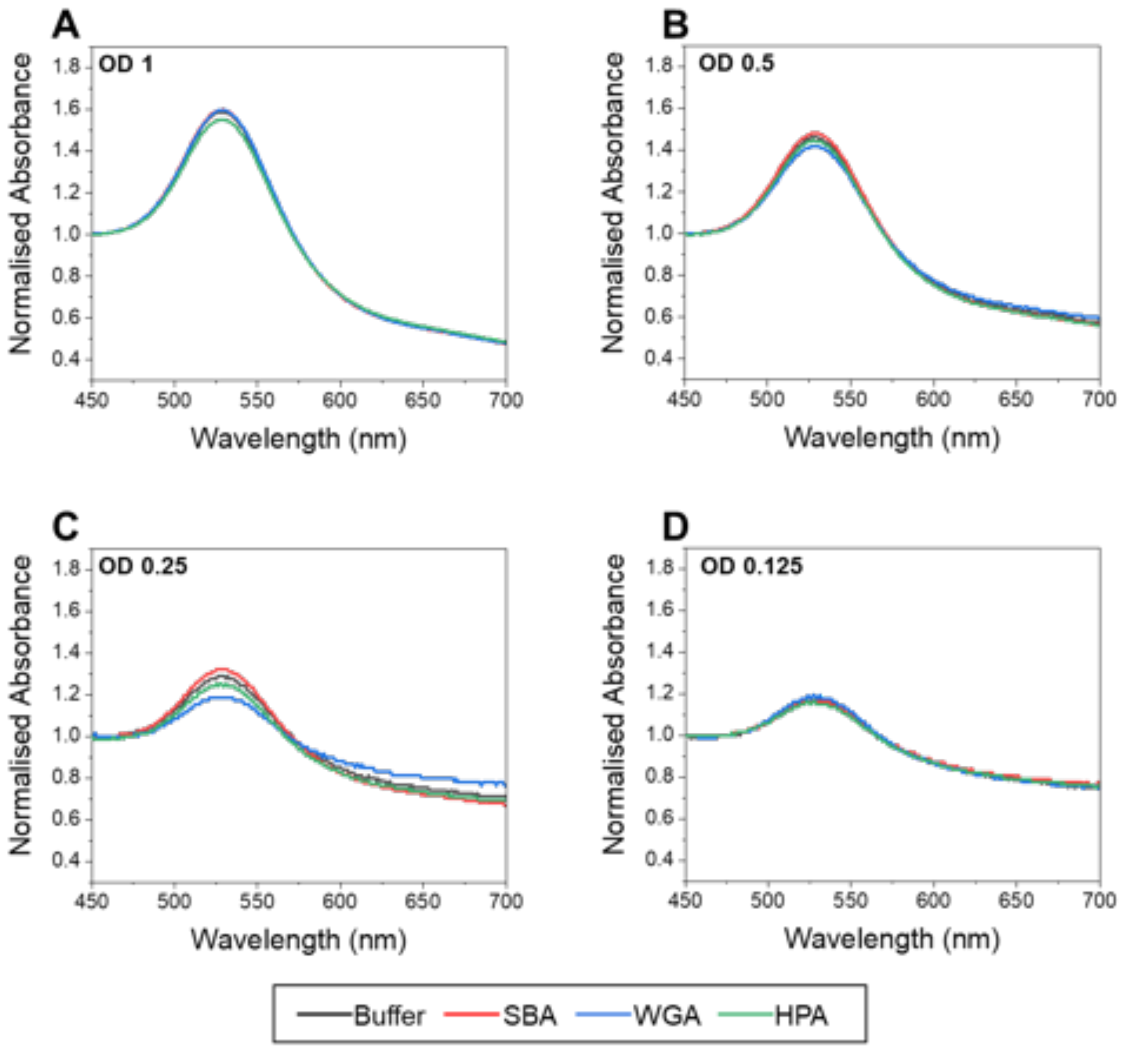

Figure S43. UV-Visible spectra of Gal-pHEA$_{74}$@AuNP$_{30}$ after addition of SBA, HPA or WGA. All lectins used at 0.1 mg.mL^{-1}. A) OD 1, B) OD 0.5, C) OD 0.25 and D) OD 0.125.

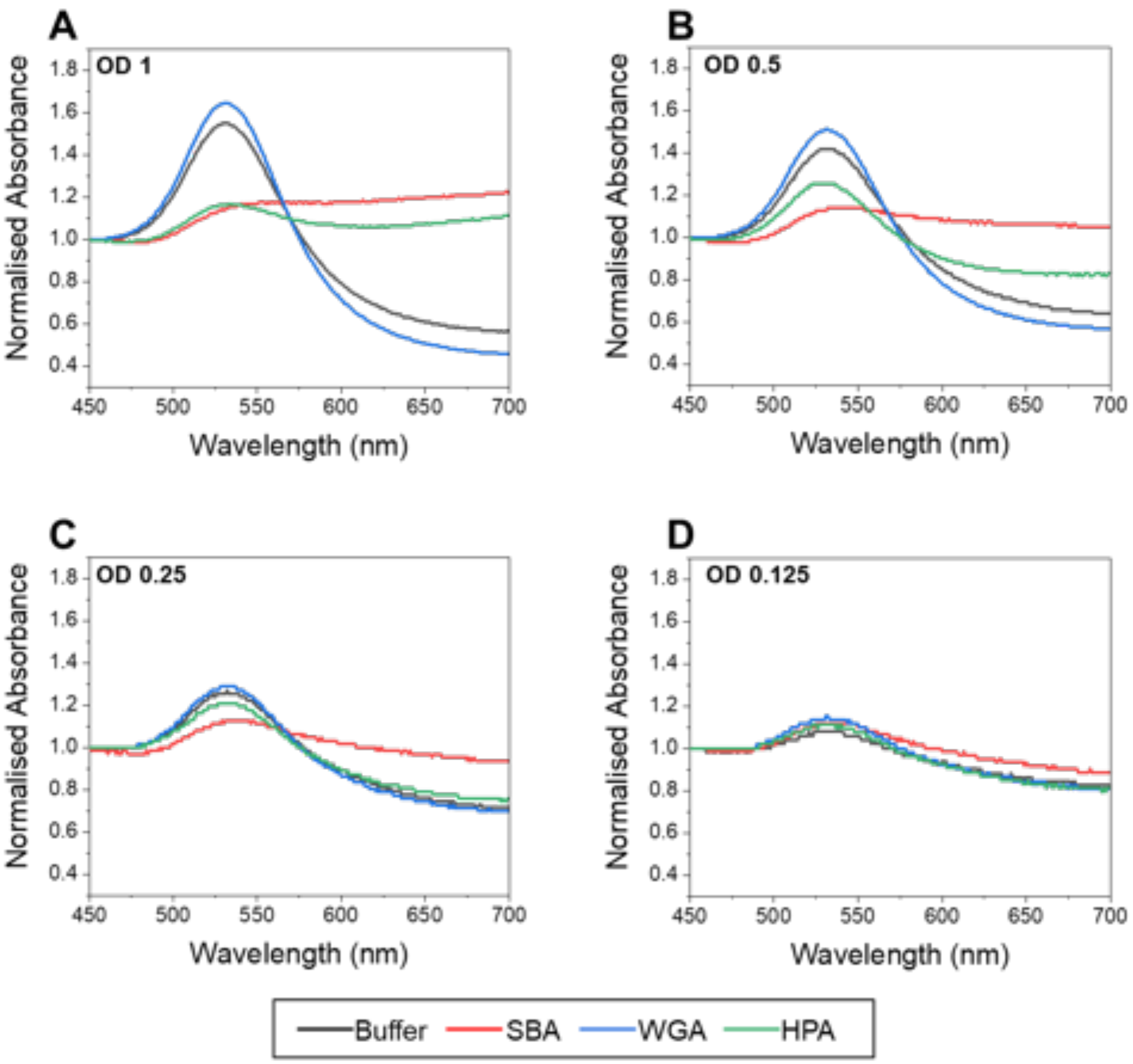

Figure S44. UV-Visible spectra of Gal-pHEA$_{33}$@AuNP$_{39}$ after addition of SBA, HPA or WGA. All lectins used at 0.1 mg.mL^{-1}. A) OD 1, B) OD 0.5, C) OD 0.25 and D) OD 0.125.

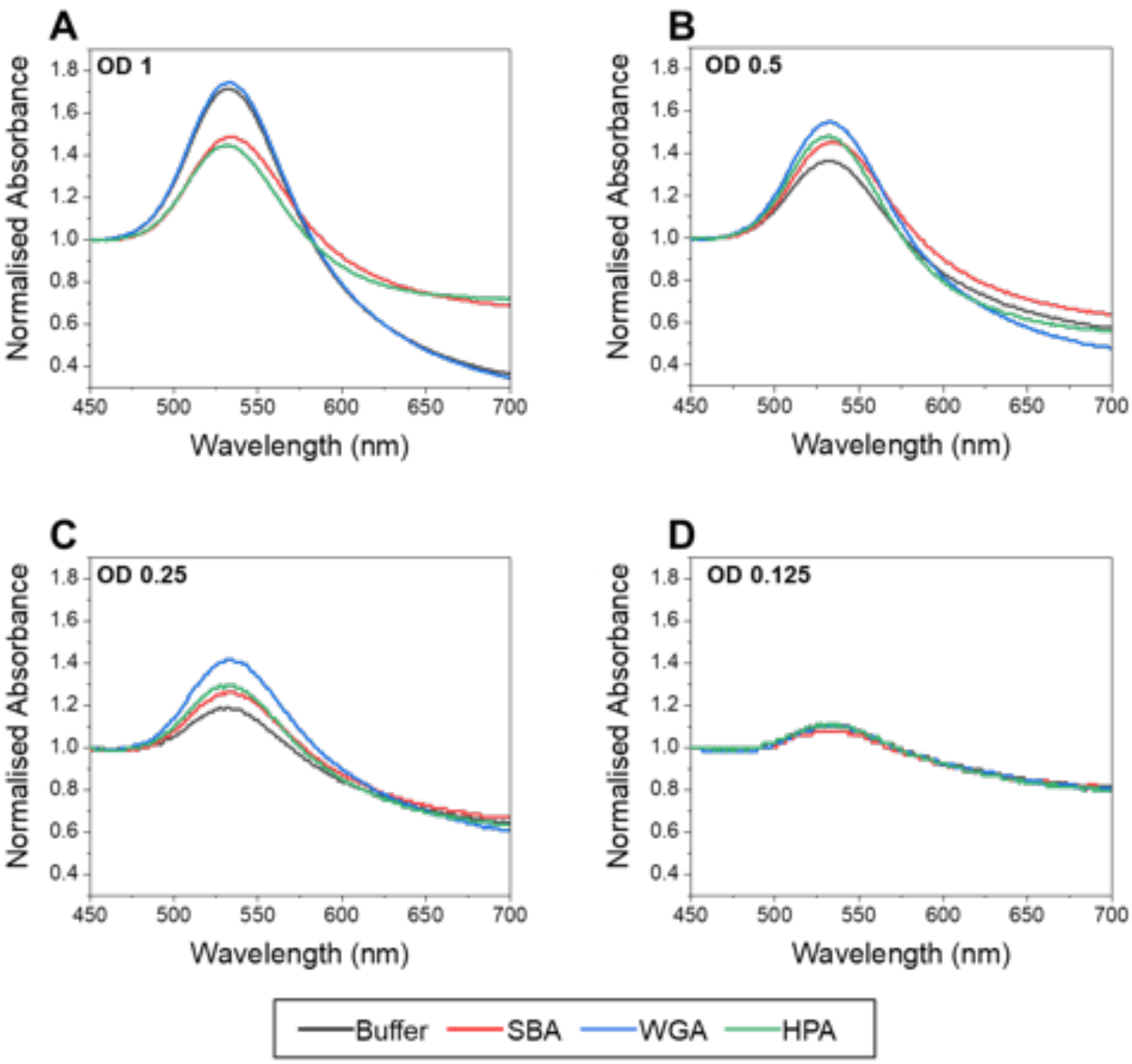

Figure S45. UV-Visible spectra of Gal-pHEA$_{40}$@AuNP$_{39}$ after addition of SBA, HPA or WGA. All lectins used at 0.1 mg.mL^{-1}. A) OD 1, B) OD 0.5, C) OD 0.25 and D) OD 0.125.

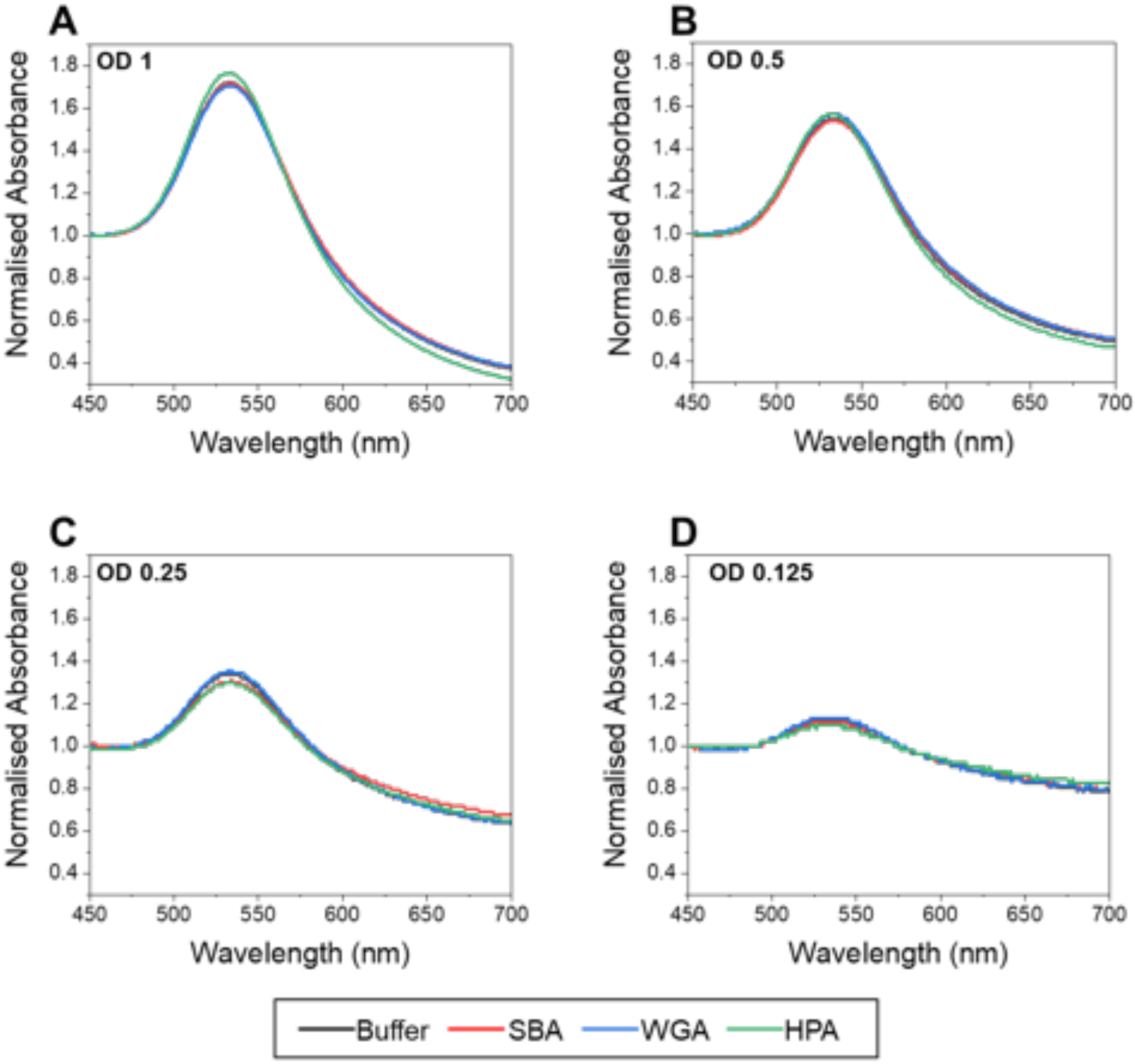

Figure S46. UV-Visible spectra of Gal-pHEA$_{74}$@AuNP$_{39}$ after addition of SBA, HPA or WGA. All lectins used at 0.1 mg.mL^{-1}. A) OD 1, B) OD 0.5, C) OD 0.25 and D) OD 0.125.

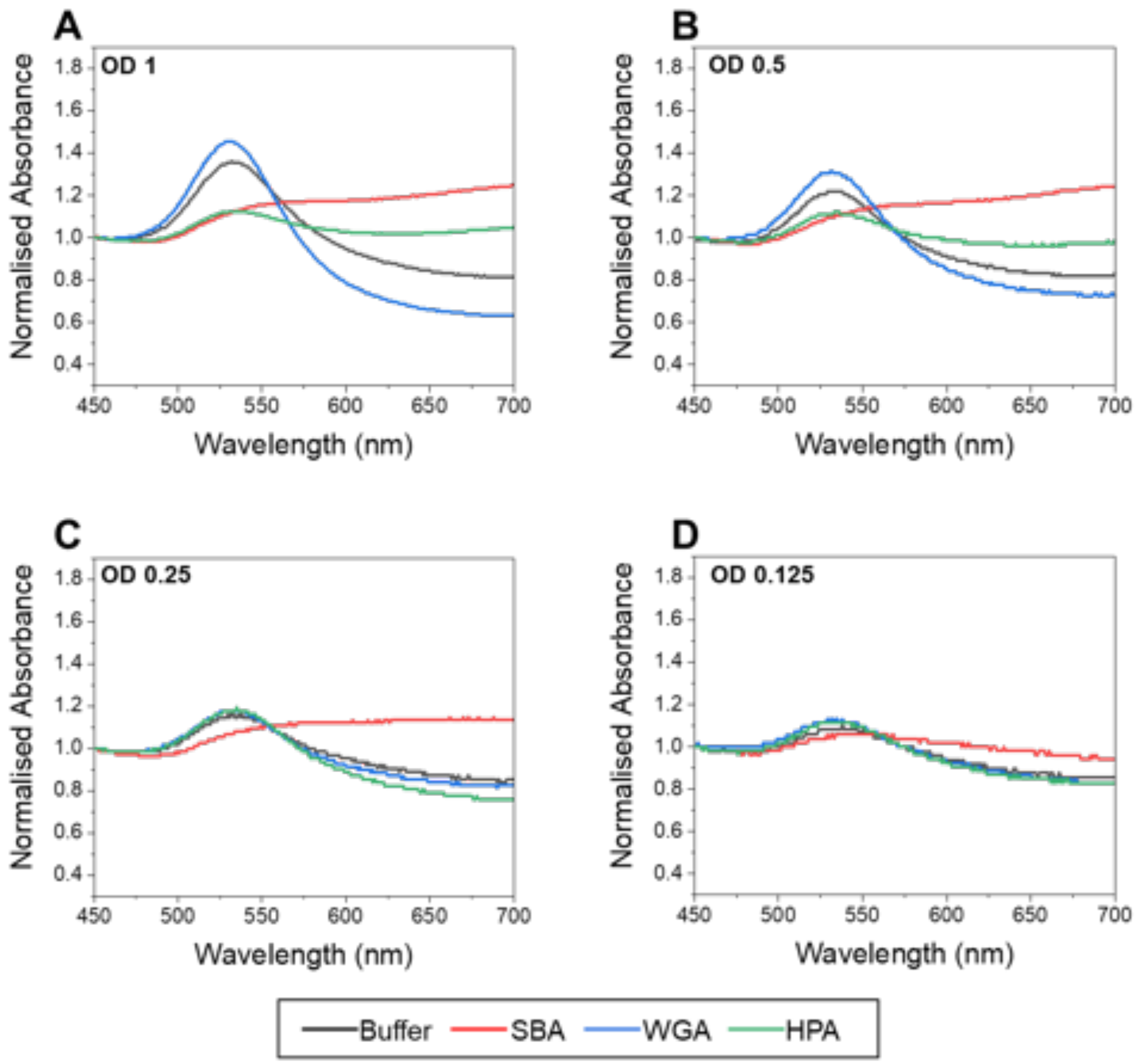

Figure S47. UV-Visible spectra of Gal-pHEA$_{33}$@AuNP$_{56}$ after addition of SBA, HPA or WGA. All lectins used at 0.1 mg.mL^{-1}. A) OD 1, B) OD 0.5, C) OD 0.25 and D) OD 0.125.

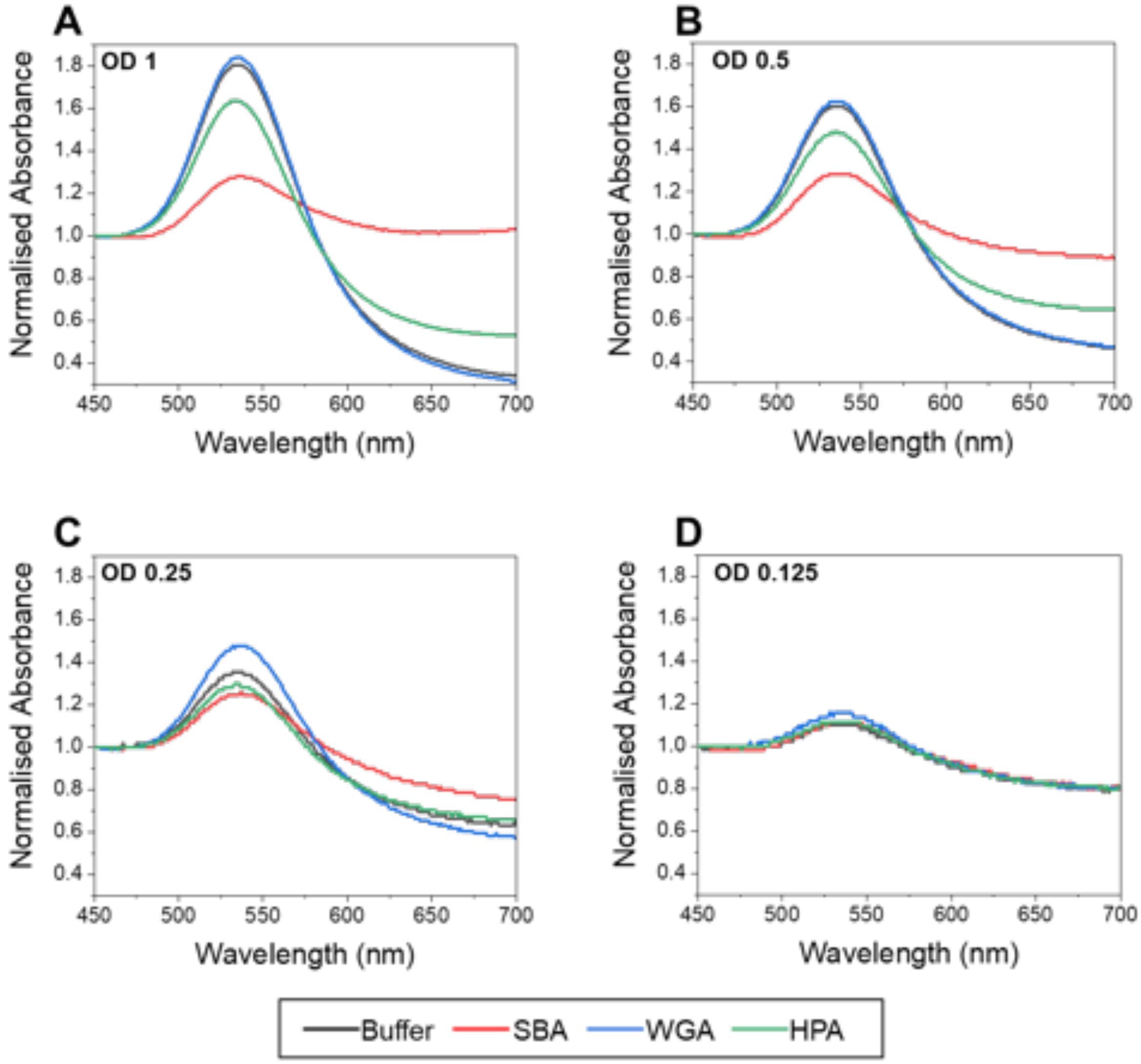

Figure S48. UV-Visible spectra of Gal-pHEA$_{40}$@AuNP$_{56}$ after addition of SBA, HPA or WGA. All lectins used at 0.1 mg.mL^{-1}. A) OD 1, B) OD 0.5, C) OD 0.25 and D) OD 0.125.

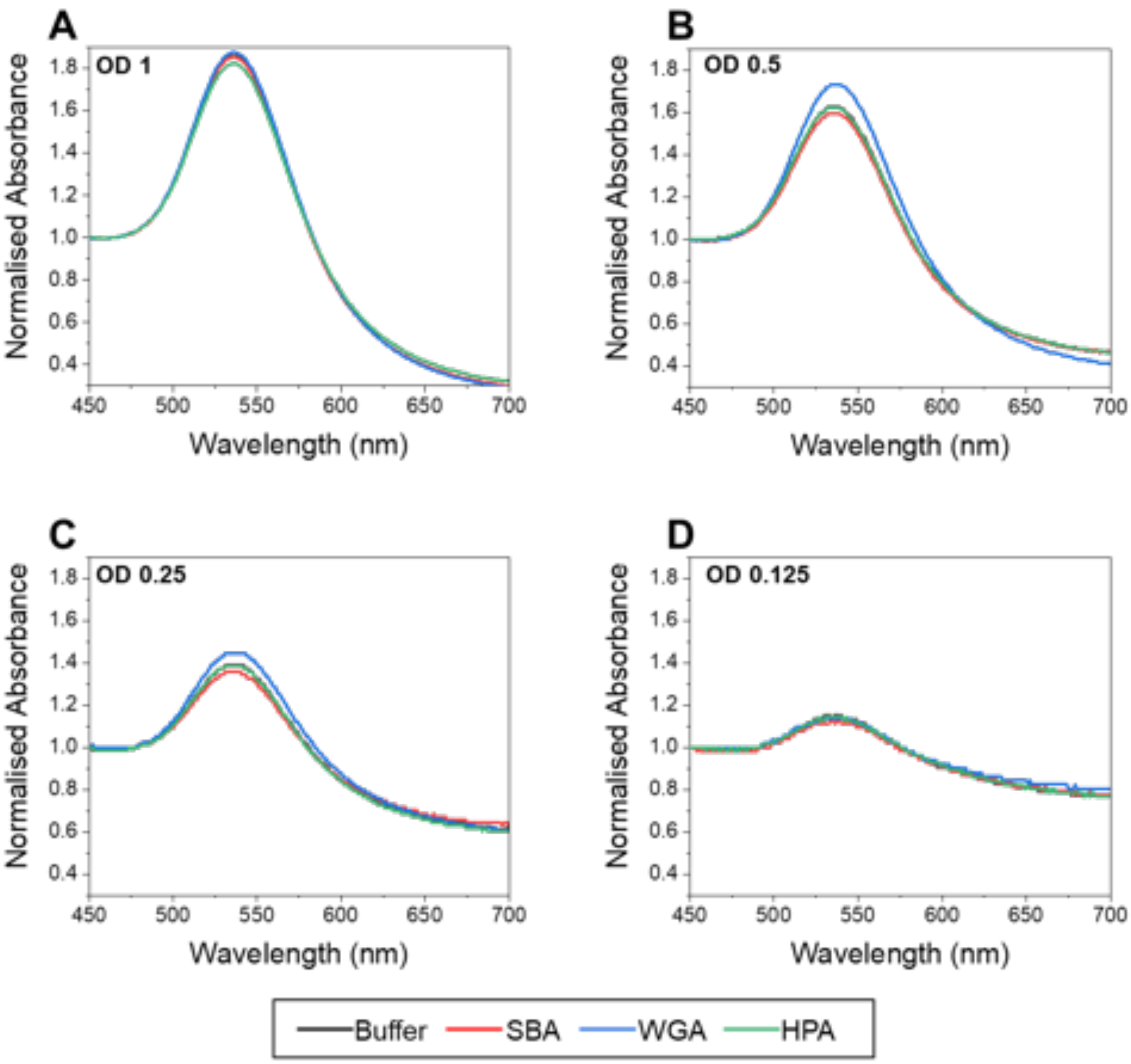

Figure S49. UV-Visible spectra of Gal-pHEA$_{74}$@AuNP$_{56}$ after addition of SBA, HPA or WGA. All lectins used at 0.1 mg.mL^{-1}. A) OD 1, B) OD 0.5, C) OD 0.25 and D) OD 0.125.

Aggregation Assay Heatmaps

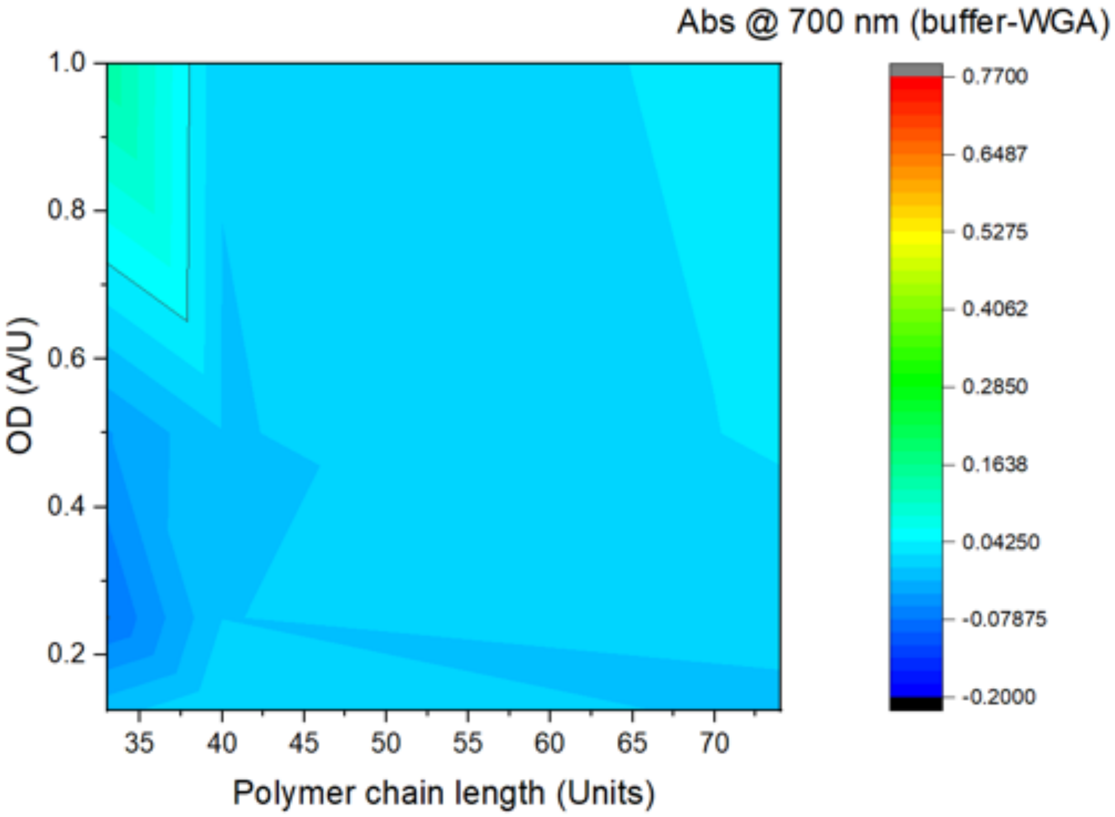

Figure S50. Heatmap showing how the difference in absorbance at 700 nm of buffer and WGA (Z) varies with polymer chain length (X) and optical density of the AuNPs (Y) for 17 nm AuNPs.

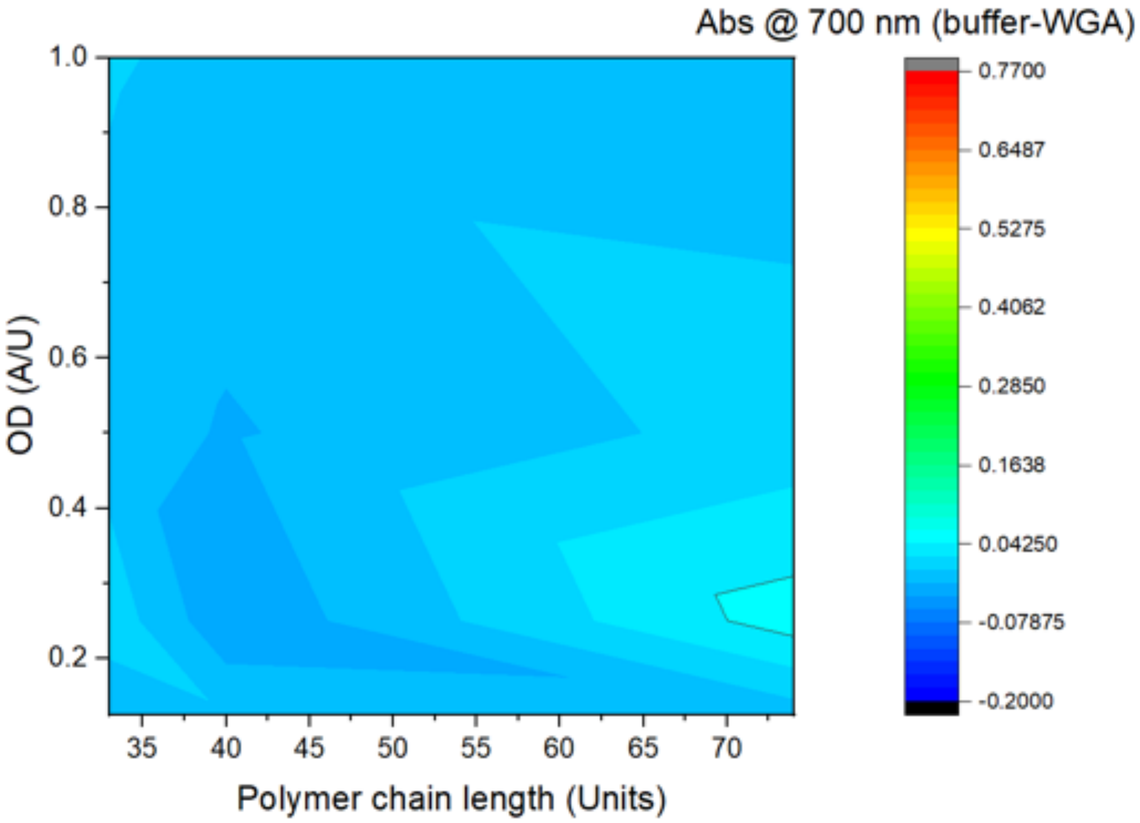

Figure S51. Heatmap showing how the difference in absorbance at 700 nm of buffer and WGA (Z) varies with polymer chain length (X) and optical density of the AuNPs (Y) for 30 nm AuNPs.

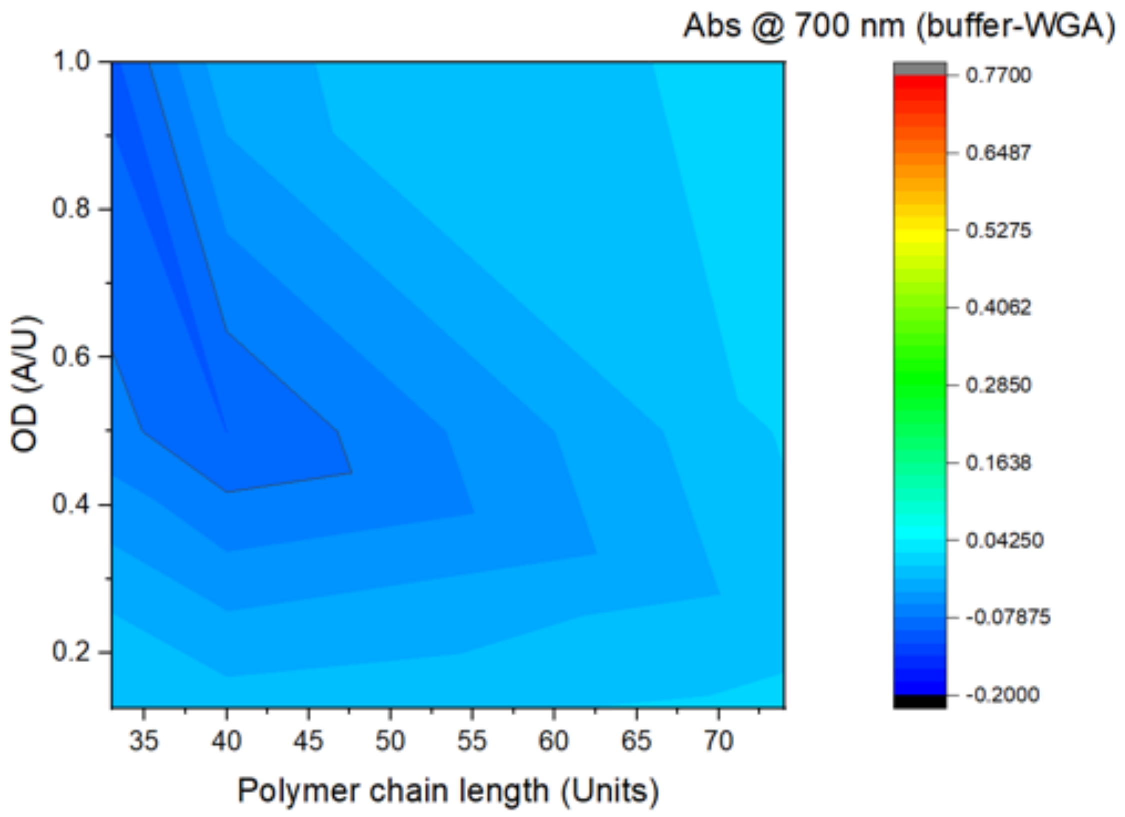

Figure S52. Heatmap showing how the difference in absorbance at 700 nm of buffer and WGA (Z) varies with polymer chain length (X) and optical density of the AuNPs (Y) for 39 nm AuNPs.

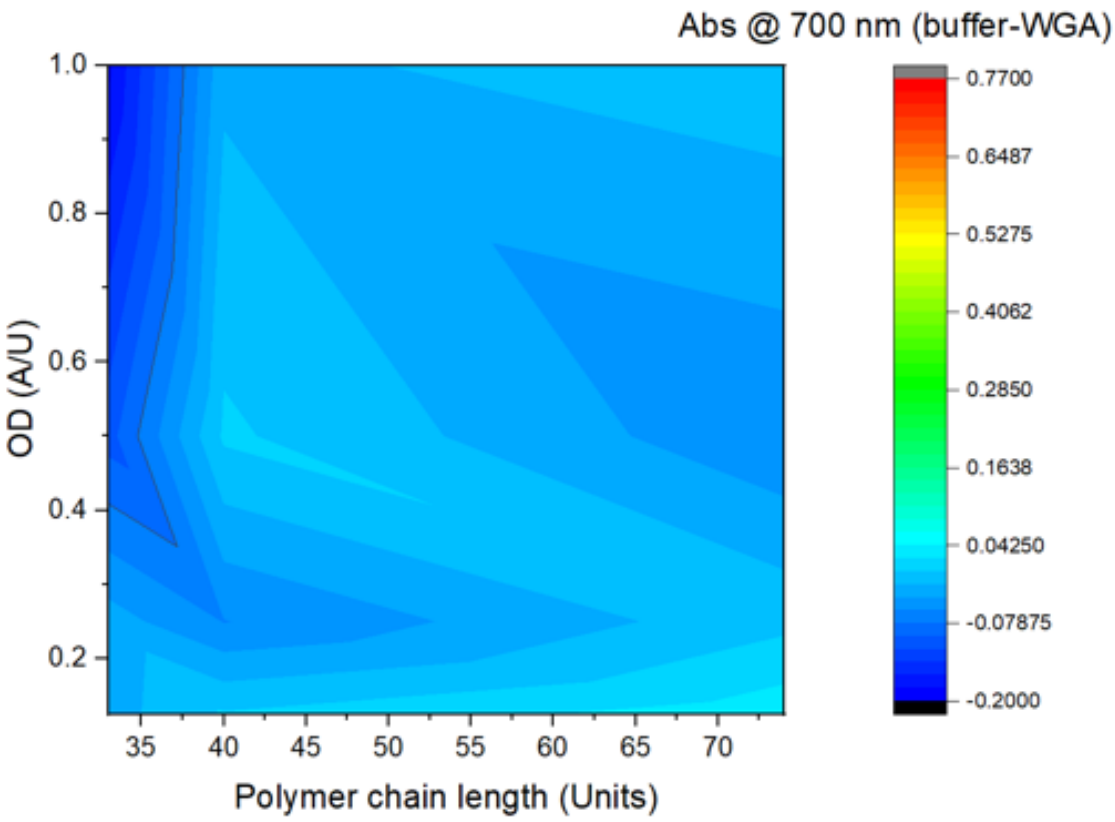

Figure S53. Heatmap showing how the difference in absorbance at 700 nm of buffer and WGA (Z) varies with polymer chain length (X) and optical density of the AuNPs (Y) for 56 nm AuNPs.

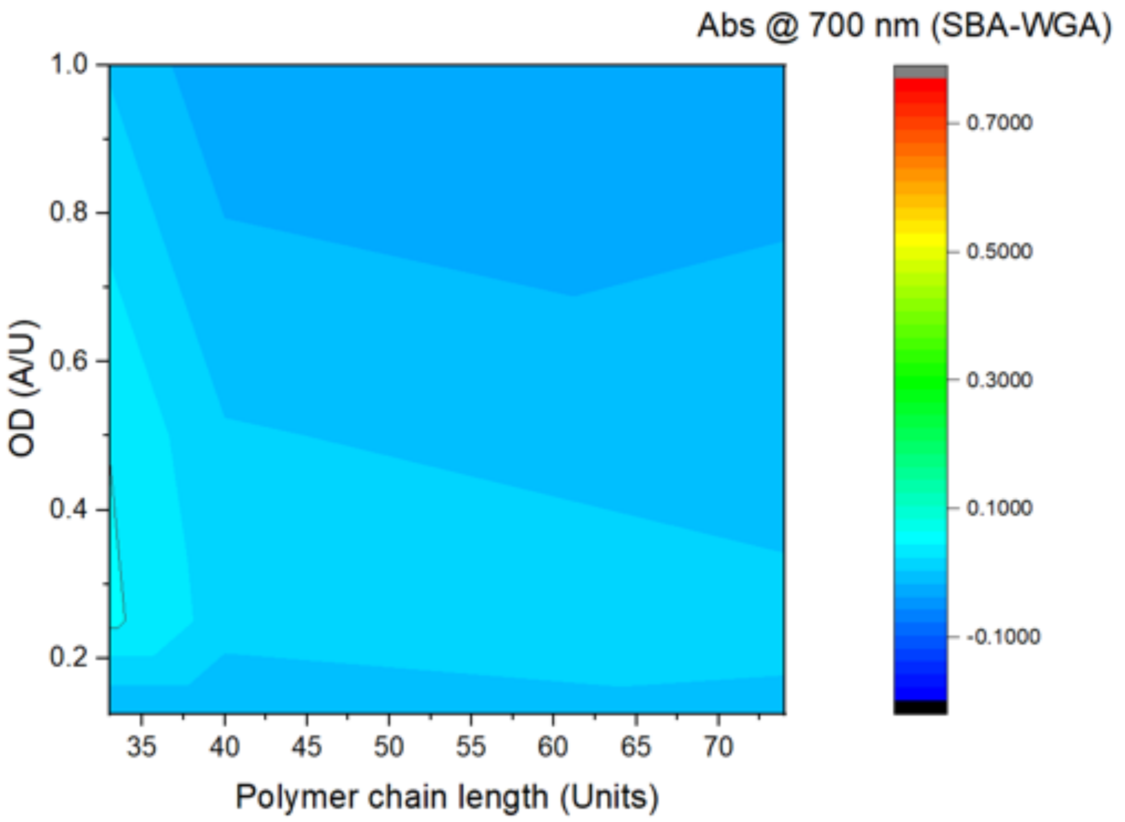

Figure S54. Heatmap showing how the difference in absorbance at 700 nm of SBA and WGA (Z) varies with polymer chain length (X) and optical density of the AuNPs (Y) for 17 nm AuNPs.

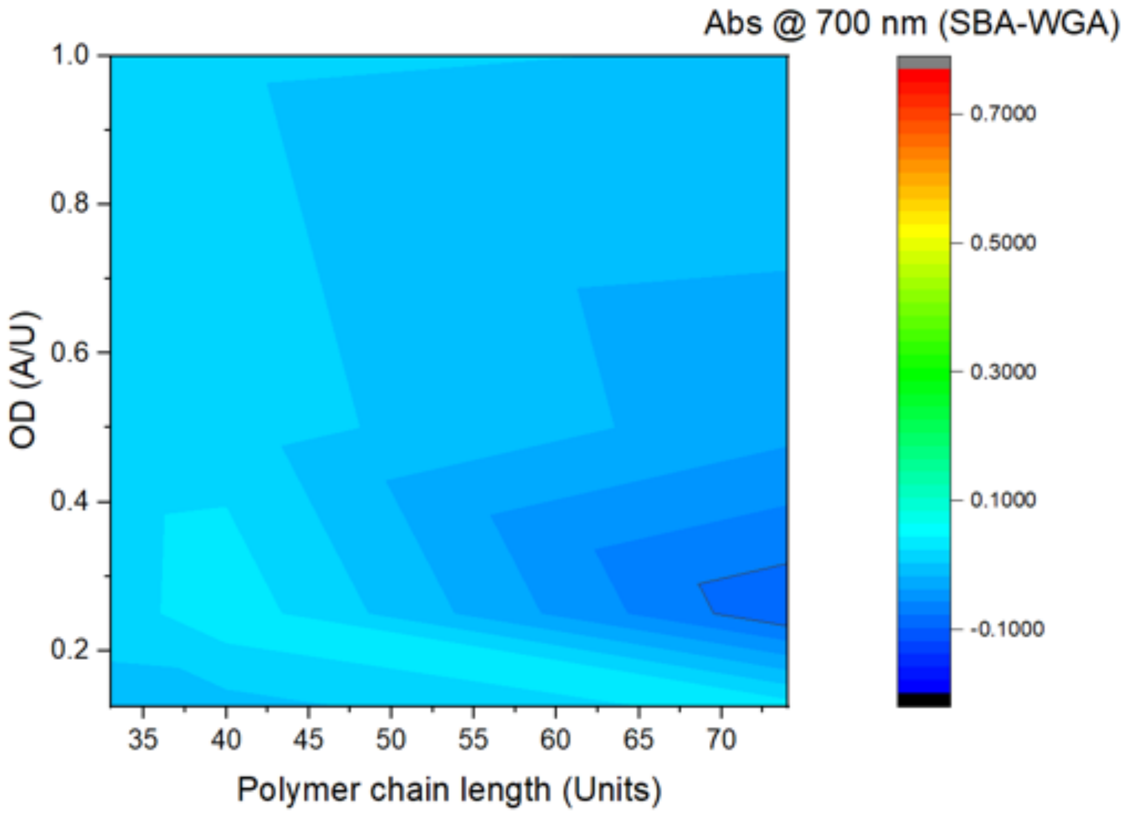

Figure S55. Heatmap showing how the difference in absorbance at 700 nm of SBA and WGA (Z) varies with polymer chain length (X) and optical density of the AuNPs (Y) for 30 nm AuNPs.

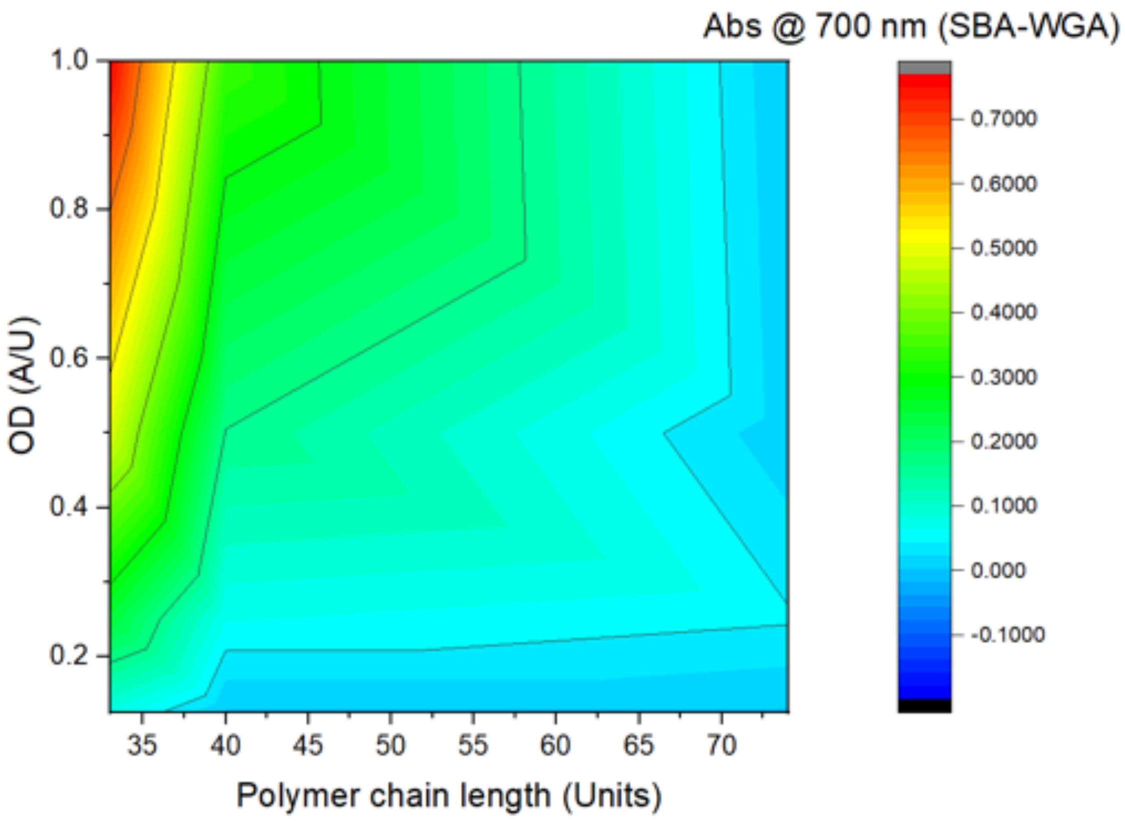

Figure S56. Heatmap showing how the difference in absorbance at 700 nm of SBA and WGA (Z) varies with polymer chain length (X) and optical density of the AuNPs (Y) for 39 nm AuNPs.

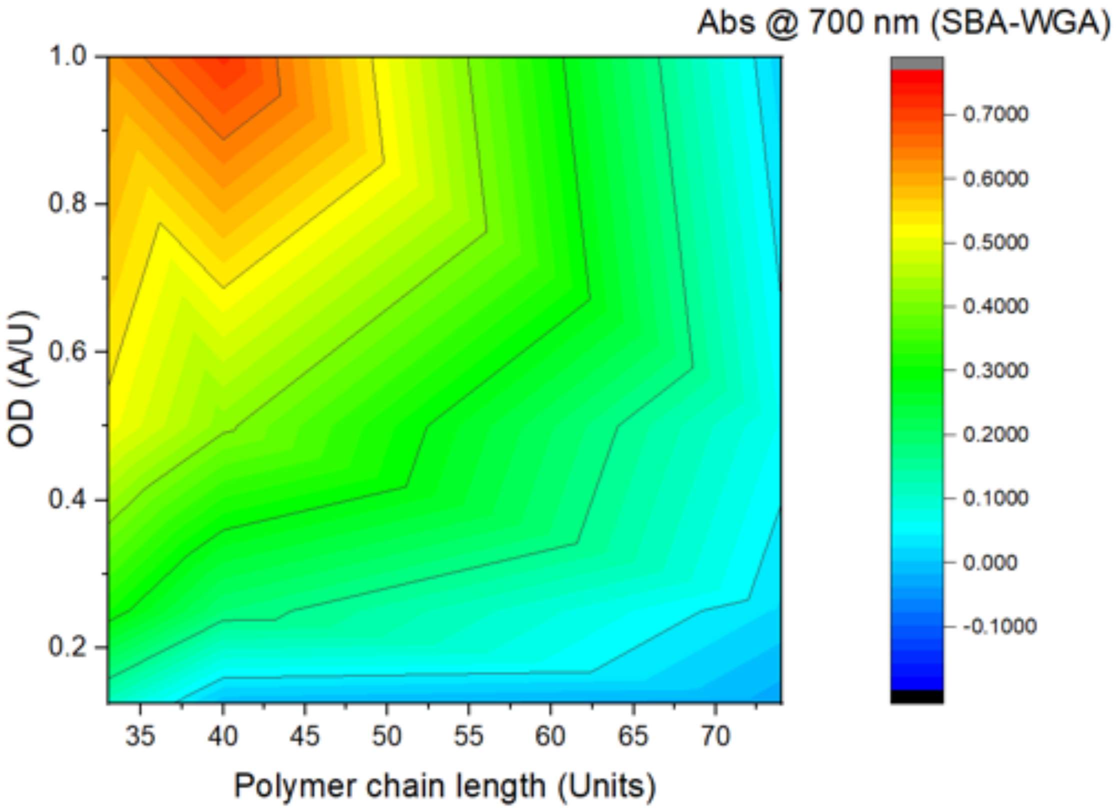

Figure S57. Heatmap showing how the difference in absorbance at 700 nm of SBA and WGA (Z) varies with polymer chain length (X) and optical density of the AuNPs (Y) for 56 nm AuNPs.

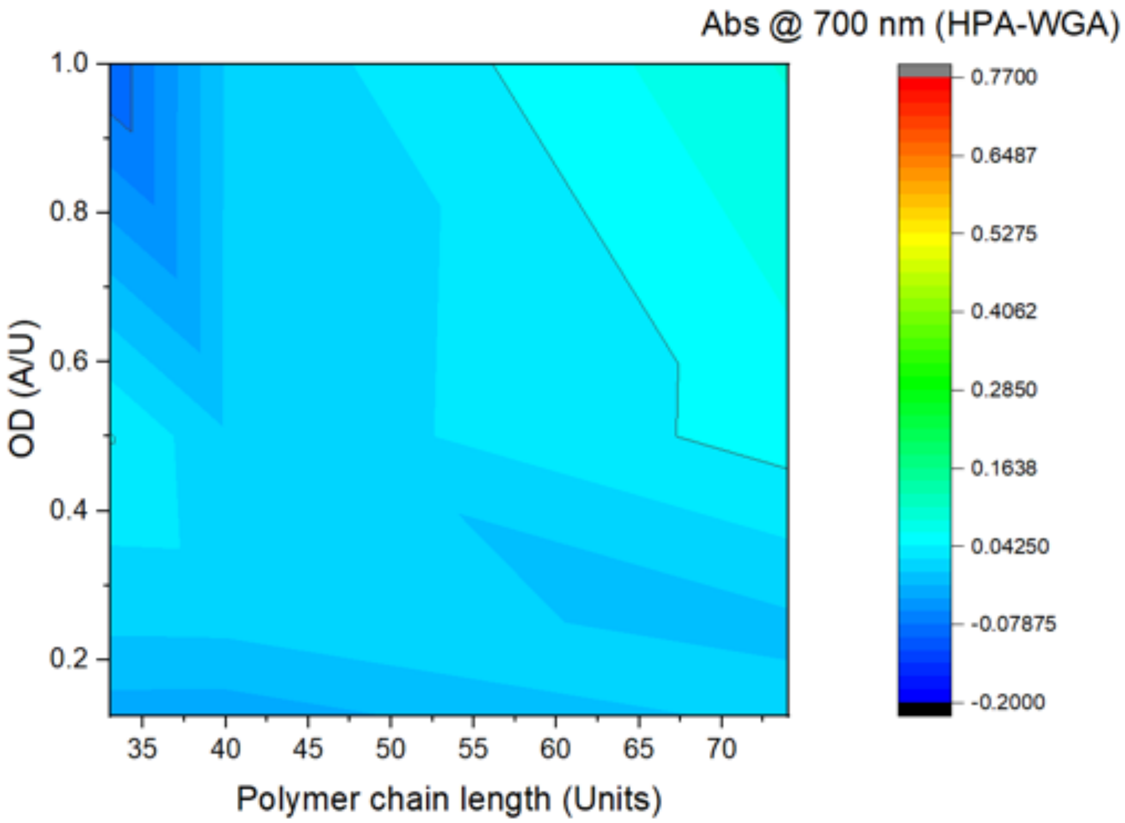

Figure S58. Heatmap showing how the difference in absorbance at 700 nm of HPA and WGA (Z) varies with polymer chain length (X) and optical density of the AuNPs (Y) for 17 nm AuNPs.

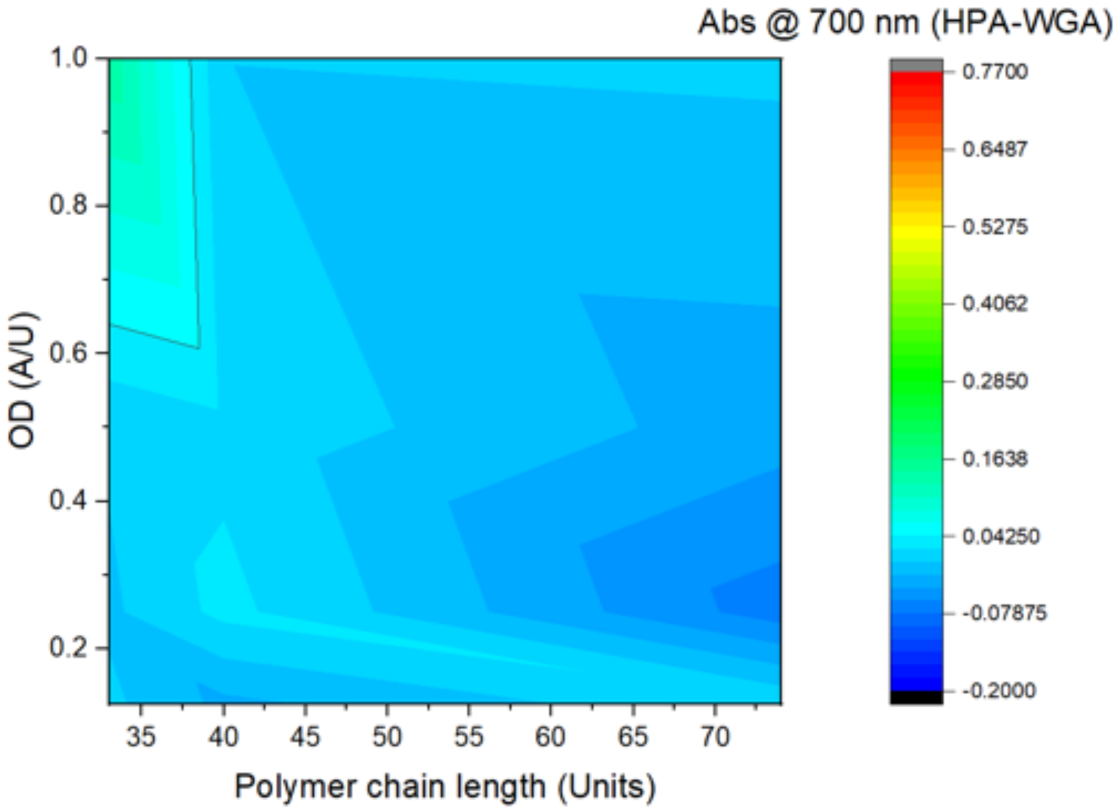

Figure S59. Heatmap showing how the difference in absorbance at 700 nm of HPA and WGA (Z) varies with polymer chain length (X) and optical density of the AuNPs (Y) for 30 nm AuNPs.

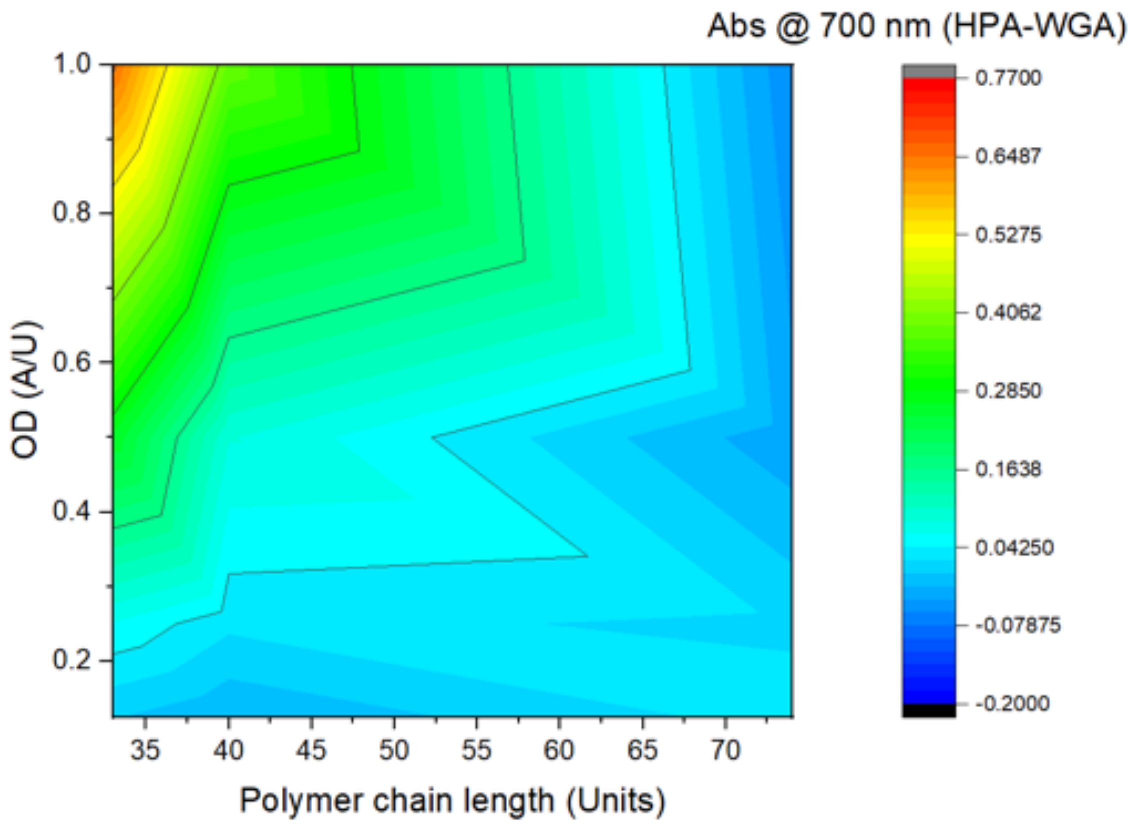

Figure S60. Heatmap showing how the difference in absorbance at 700 nm of HPA and WGA (Z) varies with polymer chain length (X) and optical density of the AuNPs (Y) for 39 nm AuNPs.

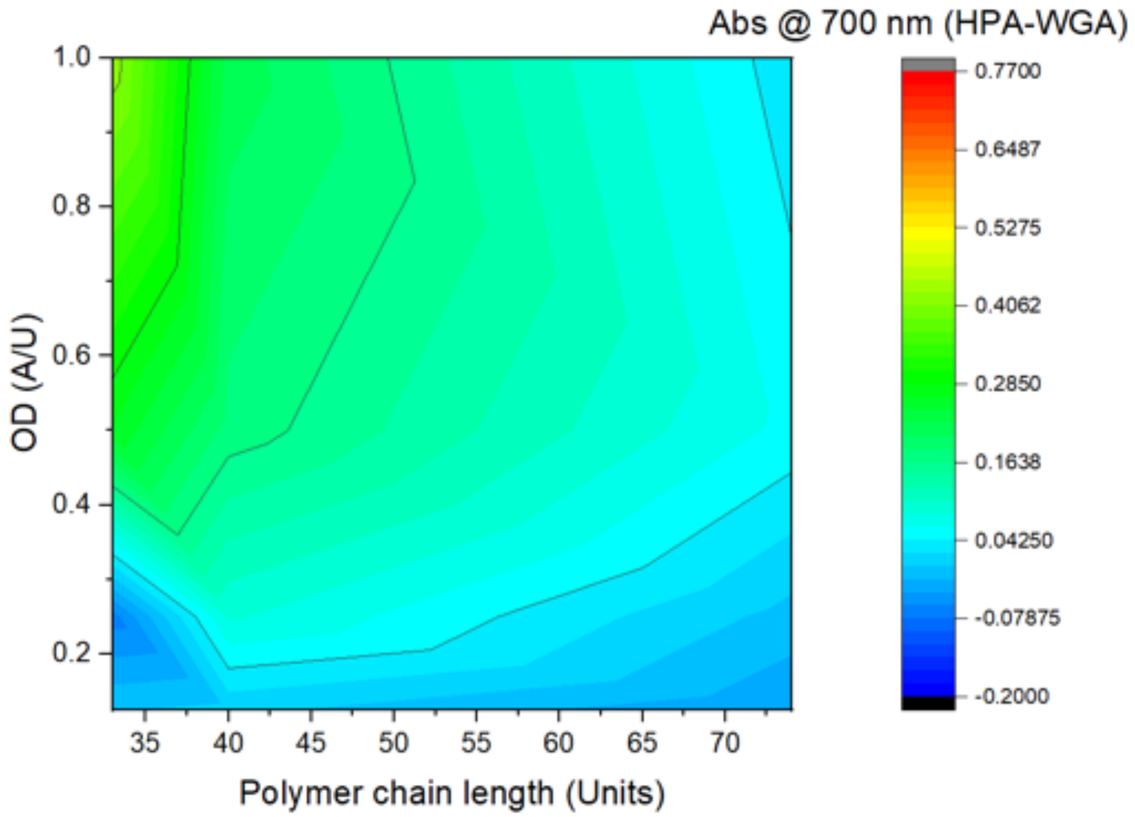

Figure S61. Heatmap showing how the difference in absorbance at 700 nm of HPA and WGA (Z) varies with polymer chain length (X) and optical density of the AuNPs (Y) for 56 nm AuNPs.

References

(1) Richards, S.-J.; Gibson, M. I. Optimization of the Polymer Coating for Glycosylated Gold Nanoparticle Biosensors to Ensure Stability and Rapid Optical Readouts. *ACS Macro Letters* **2014**, *3* (10), 1004–1008.

(2) Baker, A. N.; Richards, S.-J.; Guy, C. S.; Congdon, T. R.; Hasan, M.; Zwetsloot, A. J.; Gallo, A.; Lewandowski, J. R.; Stansfeld, P. J.; Straube, A.; Walker, M.; Chessa, S.; Pergolizzi, G.; Dedola, S.; Field, R. A.; Gibson, M. I. The SARS-COV-2 Spike Protein Binds Sialic Acids and Enables Rapid Detection in a Lateral Flow Point of Care Diagnostic Device. *ACS Central Science* **2020**, *6* (11), 2046–2052.

(3) Bastús, N. G.; Comenge, J.; Puntes, V. Kinetically Controlled Seeded Growth Synthesis of Citrate-Stabilized Gold Nanoparticles of up to 200 Nm: Size Focusing versus Ostwald Ripening. *Langmuir* **2011**, *27* (17), 11098–11105.

(4) Haiss, W.; Thanh, N. T. K.; Aveyard, J.; Fernig, D. G. Determination of Size and Concentration of Gold Nanoparticles from UV - Vis Spectra. *Analytical Chemistry* **2007**, *79* (11), 4215–4221.
